# Translational and post-translational control of human naïve versus primed pluripotency

**DOI:** 10.1016/j.isci.2021.103645

**Published:** 2021-12-17

**Authors:** Cheng Chen, Xiaobing Zhang, Yisha Wang, Xinyu Chen, Wenjie Chen, Songsong Dan, Shiqi She, Weiwei Hu, Jie Dai, Jianwen Hu, Qingyi Cao, Qianyu Liu, Yinghua Huang, Baoming Qin, Bo Kang, Ying-Jie Wang

**Affiliations:** 1 State Key Laboratory for Diagnosis and Treatment of Infectious Diseases, National Clinical Research Center for Infectious Diseases, Collaborative Innovation Center for Diagnosis and Treatment of Infectious Diseases, The First Affiliated Hospital, School of Medicine, Zhejiang University, Hangzhou, Zhejiang 310003, China; 2 Shaoxing People's Hospital, Shaoxing Hospital, Zhejiang University School of Medicine, Shaoxing, Zhejiang 312000, China; 3 Zhejiang Museum of Natural History, Hangzhou, Zhejiang 310014, China; 4 Shanghai Bioprofile Technology Co., Ltd., Shanghai 200241, China; 5 College of Life Sciences, Zhejiang University, Hangzhou, Zhejiang 310058, China; 6 Laboratory of Metabolism and Cell Fate, Center for Cell Lineage and Development, Guangzhou Institutes of Biomedicine and Health, Chinese Academy of Sciences, Guangzhou 510530, China; 7 Cancer Center, Zhejiang University, Hangzhou, Zhejiang 310058, China

**Keywords:** Molecular biology, Stem cells research, Proteomics

## Abstract

Deciphering the regulatory network for human naive and primed pluripotency is of fundamental theoretical and applicable significance. Here, by combining quantitative proteomics, phosphoproteomics, and acetylproteomics analyses, we revealed RNA processing and translation as the most differentially regulated processes between naive and primed human embryonic stem cells (hESCs). Although glycolytic primed hESCs rely predominantly on the eukaryotic initiation factor 4E (eIF4E)-mediated cap-dependent pathway for protein translation, naive hESCs with reduced mammalian target of rapamycin complex (mTORC1) activity are more tolerant to eIF4E inhibition, and their bivalent metabolism allows for translating selective mRNAs via both eIF4E-dependent and eIF4E-independent/eIF4A2-dependent pathways to form a more compact naive proteome. Globally up-regulated proteostasis and down-regulated post-translational modifications help to further refine the naive proteome that is compatible with the more rapid cycling of naive hESCs, where CDK1 plays an indispensable coordinative role. These findings may assist in better understanding the unrestricted lineage potential of naive hESCs and in further optimizing conditions for future clinical applications

## Highlights

• RNA processing and translation are most different between naive and primed hESCs
• Glycolytic primed hESCs mainly rely on eIF4E-dependent translation
• Bivalent metabolism in naive hESCs promotes eIF4E-independent translation
• CDK1 is required for naive pluripotency partially by activating E-cadherin signaling

## Graphical Abstract

**Figure:**
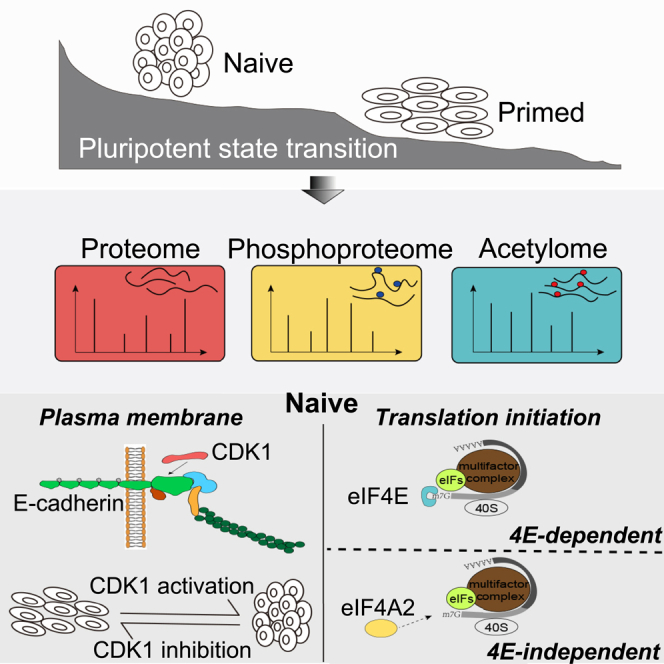

## Introduction

The successful isolation of inner cell mass (ICM) cells from blastocysts and propagating them in culture as embryonic stem cells (ESCs) offers exciting opportunities for studying early developmental mechanisms, drug screening, disease modeling, and cell-replacement therapies (Boroviak and Nichols, 2014; Ilic and Ogilvie, 2017). Unlike mouse ESCs (mESCs) that can be maintained in a naive ICM-like state in the presence of LIF (leukemia inhibitory factor) and 2i (MEK and GSK3 inhibitors) *in vitro* (Martello and Smith, 2014), human ESCs (hESCs) cultured in conventional conditions are in a so-called ‘‘primed’’ pluripotency that resembles the post-implantation epiblasts (Niakan et al., 2012). naive ESCs are characterized by dome-shaped colonies, dependence on JAK/STAT signaling, and increased homogeneity and single-cell survival while primed ESCs display flat colony morphology, dependence on TGFβ/activin signaling, and low single-cell clonogenicity (Nichols and Smith, 2009; Wu and Izpisua Belmonte, 2016).

To convert primed hESCs back to a naive state represents an appealing research endeavor that is of both theoretical and applicable importance. The naive hESCs have multiple advantageous features: they offer a unique model to study human preimplantation biology including the regulation of lineage decisions, responsiveness to signaling changes, genetic and epigenetic alterations induced by environmental cues; compared to primed hESCs, naive hESCs have higher homogeneity, pluripotency and self-renewal capacity that is expected to lead to more efficient directed differentiation toward germ layer derivatives (Collier and Rugg-Gunn, 2018; Wu and Izpisua Belmonte, 2016); in addition, naive hESCs also provide an opportunity for interspecies blastocyst complementation to produce transplantable patient-specific organs in large animal hosts (De Los Angeles et al., 2018). More excitingly, recent work from several laboratories revealed the capabilities of naive hESCs in generating trophectoderm and descendant trophoblast cell types and in forming three-dimensional blastocyst-like structures (Guo et al., 2021; Io et al., 2021; Yanagida et al., 2021), which will have a transformative impact on future human reproductive research.

Over the past years, by combining 2i/LIF with different components, several groups have formulated protocols to induce naive pluripotency in hESC, both from existing primed hESCs and by direct derivation from the blastocysts (Collier and Rugg-Gunn, 2018; Theunissen et al., 2014; Warrier et al., 2017). Although naïve-like hESCs derived from different induction conditions differ in some properties (Liu et al., 2017; Warrier et al., 2017), they generally share several key features: they display global DNA hypomethylation compared to primed hESCs; they have reduced levels of the repressive histone mark H3K27me3 at >3000 Polycomb-associated gene promoter; they exhibit the preferential activity of the OCT4 distal enhancer, whereas primed hESCs use the proximal enhancer; female naïve-like hESCs have two active X chromosomes with localized depletion of the H3K27me3 mark (Collier and Rugg-Gunn, 2018; Theunissen et al., 2016). Besides, several genes or cell surface proteins are considered as relatively specific biomarkers for naïve-like hESCs (Taei et al., 2020; Trusler et al., 2018; Weinberger et al., 2016). So far, most naïve-like hESCs are induced in the presence of feeder cells that usually are of murine origin (Collier and Rugg-Gunn, 2018) and the partially feeder-dependent PXGL hESC induction system derived from the t2iLGö system (Bredenkamp et al., 2019) is considered as one of the best systems that can induce the so-called “bona fide naive hESCs.” However, the presence of non-human factors in the culture may limit the use of hESCs for therapeutic purposes. Recent improvements in hESC culture have enabled the commercial development of completely defined, feeder-free culture conditions such as mTeSR1 for primed h

ESCs, and RSeT Feeder-Free Medium for naive hESCs that are highly tractable and have less abnormal karyotypes (Ward et al., 2017), addressing the challenge of delivering the clinical promise of hESCs and ultimately leading to Good Manufacturing Practice (GMP)-compatible services and products. More recently, another feeder-free naive hESC induction system (FINE) was developed that offered more robust naive marker expression including 8-cell stage-specific transcripts (Szczerbinska et al., 2019).

To further improve the induction and cultivation conditions for human naive pluripotency that can faithfully recapitulate the key attributes of ICM cells, it is important to gain deeper insights into the roles of the major signaling and metabolic pathways in the acquisition and maintenance of the different states of pluripotency. Corresponding to 2i, MEK/ERK and GSK3 signaling pathways are well established to be down-regulated in naive hESCs (Collier and Rugg-Gunn, 2018). Conversely, LIF/JAK/STAT3 (Chen et al., 2015), LPA/YAP (Qin et al., 2016), WNT, TGF-β pathways (Taei et al., 2020) are the main signaling pathways known to promote human naive pluripotency. These signaling pathways are expected to affect a battery of downstream proteins via multiple post-translational modifications (PTMs) such as phosphorylation, acetylation, ubiquitination, etc (Wang et al., 2014a), but a systematical comparison of PTM profiles between naive and primed hESCs is lacking. In recent years, there has been a growing appreciation that energy balance and metabolic status are closely associated with stem cell functions, and metabolic reprogramming and regulation have become one of the research focuses in stem cell biology (Harvey et al., 2019; Tsogtbaatar et al., 2020). Although consensus has been reached that the primed hESCs exclusively rely on glycolysis for energy supply and cell proliferation while naive hESCs utilize a bivalent metabolic system (i.e., both glycolysis and oxidative phosphorylation [OXPHOS]) (Tsogtbaatar et al., 2020), the potential functional roles and specific contributions of distinct metabolic modes in regulating naive versus primed pluripotency remain largely unknown. In this study, by multi-omics analyses, we identified CDK1 as a crucial kinase for maintaining human naive pluripotency, and revealed RNA processing and translation as the most differentially regulated processes between naive and primed hESCs. We further discovered that under stressed conditions, glycolytic primed hESCs rely predominantly on eukaryotic initiation factor 4E (eIF4E)-mediated cap-dependent pathway for protein translation, while bivalent metabolism in naive hESCs promotes both cap-dependent and eIF4A2-dependent/cap-independent translation that confer them higher capability for rapid cell cycling and propagation.

## Results

### Induction of primed human embryonic stem cells into naïve-like human embryonic stem cells

**Figure 1:**
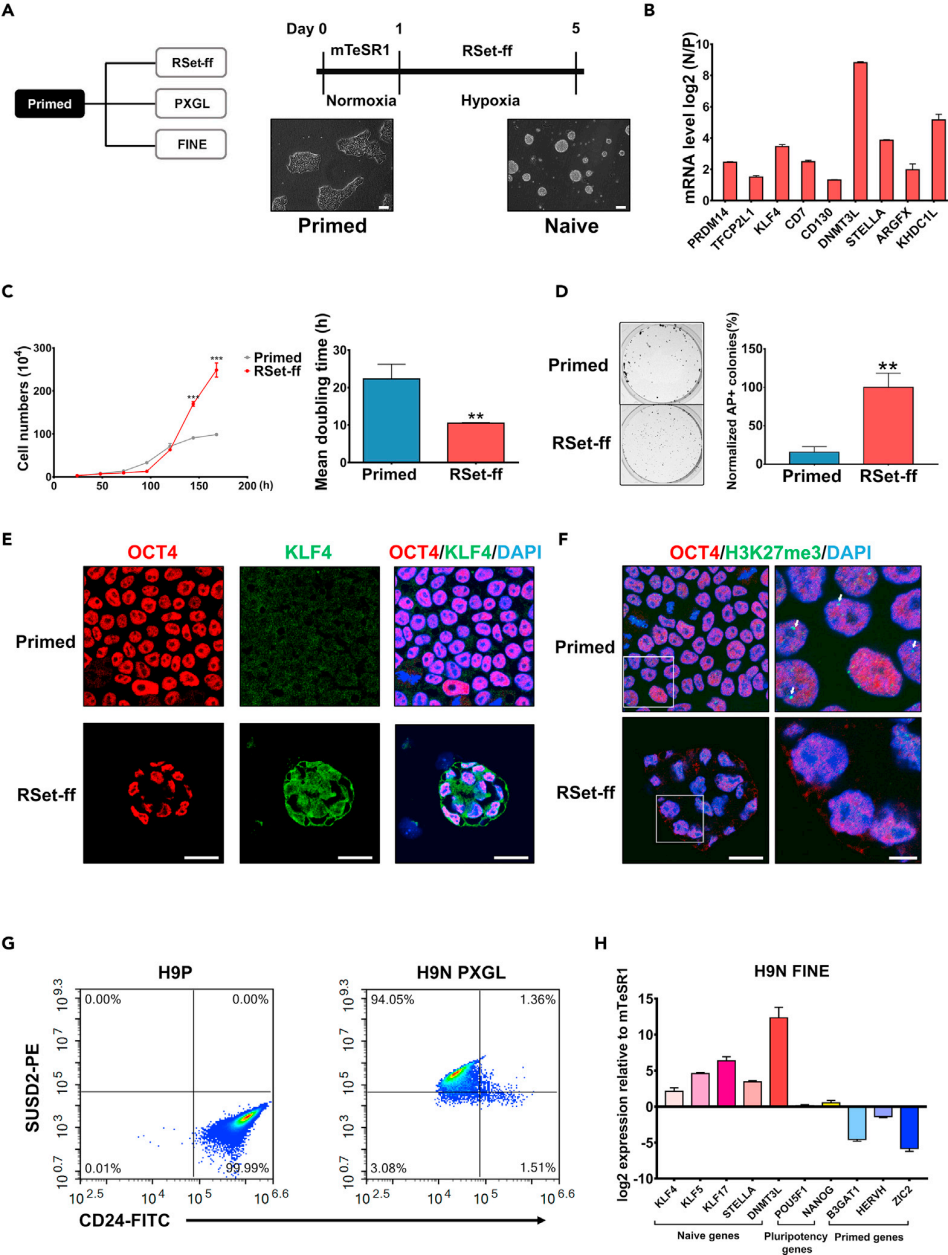
Induction of primed hESCs into naive pluripotency under three culture conditions (A) Overview for the induction of primed hESCs into naive hESCs via three culture systems. Flowchart detailing commercial RSet-feeder-free (RSet-ff) induction system, and light micrographs showing primed H9 hESCs and converted naive H9 hESCs. Scale bars, 100 μm. (B) Primed and RSet-ff-induced naive H9 hESCs were harvested, and the mRNA levels of naive-pluripotency marker genes were determined by qRT-PCR. The log2 naive/Primed fold change values were presented as mean ± S.D. of three independent experiments. (C) Mean population doubling time of primed versus RSet-ff-induced naive H9 hESCs. Total cell numbers (left panels) were counted every 24 h, and the mean population doubling times were calculated (right panels). The data were presented as the mean ± S.D. from three independent experiments Unpaired t test was performed so that ∗∗p < 0.01, ∗∗∗p < 0.001. (D) Single-cell clonogenicity of primed versus RSet-ff-induced naive H9 hESCs was determined by Alkaline Phosphatase Staining. The data were presented as the mean ± S.D. from three independent experiments. Unpaired t test was performed so that ∗∗p < 0.01. (E) Primed versus RSet-ff-induced naive H9 hESCs were fixed and immunostained for pluripotency marker OCT4 and naive-associated marker KLF4, and counterstained for nuclei. Scale bars, 50 μm. (F) Primed versus RSet-ff-induced naive H9 hESCs were fixed and immunostained for H3K27me3 foci (arrows), and counterstained for OCT4 and nuclei. Scale bars, 50 μm (left), 20 μm (right). (G) Flow cytometry analysis of SUSD2 and CD24 expression in primed versus PXGL-induced naive H9 hESCs. (H) Expression of naive, primed and pluripotency-associated transcripts in primed versus FINE-induced naive H9 hESCs. Data were presented as mean ± S.D. of three independent experiments.

Given its advantages mentioned above, in this study, we mainly employed the RSeT Feeder-Free Medium (designated as “RSet-ff”) (Ward et al., 2017) to induce naïve-like hESCs, where dome-shaped colonies of both H1 (Figure S1A) and H9 hESCs (Figure 1A) were formed within 5–7 days. Besides morphological features, examination of several cellular and molecular hallmarks for naive pluripotency (Collier and Rugg-Gunn, 2018; Taei et al., 2020) further demonstrated our success in acquiring the naïve-like hESCs (Figures 1B and S1B). RSet-ff naïve-like hESCs had a much shorter doubling time (Figures 1C and S1C) and enhanced single-cell clonogenicity (Figures 1D and S1D) than their primed counterparts. Furthermore, immunofluorescence microscopy confirmed a much higher expression level of the naive pluripotency marker KLF4 in RSet-ff naïve-like cells (Figures 1E and S1E). Consistent with the notion that female naive hESCs have two active X chromosomes (XaXa) while female primed hESCs undergo one X chromosome inactivation (XaXi) caused by H3K27me3, the H3K27me3 foci-positive nuclei were detected predominantly in primed but not naïve-like H9 hESCs (Figure 1F). In addition, the CpG methylation level at the DNMT3L promoter was reduced by nearly 35% (Figures S1F and S2C), indicating a global DNA hypomethylation state present in RSet-ff naïve-like hESCs.

We also induced naïve-like hESCs via another two approaches. The PXGL hESC induction system (Bredenkamp et al., 2019) (Figure S2A), one of the best systems that can induce the “bona fide naive hESCs” and further generate blastocyst trophectoderm (Guo et al., 2021), gave rise to characteristic SUSD2+/CD24 low or negative naïve-like hESCs that were unambiguously distinguished from their SUSD2-/CD24 + primed counterparts (Figure 1G). Remarkably, the extent of upregulation of the key naive genes and pluripotency genes over primed hESCs were rather similar between the PXGL- and RSet-ff-induced naïve-like hESCs (Figure S2D). Likewise, the stabilized (i.e., passage 5 onward) naïve-like hESCs derived from the FINE feeder-free naive induction system (Szczerbinska et al., 2019) (Figure S2B) exhibited significantly upregulated expression of naive genes over primed hESCs (Figure 1H).

Collectively, we established three tractable, reliable, and efficient systems to induce naïve-like hESCs (hereafter referred to as naive hESCs) that can be used for further investigations.

### Global profiles of proteome, acetylome, and phosphoproteome in primed and naïve human embryonic stem cells

**Figure 2:**
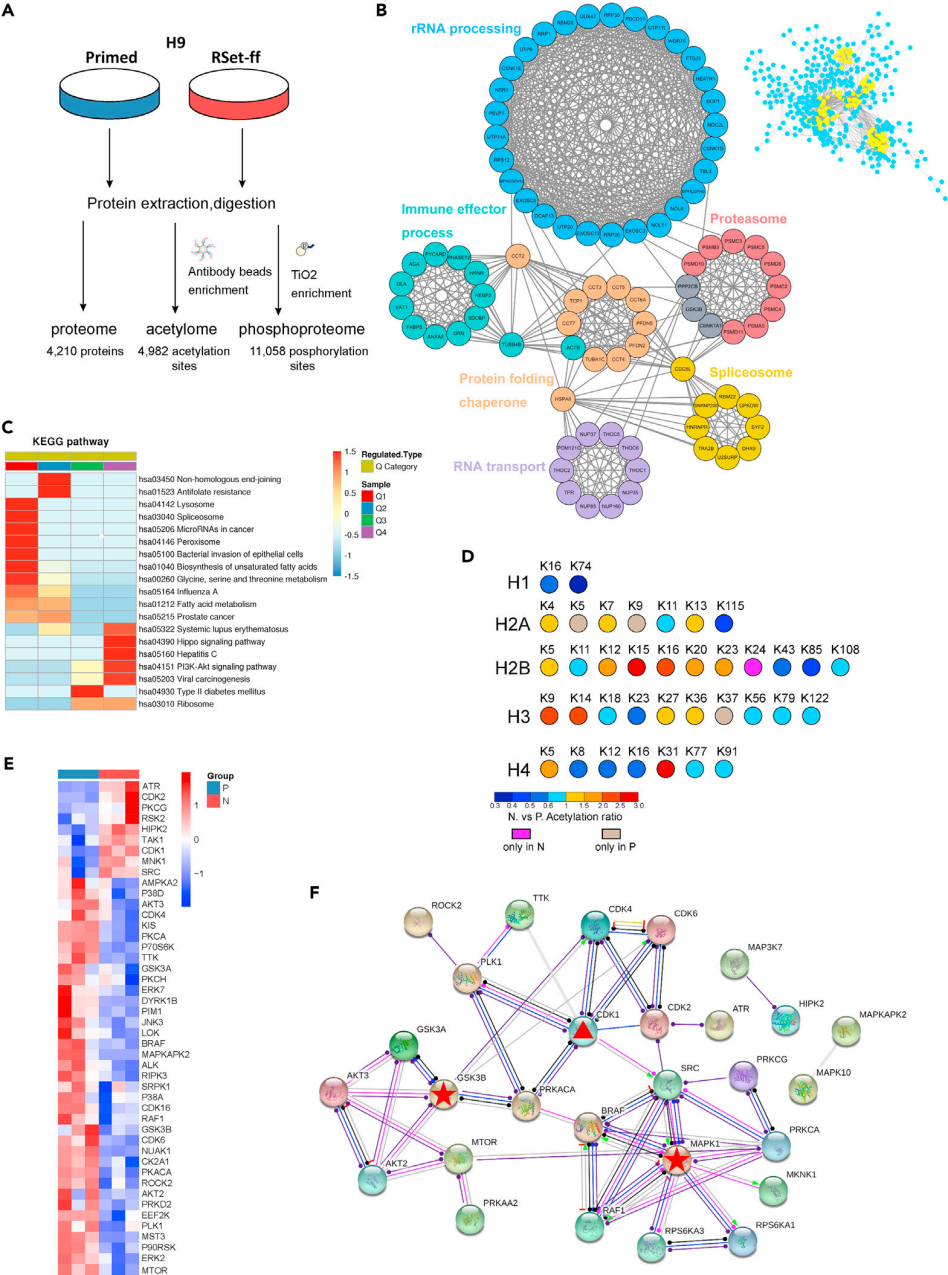
Global views on biological processes, histone acetylation and kinase activity in primed versus RSet-ff-induced naive H9 hESCs (A) Workflow of the main procedures for proteomic, acetylomic, and phosphoproteomic analyses. (B) All the differentially expressed proteins between the two pluripotency states were subjected to MCODE and PPI analysis. Each blue node represents a differential protein, and the gray lines connecting the nodes represent PPIs. 6 modules comprising highly inter-connected nodes were highlighted in yellow (higher right), sorted out, and given more details (left). (C) Differential non-histone acetylation sites were classified into four levels, Q1 through Q4 (Q1 (ratio <0.5), Q2 (0.5 < ratio <0.66), Q3 (1.5 < ratio <2), and Q4 (ratio >2)), in the order of increasing naive/Primed fold changes. The KEGG pathway enrichment analysis for the classified acetylation sites was shown. (D) A summarized map showing the naive/Primed fold changes for acetylation sites on histone subunits. N, naive; P, primed. (E) Kinase activities inferred from IKAP may shape the phosphoproteome in naive versus primed hESCs. (F) Kinase-to-kinase interaction network. The asterisks indicate kinases that are well-established in maintaining naive pluripotency, and the triangle represents a novel kinase that is centrally positioned in the network.

To gain novel insights into the differential protein translational and post-translational regulatory mechanisms in the two hESC pluripotency states, we used label-free LC-MS/MS approaches to quantify and compare the proteome, acetylome, and phosphoproteome of primed and RSet-ff naive H9 hESCs in biological triplicates. As depicted in Figure 2A, H9 cells were extracted, digested, and enriched by antibody beads or TiO_2_ for acetylome or phosphoproteome, respectively, then analyzed by LC-MS/MS. In total, we quantified 4210 proteins, 4982 acetylation sites, and 11,058 phosphosites, with equal amounts of the total protein from the two hESC states being analyzed (Tables S1, S2, and S3). Principal-component analysis (PCA) of three omics showed considerable differences between primed and naive cells that were generally kept consistent in three replicates (Figure S3A). Relative standard deviations (RSDs) of quantitative values between replicates were less than 0.2, suggestive of high reproducibility (Figure S3B).

With respect to more detailed phosphorylation and acetylation patterns, we totally identified 1004 proteins with 1 phosphosite, 519 proteins with 2 phosphosites, 316 proteins with 3 phosphosites, and 228 proteins with 4 phosphosites (Figure S3C). Corresponding acetylation data were also represented. The dominant phosphorylated amino acids were serine (85%), followed by threonine (14%), and only 0.9% of the phosphor-sites were identified as tyrosine (Figure S3D). The differential proteins and PTM sites were selected according to the following criteria: the modification site localization probability >0.75; the quantification ratio >1.5 or <0.66 (naive versus primed); the p value < 0.05.

### Multi-omics analyses reveal differences in proteome, histone acetylation, and kinase activity between primed and naïve human embryonic stem cells

Firstly, we analyzed the proteomic data and sought to identify the most differentially regulated biological processes between the two pluripotency states. We subjected all the differentially expressed proteins between the two states to an integrated Molecular Complex Detection (MCODE) analysis and the protein–protein interaction (PPI) network analysis via the STRING database. 6 modules (comprising 84 proteins) within which the protein members are highly interconnected, were identified from global PPI network (Figure 2B). One or two key members in a module mediated connections with other modules. Then, we tried to associate the majority of these modules with known gene annotation terms and physiological processes. For example, the largest module was mainly comprised of proteins participating in ribosome RNA (rRNA) processing. Additionally, a series of function annotations including spliceosome, RNA transport, immune effector process, proteasome, and protein folding chaperone were linked to other 5 modules. Of note, 3 out of these 6 modules were associated with RNA-related post-transcriptional processes, namely splicing, transport, and translation initiation, and 2 out of them were related to proteostasis, namely proteasome and protein folding chaperone. Furthermore, heatmap and cluster analyses were performed for every single module described above, showing consistent down-regulation of RNA processing-related 3 modules and up-regulation of the other 3 modules in naive pluripotency (Figure S4A). As a parallel analysis, the global connections among various KEGG pathways for all differential proteins were established (Figure S4B). Among them, three significantly enriched pathways governing translation and energy metabolism, namely RNA transport, ribosome biogenesis in eukaryotes, and glycolysis/gluconeogenesis, were chosen for further analysis, and the PPI network for each chosen pathway was shown in Figure S4C. Thus, various analyses of proteomic data consistently indicate global repression of RNA processing and translation pathways in human naive pluripotency. Together with the up-regulated proteostasis machinery, the down-regulated translational machinery is likely to give rise to a reduced total protein level and proteome size in naive hESCs.

Next, we separately analyzed the acetylome and phosphoproteome data. By using the MoMo software (http://meme-suite.org/tools/momo), the position-specific frequencies of ten flanking amino acid residues up- and downstream of the detected acetylation sites in naive hESCs were compared. Five acetyl-K motifs were identified and KY was the most conserved one (Figure S4D). Besides histone proteins, acetylation modifications have been identified in numerous non-histone proteins participating in various cellular processes (Narita et al., 2019). In this study, highly acetylated non-histone proteins in naive state were significantly enriched in pathways concerning cell proliferation. In contrast, less acetylated non-histone proteins were mainly involved in the metabolism of amino acids and fatty acids (Figures 2C and S4E). Given the crucial role of histone acetylation as a major epigenetic marker in regulating early development (Moussaieff et al., 2015; Ware et al., 2009), we summarized the quantitative acetylation data for all histones in Figure 2D. In general, the overall degree of histone acetylation in naive hESCs was similar to that in primed hESCs. The naive hESCs had an increase in H3K9Ac, an epigenetic marker representing transcriptionally active chromatin, and is involved in pluripotency maintenance (Qiao et al., 2015). The unique H2BK24Ac, as well as the prominently increased H2BK15Ac and H4K31Ac in naive hESCs may warrant special attention and further in-depth investigation.

For phosphosites, we identified 6 significantly enriched phosphor-S (pS) motifs and three phosphor-T (pT) motifs in naive hESCs (Figure S4F). Owing to the limited number of phosphor-Y (pT, phosphorylated tyrosine) sites, no common motifs can be identified. The frequency analysis for amino acids around the phosphosites revealed the preferred residues following pS11 were P12 and E14. TP is known to be by far the most common pT motif, which was also confirmed in this study. Furthermore, a machine learning algorithm, IKAP (Mischnik et al., 2016), was used to infer the upstream kinases that phosphorylate multiple downstream substrates whose phosphorylation sites and levels were revealed by the quantitative phosphoproteomics after the normalization of the phosphopeptide abundance by the protein abundance. Out of the analyzed 170 kinases, 46 differentially regulated kinases were clustered (Figure 2E). Only 9 kinases were relatively more active in naive hESCs, including CDK1, CDK2, ATR, PKCG, RSK2, HIPK2, TAK1, MNK1, and SRC. We then constructed a kinase-to-kinase interaction network for all the differentially regulated kinases, rooted on centrally positioned kinases MAPK1 and GSK3B that are known to be suppressed by 2i in almost all naive culture systems (Takashima et al., 2014; Theunissen et al., 2016) (Figure 2F). Strikingly, a quarter of the clustered kinases had a direct or indirect connection to CDK1, a kinase with potentially up-regulated activities in naive hESCs (Figures 2E and 2F).

In summary, we identified a series of differential acetylation sites on the histones and constructed the kinase-to-kinase interaction network for multiple upstream kinases that may critically shape the phosphoproteome. Remarkably, four members of the CDK family (CDK1, CDK2, CDK4, and CDK6) stood out as potential key regulators for naive pluripotency.

### CDK1 activity is crucial for maintaining naïve pluripotency

**Figure 3:**
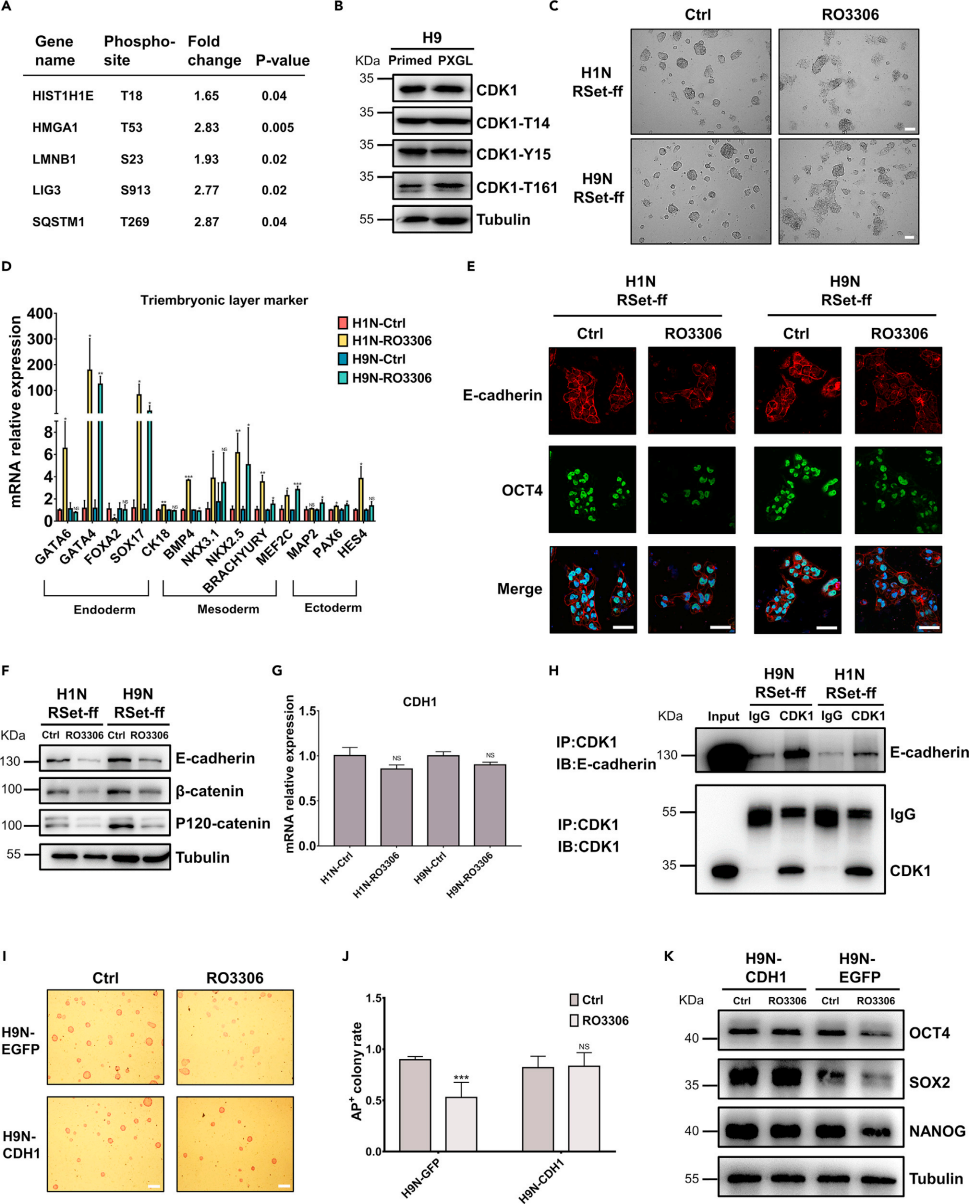
CDK1-regulated E-cadherin functionality is crucial for naive pluripotency (A) Selected CDK1 substrates with elevated phosphorylation levels at putative CDK1 phosphorylated sites identified in naive H9 hESC phosphoproteome. (B) Western blot showing CDK1 is more active in naive H9 cells cultured in PXGL medium, similar to those cultured in RSet-ff medium. (C) Light micrographs of naive (RSet-ff) H1 and H9 cells treated with vehicle (Ctrl) or 5 μM RO3306 for 24 h. Scale bars, 200 μm. (D) Inactivation of CDK1 by RO3306 (5 μM for 24 h) resulted in increased transcription of mesendoderm markers. naive H1 and H9 cells were cultured in RSet-ff medium. Data were presented as mean ± S.D. from three independent experiments. Unpaired t test was performed so that NS (not statistically significant) p > 0.05, ∗p < 0.05,∗∗p < 0.01, ∗∗∗p < 0.001. (E) Naïve H1 and H9 cells cultured in RSet-ff were treated with vehicle (Ctrl) or 5 μM RO3306 for 24 hours, immunostained for E-cadherin and OCT4, and counterstained with Hoechst 33342 for nuclei. Representative confocal micrographs were shown. Scale bars, 50 μm. (F) CDK1 inhibitor RO3306 (5 μM, 24 hours) led to decreased expression levels of E-cadherin and its binding partners. Naïve (RSet-ff) H1 and H9 cells were harvested and the whole-cell lysates were subjected to SDS-PAGE and immunoblotting, and detected by specified antibodies. The immunoblots shown were from one experiment representative of three independent experiments with similar results. (G) The mRNA levels of CDH1 (E-cadherin) in naïve (RSet-ff) H1 and H9 cells remained unchanged at conditions described in (F). Data were presented as mean ±S.D. from three independent experiments. Unpaired t test was performed so that NS (not statistically significant) p>0.05. (H) CDK1 interacted with E-cadherin in naïve (RSet-ff) hESCs. Naive cell lysates were immunoprecipitated (IPed) with anti-CDK1 antibody, and the immune complexes were analyzed by immunoblotting (IB) using anti-CDH1 antibody (upper) or anti-CDK1 antibody (lower). (I–K) Stable naïve (RSet-ff) H9 cell lines overexpressing EGFP or E-cadherin were generated by the lentiviral system, and named H9N-EGFP and H9N-CDH1, respectively. They were treated with DMSO (Ctrl) or 5 μM RO3306 for 24 hours. Light micrographs of alkaline phosphatase positive (AP+) colonies (I) and their statistical data (J) were shown. Scale bars, 200 μm. The data were presented as mean±S.D. of three independent experiments. (∗∗∗p <0.001). Unpaired t test was performed so that NS p>0.05, ∗∗∗p <0.001. The protein levels of key pluripotency factors were examined by immunoblotting (K).

Given the above analyses, we next focused on the CDK family in the two pluripotency states. Our pilot experiments using specific small-molecule inhibitors showed that CDK1 rather than other CDK family members is critical for human naive pluripotency. CDK1 is a unique mitosis cyclin-dependent kinase (M-CDK) indispensable for early embryonic development (Santamaria et al., 2007). It has been reported that CDK1 is required for maintaining human primed pluripotency (Neganova et al., 2014), yet its potential role in naive pluripotency has not been documented. As shown in the phosphoproteome data, the phosphorylation levels at known CDK1-phosphorylated sites in some selected CDK1 substrates were higher in RSet-ff naive hESCs (Figure 3A), implicating a higher CDK1 catalytic activity in naive pluripotency. This was consistent with the immunoblotting data showing that the level of total CDK1 protein was increased while that of the inhibitory phosphorylation on T14 and Y15 was reduced in RSet-ff naive hESCs (Figure S5A). Under the PXGL naive induction conditions, the level of inhibitory phosphorylation on Y15 was reduced and that of activating phosphorylation on T161 was elevated in naive H9 cells as opposed to their primed counterparts (Figure 3B), confirming enhanced CDK1 activities in naive pluripotency. Strikingly, treating RSet-ff naive hESCs but not their primed counterparts with the highly specific CDK1 inhibitor RO3306 (Jorda et al., 2018), which had no discernable effects on cell viability at a concentration of 5 μM (Figure S5B), converted their colony morphology from domes to flattened sheets (Figures 3C and S5C). The pluripotency markers (NANOG, SOX2, and OCT4) and naive markers (STELLA, KLF4, and DNMT3L) were both decreased (Figures S5D–S5F). Meanwhile, examination of germ layer markers revealed the differentiation of RO3306-treated naive hESCs toward mesendoderm (Figure 3D). Thus, CDK1 is more active in naive hESCs and is essential for maintaining their characteristics.

To investigate if the impairment of naive pluripotency by CDK1 inhibitor was mediated by the traditional role of CDK1 in G2/M progression, as reported for primed hESCs (Neganova et al., 2014), cell cycle analyses were conducted for RSet-ff-induced naive H1 and H9 cells with or without RO3306 treatment. At 1 day after RO3306 treatment, a higher proportion of naive H1 cells were arrested at the G2/M phase while naive H9 cells were mainly S/G2 arrested (Figure S5G), indicating differential roles of CDK1 in cell cycle regulation between naive H1 and H9 cells. Therefore, it seems unlikely that the cell cycle regulation by CDK1 plays a conservative and major role in maintaining human naive pluripotency.

The above morphological transition by CDK1 inhibitor treatment drew our attention to the cell–cell adhesion complexes. E-cadherin, a calcium-dependent cell adhesion protein, has a pivotal role in forming the compact colonies of mESCs and in early embryogenesis (Li et al., 2012; Pieters and van Roy, 2014). Remarkably, upon RO3306 treatment, the continuously and cortically distributed E-cadherin disappeared only in naive (Figures 3E and S6A) but not primed hESCs (Figure S6B). RO3306 significantly reduced E-cadherin protein levels (Figure 3F) but not its mRNA levels (Figure 3G) in naive hESCs but not in primed hESCs (Figure S6C), and it also reduced the protein levels of β-catenin and p120-catenin to a similar extent (Figure 3F), indicating that CDK1 may directly regulate E-cadherin/β-catenin/p120-catenin protein complex via its kinase activity in naive hESCs. Importantly, either CDK1 knockdown or E-cadherin (CDH1) knockdown impaired the establishment of naive pluripotency, and the trends of change in naive markers were similar between the two knockdown groups (Figures S6F and S6G). Co-IP experiments demonstrated that there was physical interaction between E-cadherin and CDK1 proteins in naive hESCs (Figures 3H and S6D). Remarkably, overexpressing E-cadherin (Figure S6E) can rescue RO3306-induced reduction of AP + colony formation (Figures 3I and 3J) and reduction of pluripotency markers (Figure 3K), and thereby maintaining naive pluripotency. Taken together, we reveal here that CDK1 critically maintains human naive pluripotency by sustaining the protein level and cortical localization of E-cadherin.

### Integrative multi-omics analyses reveal RNA processing and translation as the most differentially regulated processes between primed and naïve human embryonic stem cells

**Figure 4:**
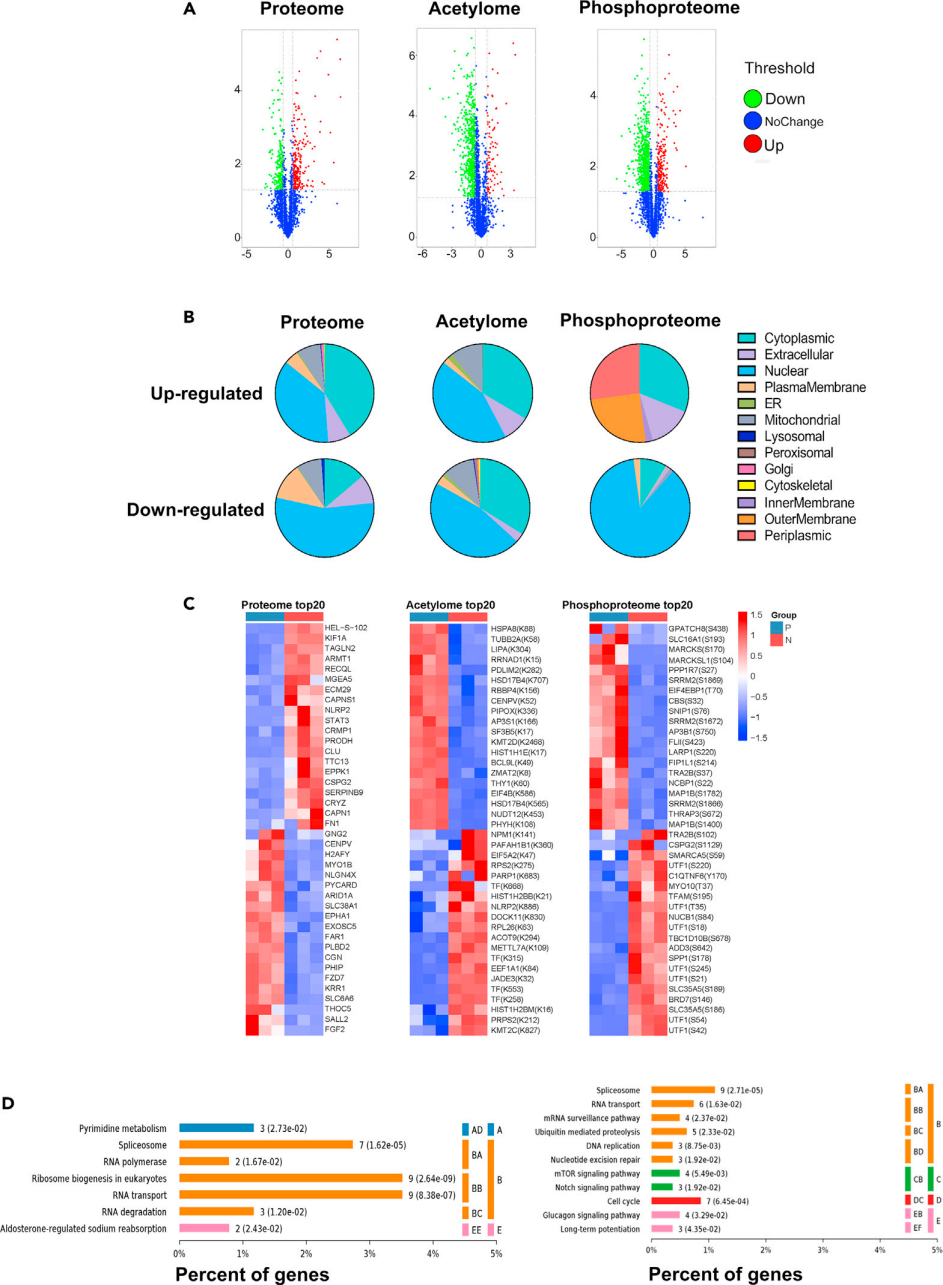
Integrative multi-omics analyses implicating RNA Processing and Translation as the most differentially regulated processes between primed and naive hESCs (A) Volcano plot showing global distribution of significantly differential proteins and PTM sites. The vertical axis represents the p value (-log10) for differences between naive (RSet-ff) and primed H9 hESCs, and the horizontal dimension is the fold change (log2) between the two groups. Red dots: p value < 0.05, naive/Primed fold change >1.5; green dots: p value < 0.05, naive/Primed fold change <0.66. (B) Subcellular distribution for up-regulated and down-regulated proteins, acetylation sites, and phosphosites in naive (RSet-ff) H9 hESCs. (C) Clustering of the top 20 up- and down-regulated hits in naive (RSet-ff) versus primed H9 hESCs from three omics analyses. (D) KEGG pathway enrichment analysis for down-regulated proteins (left) and down-regulated phosphosites (right) in naive (RSet-ff) versus primed H9 hESCs. Functional classification of pathways: A-Metabolism: AD-Nucleotide metabolism; B-Genetic information processing: BA-Transcription; BB-Translation; BC-Folding, sorting and degradation; C-Environmental information processing: CB-Signal transduction; D-Cellular processes: DC-Cell growth and death; E-Organismal systems: EB-Endocrine system; EE-Excretory system; EF-Nervous system.

After separated analyses for each omics, as shown above, we next integrated the multi-omics data to find out the combined major differences between primed and naive pluripotency. Firstly, as shown in the Venn diagram, at the global level, there was little overlapping among the differential hits from the three omics, indicating little direct correlation between PTM dynamics and protein abundance (Figure S7A). Strikingly, in naive hESCs there were much more identified sites with down-regulated acetylation and phosphorylation levels than those with up-regulated ones, despite the down-regulated total protein numbers were similar to up-regulated ones (Figure 4A), indicating globally suppressed PTM levels in naive hESCs as opposed to primed hESCs. Furthermore, Subcellular location annotation information (Yu et al., 2006) showed that almost three-quarters of total differentially changed proteins and PTM sites were localized at the nucleus and cytosol. The proportion of cytoplasmic localization was up to 41% in up-regulated proteins, while over half of the down-regulated proteins were nuclear localized. For differential acetylation sites, there was a significant increase in the proportion of mitochondrial localization, reaching 10% of the total. In comparison, nearly 63% of the differential phosphosites were localized at the nucleus and, more interestingly, almost all of them were down-regulated in naive compared to primed hESCs (Figures 4B and S7B).

**Figure 5:**
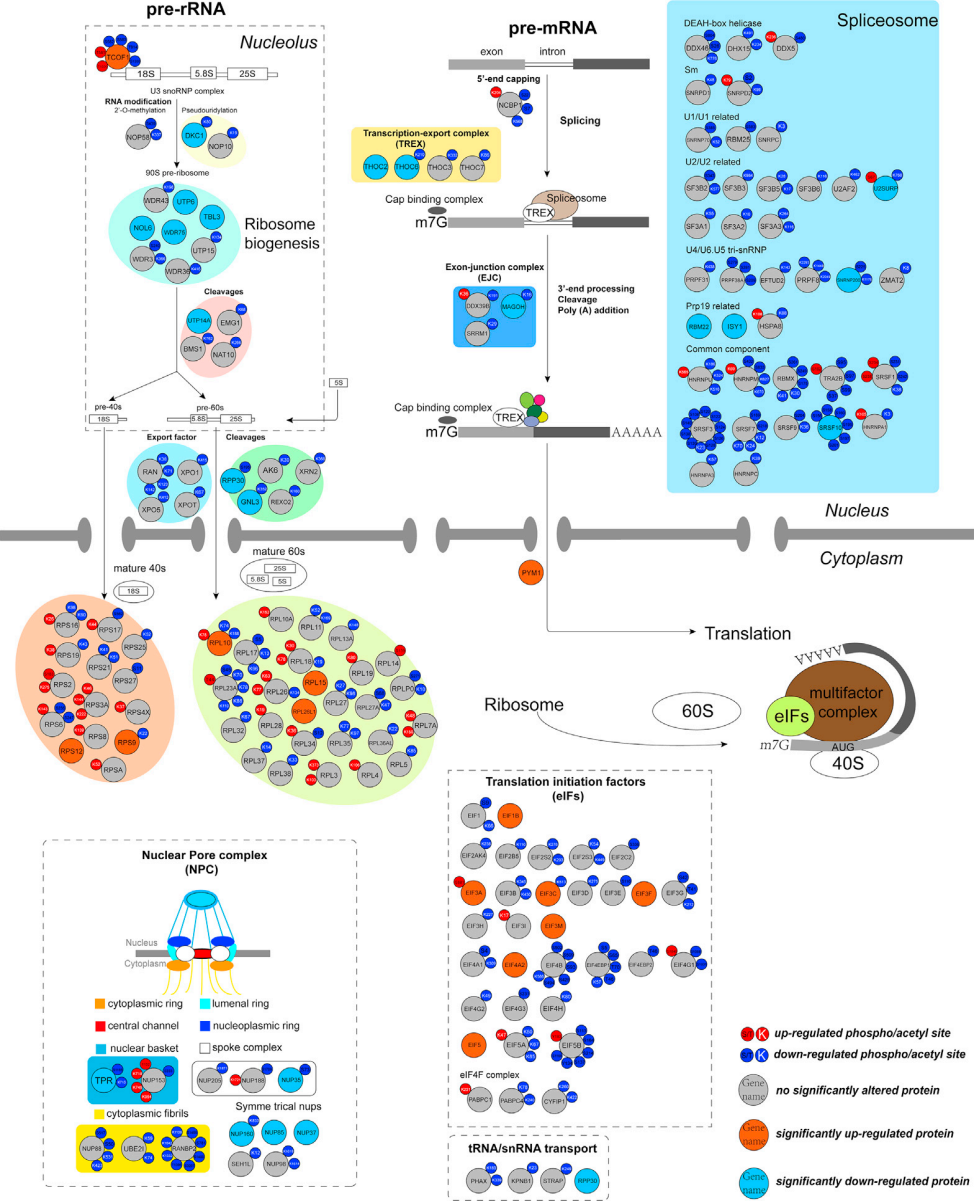
Schematic summary of multi-omics data showing differential proteins, phosphosites, and acetylation sites associated with RNA processing and translation in naive (RSet-ff) versus primed H9 hESCs Large circles represent individual proteins that are compared between naive and primed H9 hESCs. Light red: significantly up-regulated proteins in naive H9 hESCs; Light blue: significantly down-regulated proteins in H9 hESCs; Gray: no significantly altered protein levels between naive and primed H9 hESCs. Small circles surrounding each large circle represent individual post-translational modification sites that are compared between naive and primed H9 hESCs. Red: up-regulated PTMs; Blue: down-regulated PTMs. The labels in small circles denote specific residues. Black labels: phosphorylated residues. White labels: acetylated residues.

Furthermore, when clustering the top 20 up- and down-regulated proteins/PTM sites between primed and naive hESCs from all the three omics together (Figure 4C), we found a considerable number of down-regulated proteins/PTM sites were involved in RNA processing and translation, such as EXOSC5, THOC5, eIF4B (K586), SF3B5 (K17), LARP1 (S220), and eIF4EBP1 (T70). When non-biasedly taking all differentially expressed proteins/PTM sites into analysis, we found that both down-regulated proteins and phosphosites in naive hESCs were significantly enriched for RNA processing and translation-related pathways including RNA transport, spliceosome, RNA degradation, ribosome biogenesis in eukaryotes, RNA polymerase and mRNA surveillance pathway (Figure 4D), while up-regulated proteins in naive hESCs were most enriched for metabolic pathways (Figure S7C) and up-regulated phosphosites were enriched for lysine degradation and spliceosome (Figure S7D). With respect to acetylation, KEGG analysis revealed 4 RNA-related pathways with up-regulated acetylation sites that included ribosome, spliceosome, mRNA surveillance pathway, and RNA degradation, while 4 RNA-related pathways with down-regulated acetylation sites that included RNA transport, spliceosome, ribosome, and ribosome biogenesis in eukaryotes (Figures S7E and S7F). Taken together, by combining quantitative proteomics, phosphoproteomics, and acetylproteomics analyses, we revealed RNA processing and translation as the most differentially regulated processes between naive and primed hESCs, and mapped out the key differential proteins/PTM sites (Figure 5, Table S4).

### Naive human embryonic stem cells with lower mammalian target of rapamycin complex 1/4E-binding protein1 activities rely more on the eukaryotic initiation factor 4E-independent translation initiation pathway

**Figure 6:**
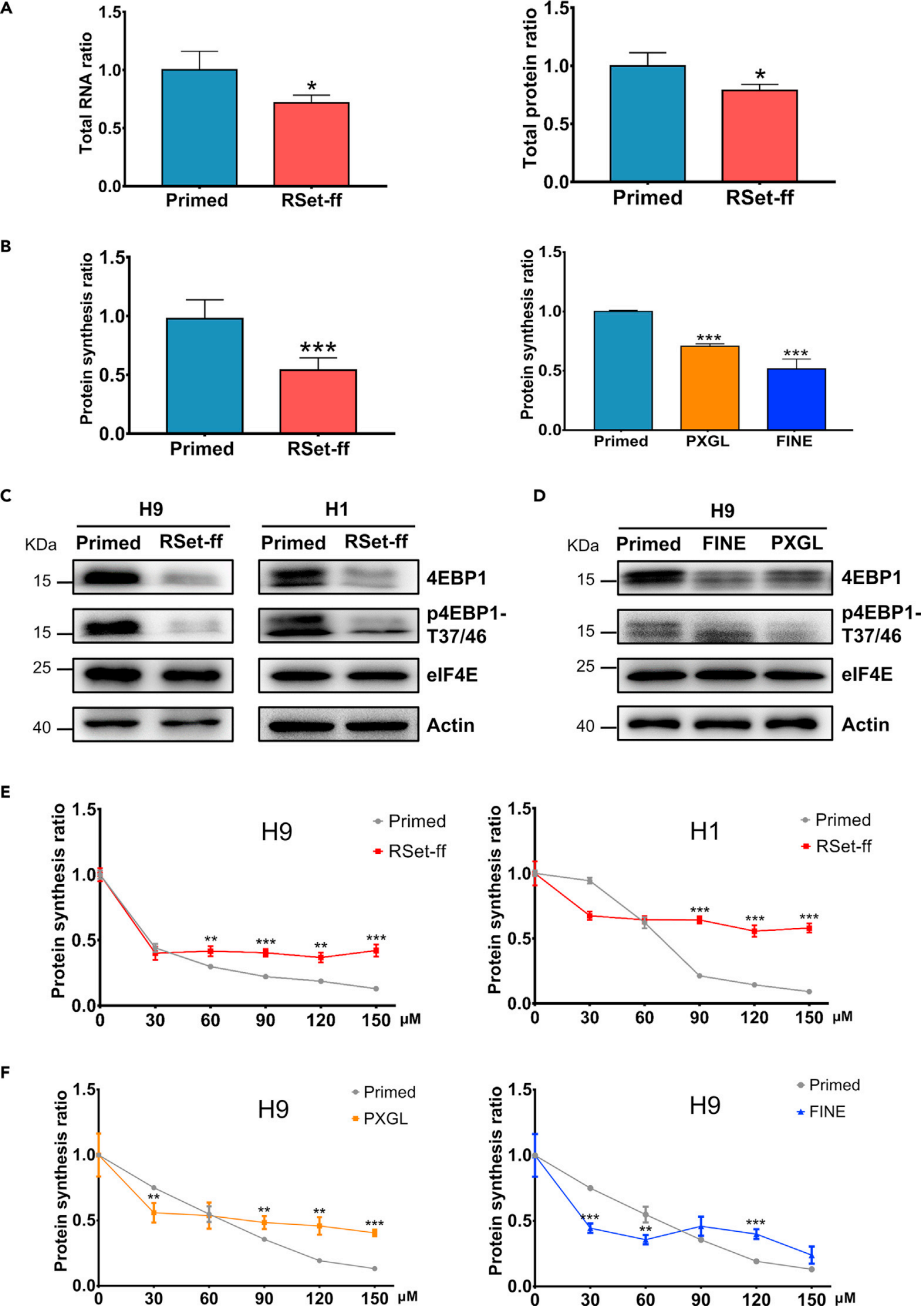
Naïve hESCs with lower mTORC1/4EBP1 activities are more tolerant to eIF4E blockade (A) Primed and naive (RSet-ff) H9 cells were harvested and counted. Total RNAs (left) and total proteins (right) from equal number of primed and naive cells were extracted and quantified. The data were presented as mean ± S.D. from three independent experiments, with the values of primed cells being set at 1. Unpaired t test was performed so that ∗p < 0.05. (B) Primed and naive (RSet-ff, left; PXGL and FINE, right) H9 cells were incubated for 30 min with 20 μM of OPP, followed by cell fixation with 1% paraformaldehyde and fluorescent labeling with Alexa Fluor™ 488 in a click reaction, and finally fluorescence imaging and quantification using the Operetta CLS High-Content Analysis System. The normalized mean Alexa Fluor™ 488 fluorescence intensities indicative of incorporated nascent polypeptide chains were presented as mean ± S.D. from three independent experiments, with the values of primed cells being set at 1. Unpaired t test was performed so that ∗∗∗p < 0.001 (C and D) Primed and naive (RSet-ff, C; FINE and PXGL, (D) hESCs were harvested and the whole-cell lysates were subjected to SDS-PAGE and immunoblotting, and detected by specified antibodies. The immunoblots shown were from one experiment representative of three independent experiments with similar results. (E) Primed and naive (RSet-ff) H9 (left) or H1 (right) cells were treated with varying concentrations of 4E1RCat for 2.5 h, followed by nascent protein translation rate determination as described in (B). The normalized mean Alexa Fluor™ 488 fluorescence intensity indicative of incorporated nascent polypeptide chains was taken as the global nascent protein synthesis rate, and the ratio of global nascent protein synthesis rate with 4E1RCat treatment over that with vehicle treatment was presented as mean ± S.D. of triplicate measurements from one experiment representative of three independent experiments with similar results. Unpaired t test was performed so that ∗∗p < 0.01, ∗∗∗p < 0.001 (F) Naïve H9 cultured in other two media (PXGL, left; FINE, right) were treated and analyzed as described in (E).

Given the globally repressed RNA processing and translation pathways identified in naive hESCs, we directly quantified and compared the total RNA and protein levels in the two pluripotency states. The total RNA and protein levels in primed hESCs were 1.4-fold and 1.3-fold higher than those in equal number of their RSet-ff naive counterparts, respectively (Figure 6A). To assess the rate of global protein synthesis, cells were incubated for 30 min with O-propargyl-puromycin (OPP), a puromycin analog that generates covalent conjugates with nascent polypeptide chains, followed by cell fixation and fluorescent labeling with Alexa Fluor™ 488 in a click reaction, and finally fluorescence imaging and quantification using the Operetta CLS High-Content Analysis System. The Alexa Fluor™ 488 fluorescence intensity indicative of nascent protein translation rate in primed hESCs was 1.8-fold higher than that in RSet-ff naive cells (Figure 6B left and S8A), an observation directly supporting the major conclusion from the above multi-omics analyses. Meanwhile, we also confirmed that the translation efficiency in naive H9 cells derived in PXGL or FINE medium was equivalent to only 70% or 50% of that of the primed H9 cells, respectively (Figure 6B right). In addition, consistent with the up-regulated proteasome activity identified from the above MCODE analysis (Figures 2B and S4A), naive hESCs exhibited higher degree of global ubiquitination (Figure S8B). However, when treated with the translational elongation inhibitor cycloheximide (CHX) to block the synthesis of new proteins, the revealed overall protein degradation rate in naive hESCs was only slightly higher than that in their primed counterparts (Figure S8C). Thus, we speculate that the greatly strengthened ubiquitin/proteasome system in naive hESCs may form a part of the protein quality control system to specifically and efficiently remove those deficient, impaired, or redundant proteins to maintain a compact naive proteome.

We next examined the mammalian target of rapamycin complex 1 (mTORC1), a serine/threonine kinase and master regulator positively promotes biosynthesis of proteins by adjusting ribosome biogenesis, mRNA biogenesis, and cap-dependent translation (Laplante and Sabatini, 2009). Both the total and phosphorylated mTOR protein levels in RSet-ff naive hESCs were much lower than those in primed hESCs (Figure S8D), confirming the predicted kinase activities shown in Figure 2E. In PXGL- or FINE-induced naive hESCs, however, the total and phosphorylated mTOR protein levels were equivalent to or only slightly lower than those in their primed counterparts (Figure S8D). Besides, naive hESCs derived from three conditions all had a higher level of total PRAS40 protein, the well-established mTORC1 suppressor, and a lower level of phosphorylated PRAS40 (except for FINE naive hESCs) that can be dissociated from mTORC1 and relieve its inhibition on mTORC1 (Figure S8D). Unexpectedly, however, AKT serine/threonine-protein kinase, the presumed positive upstream regulator of mTORC1, was more activated in naive hESCs (Figure S8E), indicating a decoupling of AKT and mTORC1 signaling in human naive pluripotency. Although the total protein level of insulin-like growth factor 1 receptor (IGF1R), the putative upstream activator of PI3K/AKT signaling, was higher in naive hESCs, the level of phosphorylated IGF1R in two pluripotency states was similar (Figure S8E). Thus, the upstream regulator for elevated AKT activity in naive pluripotency remains elusive, and it remains to be seen if elevated CDK1 in naive hESCs may promote the PI3K-AKT pathway via PDK1 as previously reported (Wang et al., 2017).

To assess the impact of mTORC1 on protein synthesis in two pluripotency states, we examined one of the mTORC1 substrates, the eukaryotic initiation factor 4E-binding protein 1 (4EBP1), which plays a key role in controlling translation initiation. When 4EBP1 is phosphorylated by active mTORC1, it can be disassociated from eIF4E which can then critically mediate cap-dependent translation (Richter and Sonenberg, 2005). Remarkably, compared with the primed hESCs, both total and phosphorylated 4EBP1 protein contents were significantly reduced in naive hESCs induced by three systems (Figures 6C and 6D) while the total eIF4E protein levels were similar in the two pluripotency states (Figures 6C and 6D). Given the evidence that in mitotic cells CDK1 can hyperphosphorylate 4EBP1 in the absence of mTORC1 activity (Shuda et al., 2015), elevated CDK1 in naive hESCs may directly phosphorylate and regulate 4EBP1 and other translation machinery. Indeed, our bioinformatics analysis indicated that some key translational regulators such as eIF2 subunits and EIF4ENIF1 are probable substrates for CDK1 (Table S5). Thus, the eIF4E-dependent translation initiation processes in naive hESCs appeared to rely much lesser on mTORC1/4EBP1 than primed hESCs.

Accordingly, when the two pluripotency state hESCs were treated with XL388, a specific mTORC1 inhibitor that had no discernable effects on cell viability at low doses (Figure S8F), there was a more prominent reduction of translation efficiency in primed hESCs compared to RSet-ff naive hESCs (Figure S8G). Taken all together, we propose that primed hESCs mainly rely on the eIF4E-mediated cap-dependent pathway for protein translation while naive hESCs with higher CDK1 (Figure 3) and lower mTORC1 activities (Figure 6) are more tolerant to eIF4E blockade.

### Identification of eukaryotic initiation factor 4E-independent and eukaryotic initiation factor 4A2-dependent translation pathways in naïve human embryonic stem cells

To quantify the proportion of eIF4E-dependent translation in two pluripotency states, we treated cells with 4E1RCat, a compound that can specifically block the formation of the eIF4F complex and hence eIF4E-mediated translation (Cencic et al., 2011). Although there was a dose-dependent inhibition of protein synthesis by up to 90% in primed hESCs treated with 4E1RCat, approximately 40%, and 60% protein synthesis was retained in RSet-ff naive H9 and H1 cells, respectively, even at the maximal dose of 4E1RCat (Figures 6E and S9A). Similarly, maximum inhibition of protein synthesis by 4E1RCat was approximately 50%–60% for both PXGL- and FINE-induced naive H9 cells compared to 90% inhibition for their primed counterparts (Figure 6F). The pictures containing fluorescence intensity indicative of nascent protein translation rate in different concentrations of 4E1RCat are shown in Figure S9B. These results indicated that under 4E1RCat-treated conditions, primed hESCs predominantly rely on eIF4E-dependent translation while a considerable proportion of translation in naive hESCs is via eIF4E-independent pathways. However, it is unclear if the eIF4E-independent translation is a compensatory mechanism under inhibitor-treated stress conditions or an intrinsic process under physiological conditions.

**Figure 7:**
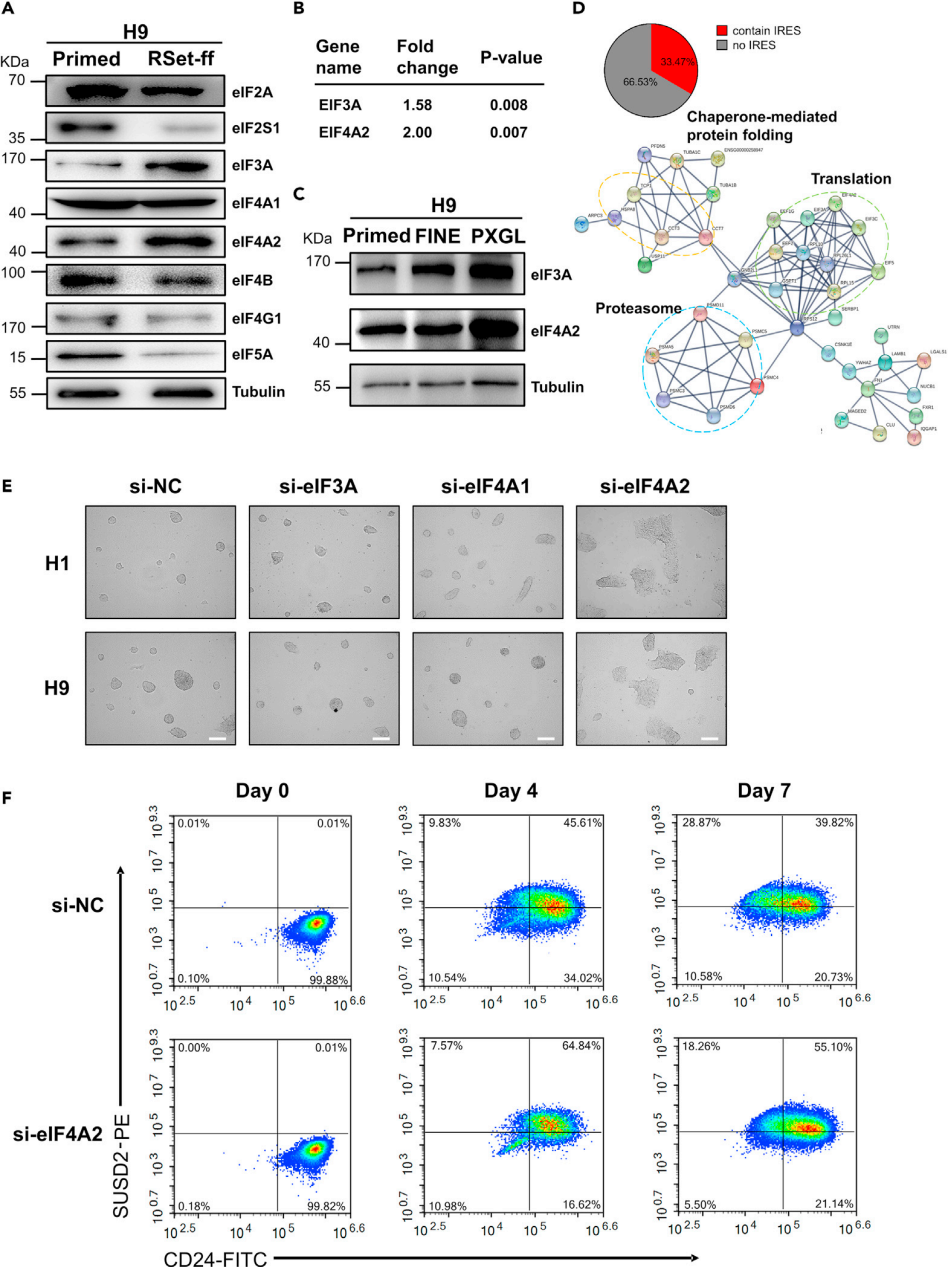
eIF4A2 is essential for inducing human naive pluripotency. (A and B) Primed and naive (RSet-ff) H9 cells were harvested and the whole-cell lysates were subjected to SDS-PAGE and immunoblotting with specified antibodies (A). The immunoblots shown were from one experiment representative of three independent experiments with similar results. The relative band intensity of eIF3A and eIF4A2 in two pluripotency states was consistent with that of the quantitative data from proteomics shown on the right (B). (C) Whole-cell lysates of primed and naive (FINE and PXGL) H9 cells were subjected to SDS-PAGE and immunoblotting with specified antibodies. The immunoblots shown were from one experiment representative of three independent experiments with similar results. (D) Pie chart (upper) showing the percentage of the up-regulated proteins in naive (RSet-ff) H9 hESCs that contain typical IRES structures in their mRNAs. The known interactions among these proteins were revealed by STRING analysis (lower). Three enriched pathways were circled. (E) Morphological changes in primed H9 hESCs transfected with scramble siRNA (si-NC) or specified siRNAs and induced to naive pluripotency in RSet-ff media for 48 h. Scale bars, 200 μm. (F) Flow cytometry analysis of SUSD2 and CD24 expression during the induction of scramble siRNA (si-NC) or eIF4A2 siRNA (si-eIF4A2) transfected primed H9 into naive pluripotency in PXGL culture system.

To gain some insights into the eIF4E-independent translation mechanism in naive pluripotency, we quantitatively compared the protein levels of multiple eukaryotic initiation factors in two pluripotency states by immunoblotting. The total protein levels of eIF4G1, eIF4B, eIF2A, and eIF5A in naive hESCs were significantly lower (Figure 7A), indicating a global suppression of eIF4E-dependent translation that is consistent with a reduction of global translation rate and total protein level as shown above (Figures 6A and 6B). On the contrary, consistent with the proteomic data, under the RSet-ff induction conditions, we demonstrated higher levels of eIF3A in naive H9 and H1 cells, and a higher level of eIF4A2 in naive H9 cells (Figures 7A, 7B, and S10A). The same increased pattern for eIF3A and eIF4A2 was held in PXGL naive H9 cells. However, FINE naive H9 cells showed a higher level of eIF3A but the same level of eIF4A2 compared to their primed counterparts (Figure 7C). Given the critical roles of eIF3 and eIF4A in regulating IRES-mediated cap-independent translation (Cate, 2017; Komar and Hatzoglou, 2011; Tsai et al., 2014), we asked to what extent the eIF4E-independent translation was mediated by IRESs. By integrating data from a previous study (Weingarten-Gabbay et al., 2016), we found that about 33% of the up-regulated proteins in naive hESCs contained typical IRES structures in their mRNAs that may translate in a cap-independent manner (Table S6). PPI analysis revealed a significant enrichment of three biological processes (translation, chaperone-mediated protein folding, and proteasome) among these IRES-translated proteins (STRING PPI with the highest confidence factor of 0.9) (Figure 7D).

As naive hESCs rely substantially on the eIF4E-independent pathway for translation (Figures 6E and 6F), we wondered if the eIFs that are involved in eIF4E-independent translation and up-regulated in naive hESCs are also crucial for naive pluripotency. To this end, we knocked down eIF3A or eIF4A (including eIF4A1 and eIF4A2) with siRNAs in primed H9 cells (Figures S10B and S10C) and induced them under the RSet-ff conditions for another 48 h. Strikingly, the reduction in eIF4A2 protein levels largely prevented the formation of dome-shaped naive H9 colonies (Figure 7E) and significantly reduced the levels of naive marker transcripts in converted hESCs (Figure S10D). In contrast, knockdown of eIF3A, or even knockdown of eIF4A1 that has higher than 90% protein sequence similarity to eIF4A2, did not inhibit the formation of naive morphology (Figure 7E). On day 7 of the PXGL induction conditions, the percentage of formed SUSD2+CD24-naive cells dropped significantly from 29% in the si-NC control group to 18% in the si-eIF4A2 group (Figure 7F). We, therefore, concluded that eIF4A2 is essential for inducing human naive pluripotency under both RSet-ff and PXGL conditions.

### Bivalent metabolism drives both eukaryotic initiation factor 4E-dependent and eukaryotic initiation factor 4E-independent translation in naïve human embryonic stem cells while eukaryotic initiation factor 4E-dependent translation in primed human embryonic stem cells relies exclusively on glycolysis

As mRNA translation is one of the most energetically demanding processes in the cell (Rolfe and Brown, 1997), and primed hESCs utilize aerobic glycolysis while naive hESCs adopt bivalent metabolism (both glycolysis and OXPHOS) (Leibovitch and Topisirovic, 2018; Tsogtbaatar et al., 2020), we wondered if there is any association between metabolism modes and translation pathways. GSK2837808A (GSKA), a selective lactate dehydrogenase A (LDHA) inhibitor (Hu et al., 2018), and lonidamine (LONI), a selective hexokinase inhibitor (Guo et al., 2016), were used to selectively block glycolysis and OXPHOS, respectively. To minimize indirect or nonspecific effects that could result from prolonged inhibition, we treated cells for only 4 h. Disturbing energy metabolism dramatically decreased protein synthesis in both pluripotency states. GSKA dose-dependently reduced translation in primed hESCs, reaching about 90% reduction at the dose of 900 μM, whereas LONI had a much lesser inhibitory effect on translation (Figure S10E), suggesting that glycolysis is the predominant metabolic mode to supply energy for translation in primed hESCs. In contrast, GSKA or LONI alone had a similar dose-dependent inhibitory effect on translation in naive hESCs (Figure S10E), indicating a roughly equivalent contribution of glycolysis and OXPHOS to translational efficiency and protein synthesis in naive pluripotency where neither glycolysis nor OXPHOS alone could suffice. It is noteworthy that GSKA caused a much faster translation reduction in naive hESCs than in primed hESCs, supporting the assumption that glycolysis-regulated translation accounts for almost all translation activities in primed hESCs but only a portion of translation activities in naive hESCs. Finally, to find out if the different metabolic mode-mediated translation in naive hESCs has a preference in eIF4E usage, we combined varying doses of the two metabolic inhibitors with a fixed dose of 4E1RCat, respectively. At low doses, GSKA or LONI did not further reduce translation activities on top of those reduced by 4E1RCat (Figure S10F), suggesting that both compounds preferentially inhibit eIF4E-dependent translation. Higher concentrations of both compounds (GSKA >100 μM, LONI >200 μM) further brought the translation activities down to levels below those achieved by 4E1RCat treatment, indicating a gradual inhibition of eIF4E-independent translation (Figure S10F). We estimate that the eIF4E-independent translation accounted for at least 30% of the total translation activities in naive hESCs. Collectively, primed hESCs rely predominantly on glycolysis for eIF4E-dependent translation, while the bivalent metabolism supports both eIF4E-dependent and eIF4E-independent translation in naive hESCs and thereby conferring them with higher capabilities of survival and propagation.

## Discussion

The transition between different states of pluripotency renders a convenient and versatile *in vitro* model for understanding early human pre-implantation development, the timing and molecular basis of lineage restriction, and signal transduction in ICM cells, which will lead to improvements in hESC culture that can generate lineage-committed cells of therapeutic relevance. Our multi-omics analyses reveal here that RNA processing and protein translation are the most differentially regulated processes between naive and primed hESCs, and global RNA processing and translation are both down-regulated in naive hESCs. On one hand, our finding is consistent with the notion that during the early stages of stem cell state transition, translational control plays a dominant role (Corsini et al., 2018; Saba et al., 2021; Sampath et al., 2008; Tahmasebi et al., 2018; Wong et al., 2016). On the other hand, however, it apparently contradicts the observations in this and previous studies (Gafni et al., 2013; Ware et al., 2014) showing that naive hESCs divide more rapidly than primed hESCs. Indeed, from a broader view, the low mRNA translation rates and restricted protein synthesis in various stem cells are not compatible with their active self-renewal and propagation, and the apparent disconnection between protein translation rate and cell cycling (proliferation) rate remains an intriguing but puzzling question in the stem cell field (Blanco et al., 2016; Tahmasebi et al., 2018).

Recently, Atlasi et al. (Atlasi et al., 2020) took multiple genome-wide approaches to acquire a detailed and dynamic view of the mRNA transcription, mRNA translation, and protein abundance during the transition between 2i/LIF (2iL) ground-state (herein defined as naive mESCs) and serum/LIF (SL) (defined as primed mESCs) mESCs. They found general increases in polysome-bound mRNAs and hence translation efficiency in naive mESCs as opposed to primed mESCs, but, as a result of “translational buffering,” such increases did not lead to a concomitant elevation of global protein level. Instead, the majority of changes in the proteome were accomplished by transcriptional regulation during naive to primed mESC transition, and only a set of genes underwent specific regulation at the translational level. Thus, it was concluded that transcriptional control represents the main regulatory mode among different states of mESCs whereby specific translational and post-translational control make a lesser contribution to regulating the final protein abundance. The mechanism for “translational buffering” that may underlie the disconnection between global translation efficiency and protein abundance, and more importantly, the mechanism that coordinates protein translation rate and cell cycling rate, await further elucidation.

We showed in this study that the overall transcriptional and translational levels in naive hESCs are both lower than those in their primed counterparts. It should be noted that the hESC populations and particularly the primed hESCs are highly heterogeneous and therefore biological triplicates were set for each treatment in multi-omics analyses and most validation assays. Similar to the above-mentioned paradoxical observations, naive hESCs exhibit faster proliferation rate and shorter cycling time. Our current work offers several new clues to the paradox: firstly, as the nascent protein translation rate in naive hESCs is significantly lower than that in primed hESCs, and the overall protein degradation rate is similar between the two states, the total protein level in a naive hESC is significantly lower than that in a primed hESC. Thus, the more compact proteomes in naive hESCs are expected to require lower protein synthesis rates to maintain comparable cell division rates. Also, it was proposed that a low abundance of proteins would allow stem cells to quickly “erase” the old proteome when they receive signals to differentiate (Saba et al., 2021). Secondly, the threshold for total protein level that suffices for naive hESC division appears to be lower than that for primed hESC division, and CDK1 may be a key player to coordinate metabolism mode, translation initiation, and cell cycle progression for rapid cycling of naive hESCs. Besides its well-established role as an M-CDK that is required for G2/M progression in primed hESCs (Neganova et al., 2014), CDK1 ablation by a specific inhibitor RO3306 caused a significant arrest at the S phase, implicating its unexpected role in facilitating S to G2/M progression in naive hESCs. The Unstable Accumulating Activator models for cellular size control propose an activator that accumulates in a size-dependent manner and triggers cell cycle progression once it has reached a certain threshold, and a strong candidate for the accumulating activator in G2 is Cdc25, the phosphatase that dephosphorylates and activates CDK1 at the G2/M transition (Rhind, 2018). Clearly, the underlying mechanisms for CDK1 promoting naive hESC cycling and propagation warrant further in-depth investigation.

Thirdly, we showed that during naïve-to-primed pluripotency transition, bivalent-to-glycolytic metabolism switch is associated with a decrease in eIF4E-independent translation and an increase in and predominant reliance on eIF4E-dependent translation, and as a result, a proteome with an increased size and prolonged cycling time. Primed hESCs are known to adopt glycolysis despite sufficient oxygen availability to support OXPHOS, a phenomenon known as aerobic glycolysis, or the Warburg effect, and such incomplete oxidation of glucose enables the conservation of carbon biomass required for the biosynthesis of cellular components needed for cell proliferation and division (Tsogtbaatar et al., 2020). We reveal here that glycolysis-mediated translation initiation in primed hESCs is predominantly eIF4E-dependent. In contrast, under stressed conditions, naive hESCs utilizing bivalent metabolism can initiate translation via both eIF4E-dependent and eIF4A2-dependent/eIF4E-independent pathways. eIF4E-independent translation including IRES-mediated translation is usually initiated when cells are at early embryonic stages or exposed to certain stresses such as hypoxia, nutrition deficiency (Yang and Wang, 2019). Thus, it is likely that selective mRNAs in naive hESCs are translated via eIF4A2-dependent and/or IRES-mediated pathway with higher efficiency, and such naive proteome may confer naive hESCs with a faster propagation/cycling capability to self-renew and a higher anti-apoptotic potency to tolerate stressed conditions. Given the evidence that some cancer stem cells (CSCs) have bivalent metabolism while the bulk of the non-stem cancer cells is glycolytic (Peiris-Pages et al., 2016), it remains to be seen if both eIF4E-dependent and -independent translation pathways are present in CSCs and if simultaneously blocking both pathways may be beneficial for eradicating CSCs.

CDK1 may also play important roles in promoting OXPHOS and eIF4E-independent translation in naive hESCs. Previous studies showed that CDK1 can phosphorylate and activate multiple mitochondrial electron transport chain complex proteins to promote OXPHOS while activating p53, MnSOD, and SIRT3 to eliminate reactive oxygen species (ROS) and maintain mitochondrial homeostasis (Wang et al., 2014b; Xie et al., 2019). Furthermore, our bioinformatics analysis indicates that multiple eIFs particularly eIF2 subunits are potential substrates for CDK1. In ESCs (Friend et al., 2015) and adult stem cells (Zismanov et al., 2016), phosphorylated eIF2A is found to inhibit global translation but stimulate the translation of selective mRNAs containing upstream open reading frames (uORFs) within their 5′-UTR, and uORFs can increase IRES-mediated translation in a transcript-specific manner (Leppek et al., 2018). Therefore, potential phosphorylation of eIFs by CDK1 may facilitate the translation of a subset of mRNAs via eIF4E-independent pathways. In contrast to elevated CDK1 activity, the mTORC1 signaling in RSet-ff naive hESCs is significantly suppressed, resulting in a much reduced level of total 4EBP and phosphorylated 4EBP, and a much lesser dependence of naive hESC on mTORC1 signaling-mediated eIF4E-dependent translation. Interestingly, CDK1 and mTORC1 share common substrates and may compete with each other for the same subset of substrates (Odle et al., 2020), and therefore it remains to be determined if a CDK1-to-mTORC1 switch may account for or be associated with the naïve-to-primed pluripotency transition.

Fourthly, we revealed that a more robust proteome quality control system is present in naive hESCs that ensures a high-fidelity and compact proteome for rapid cell cycling. Remarkably, protein folding chaperon and proteasome are the 2 most up-regulated pathways in naive hESC proteome we identified. The quality of the proteome is known to be controlled by the proteostasis surveillance system, a complex network of integrated cellular pathways that maintain a balanced protein synthesis rate, high protein synthesis accuracy, efficient protein folding, and continual tagging of damaged proteins for degradation (Francisco et al., 2020). Noormohammadi et al. (Noormohammadi et al., 2016) showed that human pluripotent stem cells exhibit increased assembly of the chaperonin TRiC/CCT complex that assists the folding of a significant percentage of nascent protein such as actin and tubulin, and serves as a key quality control mechanism to maintain an intact and refined proteome for active self-renewal. Our finding that CCT subunits are significantly up-regulated in naive over primed hESCs clearly indicate a more efficient protein folding system operating in naive pluripotency. In addition, the ubiquitin/proteasome-mediated protein degradation machinery (but not the overall protein degradation) and autophagy system are also up-regulated in naive hESCs. It was shown that autophagy supports the translation of a subset of mRNAs enriched for cell cycle control and DNA damage repair (Goldsmith et al., 2020). All these proteostasis surveillance mechanisms form a tighter proteome quality control system for human naive pluripotency.

Taken together, when compared with primed hESCs, we reveal several important attributes for naive hESCs that include their proteomes with reduced total protein quantities and PTM levels, their strengthened proteome quality control system, and their expedited cell cycling. We show that bivalent metabolism allows for translating selective mRNAs via both eIF4E-dependent and eIF4E-independent/eIF4A2-dependent pathways that jointly generate a refined and compact proteome required for robust self-renewal and survival of naive hESCs. These findings may assist in better understanding the recently revealed unrestricted lineage potential of naive hESCs and in further optimizing their induction and differentiation conditions for future clinical applications.

## Limitations of the study

Although three naive hESC induction systems were employed in parallel to validate some of the identified key targets, the primary multi-omics analyses were performed with H9 cells induced by the RSet-ff system. We did notice minor differences and inconsistencies in the results obtained with the three systems. As the RSet-ff system is known to give rise to the so-called “intermediate” naive hESCs, ideally, similar studies with “bona fide” naive hESCs derived from the PXGL system can be conducted which may further consolidate the key findings of this study as well as reveal exciting novel findings. Among the identified differential pathways and targets, we validated only a few of them, such as CDK1, eIF family members, mTOR, and its substrates. Many important findings including the higher expression of IGF1R and phosphorylation of AKT in naive hESCs await further validation and in-depth mechanistic studies. Site-directed mutagenesis studies may be employed to investigate the differentially phosphorylated or acetylated sites in some critical target proteins.

## STAR★Methods

### Key resources table

**Table:** 

REAGENT or RESOURCE	SOURCE	IDENTIFIER
**Antibodies**		

Anti-KLF4	Abcam	Cat# ab215036
Anti-H3K27me3	Cell signaling	Cat#9733; RRID:AB_2616029
Anti-OCT4A	Cell signaling	Cat#2890; RRID:AB_2167725
Anti-SOX2	Cell signaling	Cat#2748s; RRID:AB_823640
Anti-NANOG	Cell signaling	Cat#4903s; RRID:AB_10559205
Anti-CDK1	Beyotime	Cat#AF1516
Anti-IgG	ABclonal	Cat#AC005; RRID:AB_2771930
Anti-Phospho-CDK1(Thr^14^)	Beyotime	Cat#AF5758
Anti-Phospho-CDK1(Thr^15^)	ABclonal	Cat# AP0016; RRID:AB_2770978
Anti-Phospho-CDK1(Thr^161^)	Beyotime	Cat#AF5764
Anti-α-Tubulin	GNI Group	Cat#GNI4310-AT
Anti-E-cadherin	Beyotime	Cat#AF0138
Anti-N-cadherin	Cell signaling	Cat#13116p; RRID:AB_2687616
Anti-Beta-catenin	Cell signaling	Cat#8480p; RRID:AB_11127855
Anti-δ-Catenin/p120 Catenin	ABclonal	Cat#A11399; RRID:AB_2861557
Anti-biotin	Cell signaling	Cat#7075; RRID:AB_10696897
Anti-mTOR	Cell signaling	Cat#2983; RRID:AB_2105622
Anti-Phospho-mTOR(Ser^2448^)	Cell signaling	Cat#5536; RRID:AB_10691552
Anti-PRAS40	Cell signaling	Cat#2691; RRID:AB_2225033
Anti-Phospho-PRAS40(Thr^246^)	Cell signaling	Cat#2997; RRID:AB_2258110
Anti-4EBP1	Cell signaling	Cat#9644; RRID:AB_2097841
Anti-Phospho-4EBP1(Thr^37/46^)	Cell signaling	Cat#2855; RRID:AB_560835
Anti-eIF4E	Cell signaling	Cat#2067; RRID:AB_2097675
Anti-β-Actin	GNI Group	Cat#GNI4310-BA
Anti-eIF4G1	Abcam	Cat#ab47649; RRID:AB_2246259
Anti-eIF3A	Abcam	Cat#ab86146; RRID:AB_2096634
Anti-eIF4B	Beyotime	Cat#AF6774
Anti-eIF4A1	ABclonal	Cat#A11584; RRID:AB_2861601
Anti-eIF4A2	Abcam	Cat#ab31218; RRID:AB_732123
Anti-eIF2α	Beyotime	Cat#AF6771
Anti-eIF2A	ABclonal	Cat#A9709; RRID:AB_2863762
Anti-eIF5A	Beyotime	Cat#AF2008
Anti-Phospho-GSK3β	Cell signaling	Cat#5558; RRID:AB_10013750
Anti-Phospho-ERK	Cell signaling	Cat#4370; RRID:AB_2315112
Anti-IGF1R	Cell signaling	Cat#3027; RRID:AB_2122378
Anti-Phospho-IGF1R(Tyr^1135/1136^)	Cell signaling	Cat#3024; RRID:AB_331253
Anti-AKT	Cell signaling	Cat#4691; RRID:AB_915783
Anti-Phospho-AKT(Thr^308^)	Cell signaling	Cat#2965; RRID:AB_2255933
Anti-Phospho-AKT(Ser^473^)	Cell signaling	Cat#4060; RRID:AB_2315049
Anti-rabbit IgG, HRP-Linked Antibody	Cell signaling	Cat#7074; RRID: AB_2099233
Anti-mouse IgG,HRP-Linked antibody	Cell signaling	Cat#7076; RRID: AB_330924
Alexa Fluor® 488 AffiniPure Donkey Anti-Mouse IgG (H+L)	Jackson ImmunoResearch	Cat#715-545-150; RRID:AB_2340846
Alexa Fluor® 594 AffiniPure Donkey Anti-Mouse IgG (H+L)	Jackson ImmunoResearch	Cat#715-585-150; RRID:AB_2340854
Alexa Fluor® 594 AffiniPure Donkey Anti-Rabbit IgG (H+L)	Jackson ImmunoResearch	Cat#711-585-152; RRID:AB_2340621
Alexa Fluor® 488 AffiniPure Donkey Anti-Rabbit IgG (H+L)	Jackson ImmunoResearch	Cat#712-545-153; RRID:AB_2340684
Anti-GFP	Beyotime	Cat# AG279-1
Anti- IgG-PE	BioLegend	Cat#400114
Anti- IgG-FITC	BioLegend	Cat#400208
Anti- SUSD2-PE	BioLegend	Cat#327406; RRID:AB_940654
Anti- CD24-FITC	BioLegend	Cat#311103; RRID:AB_314852

**Chemicals, peptides, and recombinant proteins**		

Matrigel	Corning	Cat#354277
0.1% Gelatin	Biological Industry	Cat#01-944-1B
Geltrex, growth factor-reduced	Thermo Fisher Scientific	Cat# A1413302
CryoStor® CS10	StemCell	Cat#07930
mTeSR1 medium	StemCell	Cat#85850
RSeT Feeder-Free medium	StemCell	Cat#05975
DMEM/F12	Sigma	Cat#D6421
Neurobasal	Thermo Fisher Scientific	Cat#21103049
Opti-MEM	Thermo Fisher Scientific	Cat#31985062
B27	Thermo Fisher Scientific	Cat#17504044
N2	Thermo Fisher Scientific	Cat#17502048
L-Glutamine	Thermo Fisher Scientific	Cat#25030024; CAS:56-85-9
non-essential amino acids	Thermo Fisher Scientific	Cat#11140050
β-mercaptoethanol	Macklin	Cat#M6230; CAS: 60-24-2
0.02% EDTA	Cienry	Cat#E6511; CAS: 6381-92-6
TrypLE	Thermo Fisher Scientific	Cat#12605010
Accutase	Thermo Fisher Scientific	Cat# A1110501
PD0325901	ABCR	Cat# AB253775; CAS: 391210-10-9
XAV939	Sigma	Cat#X3004; CAS: 284028-89-3
Gö6983	Bio-Techne	Cat#2285; CAS: 133053-19-7
Recombinant Human Leukemia Inhibitory Factor	Sangon Biotech	Cat# C600146
valproic acid sodium salt	Sigma	Cat# P4543; CAS: 1069-66-5
Bovine Serum Albumin	Sigma	Cat#B2064; CAS: 9048-46-8
Dasatinib	Selleckchem	Cat#S1021; CAS: 302962-49-8
AZD5438	Tocris	Cat#3968; CAS: 602306-29-6
SB590885	R&D systems	Cat#2650; CAS: 405554-55-4
RO3306	TargetMol	Cat#T2356; CAS: 872573-93-8
Cycloheximide (CHX)	Sigma-Aldrich	Cat# 239763-M; CAS: 66-81-9
XL388	TargetMol	Cat#T6030; CAS: 1251156-08-7
4E1RCat	TargetMol	Cat#T1742; CAS: 328998-25-0
Lonidamine	TargetMol	Cat#T0239; CAS: 50264-69-2
GSK2837808A	TargetMol	Cat#T15435; CAS: 1445879-21-9
Y27632	Selleck	Cat#S1049; CAS: 129830-38-2
TRIzol reagent	Life Technologies	Cat#15596-026
iTaq Universal SYBR Green Supermix	Bio-Rad	Cat#1725124
TritonX-100	Diamond	Cat#A110694
4% formaldehyde	Sigma-Aldrich	Cat#F8775
Laemmli Sample Buffer	Bio-Rad	Cat#1610747
SDS Lysis Buffer	Beyotime	Cat#P0013G
ECL	Share-Bio	Cat#sb-wb012
Coomassie blue- R250	Sangon Biotech	Cat#A100472; CAS: 6104-59-2
Saponin	Sangon Biotech	Cat#A604521; CAS: 8047-15-2
FBS	GIBCO	Cat#10270-106
T4 DNA ligase	New England Biolabs	Cat# M0202
Sodium dodecyl sulfate (SDS)	Sangon Biotech	Cat#A600485; CAS: 151-21-3
Acetonitrile	Sigma-Aldrich	Cat# 900667; CAS: 75-05-8
Trypsin	Promega	Cat#V5113
Protease and Phosphatase Inhibitor Single-Use Cocktail	Thermo Fisher Scientific	Cat#78443
Anti-acetylation antibody beads	Cell signaling	Cat#13416
Hoechst 33342	Thermo Fisher Scientific	Cat#H21492
Cell Staining Buffer	BioLegend	Cat#420201
Fc Receptor Blocking Solution	BioLegend	Cat#422301
7-AAD Viability Staining Solution	BioLegend	Cat#420403
Protein A/G Magnetic Beads	MedChemExpress	Cat# HY-K0202
Lipofectamine® RNAiMAX Reagent	Thermo Fisher Scientific	Cat#13778075
Lipofectamine 3000	Thermo Fisher Scientific	Cat# L3000015
lentiCas9n(D10A)-Blast	Addgene	Cat#63593
psPAX2	Addgene	Cat#12260
pMD2.G	Addgene	Cat#12259

**Experimental models: Cell lines**		

Human (h) male ESC line H1	Cell Bank of the Chinese Academic of Sciences	SCSP-301
Human (h) female ESC line H9	Cell Bank of the Chinese Academic of Sciences	SCSP-302
DR4 MEF	Cell Bank of the Chinese Academic of Sciences	SCSP-108R
HEK293T cells	Cell Bank of the Chinese Academic of Sciences	SCSP-502

**Critical commercial assays**		

PrimeScript RT reagent kit	TaKaRa	Cat#DRR047S
Cell Cycle and Apoptosis Analysis Kit	Beyotime	Cat#C1052
AP staining kit	Beyotime	Cat#C3206
EZ-press RNA Purification Kit	EZ Bioscience	Cat#B0004DP
BCA Protein Assay Kit	Bio-Rad	Cat#P0011
Enhanced Cell Counting Kit-8	Beyotime	Cat#C0043
Click-iT™ Plus OPP Alexa Fluor™ 488 Protein Synthesis Assay Kit	Thermo Fisher Scientific	Cat#C10456
EZ DNA Methylation-Gold Kit	Zymo Research	Cat#D5005
2G Robust HotStart PCR Kit	Kapa Biosystems	Cat#KK5522
High-Select™ TiO2 Phosphopeptide Enrichment Kit	Thermo Fisher Scientific	Cat#A32993

**Deposited data**		

Multi-omics data deposited at ProteomeXchange	This paper	PXD030230

**Software and algorithms**		

MaxQuant	http://www.coxdocs.org/doku.php?id=maxquant:start	Version 1.6.0.16
Image J	http://imagej.nih.gov/ij	Version 1.52
MethPrimer	http://www.urogene.org/methprimer2/	Version 2.0
ModFit LT		Version 4.0.5
GraphPad Prism 7	https://www.graphpad.com/scientific-software/prism/	Version 7
Perseus	http://www.coxdocs.org/doku.php?id=perseus:start	Version 1.6.0.7
Cytoscape	https://cytoscape.org/	Version 3.7.2
Simcap		Version 14.1
STRING	https://string-db.org/	N/A
R		Version 3.6.1
Venny	http://bioinfogp.cnb.csic.es/tools/venny/index.html	Version 2.1.0
Motif-X algorithm	http://meme-suite.org/tools/momo	N/A
CELLO	http://cello.life.nctu.edu.tw/	Version 2.5
FastQC	http://www.bioinformatics.babraham.ac.uk/projects/fastq	N/A
Trimmomatic	Bolger et al., 2014	Version 0.36
Metilene	http://www.bioinf.uni-leipzig.de/Software/metilene/	Version 0.2-7
IKAP	Mischnik et al., 2016	N/A
MATLAB		Version R2004a

### Resource availability

#### Lead contact

Further information and requests for resources and reagents should be directed to and will be fulfilled by the Lead Contact, Ying-Jie Wang (yingjiewang@zju.edu.cn).

#### Materials availability

This study did not generate new unique reagents.

### Experimental model and subject details

#### Human cell lines and drug treatment

Human (h) male ESC line H1 (SCSP-301) and female ESC line H9 (SCSP-302) were purchased from the Cell Bank of the Chinese Academic of Sciences, Shanghai, China. Details for all the key resources were summarized, and sequences for all the qPCR primers were presented in Table S7. Where appropriate, hESCs were treated with RO3306 (TargetMol, #T2356), Cycloheximide (CHX) (Sigma-Aldrich, #239763-M), XL388 (TargetMol, #T6030), 4E1RCat (TargetMol, #T1742), Lonidamine (LONI) (TargetMol, #T0239), or GSK2837808A (GSKA) (TargetMol, #T15435) either individually or in combination with the indicated concentrations.

#### Primed and RSet-ff naiïve hESC culture

Here hESCs were cultured under feeder-free conditions to eliminate potential confounding effects caused by irradiated mouse embryonic fibroblast (MEF) feeder layers. Primed hESCs were grown on Matrigel (Corning, #354277) coated tissue culture dishes according to manufacturer's recommendation, and maintained in mTeSR1 media (StemCell Technologies, #85850) at 37°C and 5% O_2_. Only before RSet-ff naive induction, primed hESCs were cultured in 20% O_2_ for a week. Medium change was performed on daily basis. Cells were passaged every 3 to 4 days by 5 min incubation at 37°C with 0.02% EDTA (Cienry, #E6511). Conversion of primed hESCs to naive-like hESCs in RSeT™ Feeder-Free medium was achieved following the manufacturer's manual (StemCell Technologies, #05975). Briefly, one day after plating aggregates of primed hESCs (cultured in 20% O_2_), the mTeSR1 medium was replaced with RSeT™ Feeder-Free medium and cells were transferred to hypoxia environment. Afterward, the cultures were replaced with fresh RSeT™ Feeder-Free medium every two days until the domed-shaped colonies appeared (approximately 5-7 days). The naïve-like hESCs were passaged using TrypLE (Thermo Fisher, #12605010).

#### PXGL nNaïve hESC culture

N2B27 medium (1 L): 487 mL DMEM/F12 (Sigma, #D6421), 487 mL Neurobasal (Thermo Fisher, #21103049), 10 mL B27 (Thermo Fisher, #17504044), 5 mL N2 (Thermo Fisher, #17502048), 10 mL L-Glutamine (200 mM, Thermo Fisher, #25030024), 1 mL 0.1M β-mercaptoethanol (Macklin, #M6230).

PXGL medium: N2B27 (in house) supplemented with 1 μM PD0325901 (ABCR, #AB253775), 2 μM XAV939 (Sigma, #X3004), 2 μM Gö6983 (Bio-Techne, #2285) and 10 ng/mL human LIF (Sangon Biotech, #C600146). MEFs (multi-drug resistant DR4 strain) were inactivated by irradiation and used at a density of 1.5 × 10^4^/cm^2^. Primed hESCs were cultured in 5% O_2_, 5% CO_2_ in a humidified incubator at 37°C.

Conversion of primed hESCs to naive hESCs in PXGL medium was achieved following the protocol previously published (Bredenkamp et al., 2019). Primed hESCs were seeded at 1 × 10^4^/cm^2^ onto irradiated MEF feeders in mTeSR1 medium. Two days later (day 0), medium was changed to PDL/HDACi (N2B27 with 1 μM PD0325901, 10 ng/mL human LIF and 1 mM valproic acid sodium salt (VPA, Sigma, #P4543) (HDACi)). Following 3 days in PDL/HDACi, medium was changed to PXGL and refreshed daily for a further 10 to 11 days before passaging to establish naïve hESC cultures in PXGL medium on irradiated MEF feeders.

The cultures are heterogeneous initially, with many differentiated cells, and a few passages may be required until cultures appear homogeneous. After initial passages on MEF, stabilized reset cells can subsequently be maintained on Geltrex (growth factor-reduced, Thermo Fisher, #A1413302). The derived naïve hESCs can be dissociated with TrypLE Express and purified. Then, plating cell suspension and immediately adding 1 μLl/cm^2^ Geltrex to each well and mixing by gentle shaking. ROCK inhibitor (Y-27632) was added during replating. Next day feeding cells with fresh PXGL medium. There is no need to add additional Geltrex. Cultures can be split every 5 days at a ratio of 1:3, and cells can be cryopreserved using CryoStor® CS10 (StemCell Technologies, #07930) and stored at liquid nitrogen temperature (−135°C).

#### FINE naïve hESC culture

Conversion of primed hESCs to naive hESCs in FINE medium was achieved following the protocol previously published (Szczerbinska et al., 2019). Primed cells were seeded on 200× diluted Matrigel at a passage ratio of 1:6. Cells were seeded in clumps and kept in mTeSR1 culture for 48 h. To convert the primed hESCs to naive hESCs, we removed mTeSR1 medium and added FINE culture medium, which consists of a basal medium (1:1 ratio of DMEM/F12 (Sigma, #D6421) and Neurobasal (Thermo Fisher, #21103049) medium, 1x N2 supplement (Thermo Fisher, #17502048), 1× B27 supplement (Thermo Fisher, #17504044), 1x L-glutamine (Thermo Fisher, #25030024), 1× non-essential amino acids（Thermo Fisher #11140050）, 0.1 mM β-mercaptoethanol (Macklin, #M6230), and 62.5 μg/mL BSA (Sigma,#B2064)) supplemented with 0.1 mM dasatinib (Selleckchem, #S1021), 0.1 mM AZD5438 (Tocris, #3968), 0.1 mM SB590885 (R&D systems, #2650), 1 mM PD0325901 (ABCR, #AB253775), 10 mM Y-27632 (Selleckchem, #S6390), 20 ng/mL human recombinant leukemia inhibitory factor (Sangon Biotech, #C600146), 20 ng/mL activin A (StemCell Technologies), and 8 ng/mL basic fibroblast growth factor (PeproTech)). Cells were incubated under normoxic conditions (20% O_2_, 5% CO_2_) for 4 to 5 days. FINE culture medium was replenished daily. At the end of conversion, cells were passaged as single cells using TrypLE solution on Geltrex-coated plates (dishes were coated for at least 1 h before use). In brief, cells were washed with 1× PBS, and 1 mL of TrypLE was added to each well (6-well plate, Corning). Cells were incubated at 37°C for 3–5 min. When cells started to detach from each other and remained adherent to the plate, TrypLE was aspirated thoroughly. After adding 1 mL of FINE medium, cells were gently detached and clumps dissociated to single cells with a 1-mL pipette. Then cells were seeded at a high ratio of 1:2 in coated plates and transferred to a hypoxia (5% O_2_, 5% CO_2_) incubator for subsequent culture. Medium was refreshed daily. FINE culture cells were subsequently passaged at ratios of 1:2 to 1:4. Differentiated cells were observed in the first 2–3 passages and gradually decreased over subsequent passages. FINE naiïve hESCs were cultured in medium for at least five passages before use.

### Method details

#### Reverse transcription quantitative real-time PCR

Total RNA was extracted using TRIzol reagent (Life Technologies, #15596-026) and complementary DNA (cDNA) made from 1 μg of RNA using PrimeScript RT reagent kit with gDNA eraser (TaKaRa, #DRR047S). The sequences of all the primers used were presented in key resources table. For real-time PCR, we used iTaq Universal SYBR Green Supermix (Bio-Rad, #1725124) and Step One Plus (Applied Biosystems). All the PCR amplification was performed in triplicate and repeated in three independent experiments.

#### Immunofluorescence microscopy

hESCs were washed twice with PBS and fixed with 4% formaldehyde (Sigma, #F8775) in PBS for 15 min at room temperature, washed three times with PBS and permeabilized with 0.2% Triton X-100 (Diamond, # A110694) in PBS at room temperature for 10 min. They were then washed three times with PBS and blocked with 3% BSA (Sigma, #A9418) in PBS for 45 min. Thereafter, cells were incubated with specified primary antibodies overnight at 4°C and secondary antibodies for 1 h at room temperature, with three washes in between, and visualized with a Nikon A1R confocal microscope.

#### Flow cytometry

Cells were digested by Accutase (Thermo Fisher, #A1110501) for 3 min and single cell suspension was prepared in Cell Staining Buffer (BioLegend, # 420,201). After adding 5 μL of Fc Receptor Blocking Solution (BioLegend, #422301) per 100 μL of cell suspension, cells were incubated for 5 to 10 min at room temperature. Cell samples in solution were added with appropriately conjugated fluorescent primary antibodies at predetermined optimum concentrations, and incubated on ice for 15 to 20 min in the dark. Cell samples were washed twice with at least 2 mL of Cell Staining Buffer by centrifugation at 350 g for 5 min. Finally, cell pellets were resuspended in 0.5 mL of Cell Staining Buffer and added with 5 μL (0.25μg)/million cells of 7-AAD Viability Staining Solution (BioLegend, #420403) to exclude dead cells. After cell samples were incubated on ice for 3 to 5 min in the dark, flow cytometry analyses were performed using an ACEA NovoCyteTM (ACEA Biosciences). The following antibodies were used for flow cytometry: IgG-PE (BioLegend, #400114), IgG-FITC(BioLegend, #400208), SUSD2-PE (BioLegend, #327406), CD24-FITC (BioLegend, #311104).

#### Immunoblotting

Collected cell samples were lysed with the Laemmli Sample Buffer (BIO-RAD, #1610747). 20 μL of loading samples were subjected to 10% SDS–PAGE and then transferred onto 0.45 μm polyvinyl difluoride membranes (Millipore, #IPVH00010). After blocking in 5% skim milk, membranes were probed with the indicated primary antibodies and corresponding secondary antibodies. Finally, the blots were developed using the ECL reagent (Share-Bio, #sb-wb012) and visualized by the Tanon 5200 Multi Chemiluminescent Imager system.

#### Cell cycle analysis

Cell cycle analysis of hESCs treated with RO3306 or vehicle was performed using Cell Cycle and Apoptosis Analysis Kit (Beyotime, #C1052). Cells were collected and washed with cold PBS. They were then fixed with pre-cooled 70% ethanol for 30 min at 4°C. After incubated in PI stain solution for 30 min at 37°C, flow cytometry analyses were performed using an ACEA NovoCyteTM (ACEA Biosciences) and ModFit LT (V.4.0.5).

#### Immunoprecipitation

Immunoprecipitating antibodies (1.6 μg of anti–CDK1 (Beyotime, #AF1516); 1.6 μg of anti-IgG (ABclonal, #AC005)) were added to the PBST (1% Tween final concentration) together with Protein A/G magnetic beads (20 μL of magnetic bead volume, MedChemExpress, # HY-K0202) for 2 h at 4°C. Then, cells (two 10-cm-diameter dishes per condition) were lysed (800 μL per dish) with cell scraper. Cell lysates in lysis buffer (20 mM Tris-HCl (pH 7.5), 100 mM KCl, 5 mM MgCl_2_, 0.5% NP-40, protease inhibitor (Thermo, #78443) and 0.1% DTT) were shaked for 15 seconds at 5 min intervals for 30 min on ice. After centrifugation (14,000 rpm for 15 min at 4°C), the supernatants were added with magnetic beads (PBST was removed) and incubated overnight at 4°C. Magnetic beads carrying immunoprecipitated immune complexes were then collected and washed three times in lysis buffer. Samples were denatured at 100°C for 8 min in 40 μL of Laemmli Sample Buffer (BIO-RAD, #1610747). After removing beads by magnetic separation, samples were subjected to SDS-PAGE and Western blotting, and the blots were developed by the Tanon 5200 Multi Chemiluminescent Imager system.

#### Transfection and viral transduction

For siRNA transfection, primed hESCs were digested into single cell suspension and plated at a density of 1–1.5 × 10^5^ cells/well (H9) or 2 × 10^5^ cells/well (H1) in 24-well plates with ROCK inhibitor Y27632 treatment. After 24 h, cells were then starved for 3 h in Opti-MEM (Thermo, #31985062) and transfected with siRNAs (scramble siRNA as control) by Lipofectamine RNAiMAX Reagent (Thermo, #13778075) according to the manufacturer's instructions. Medium was refreshed after 6 h. For detection of mRNA or protein levels, cells were further cultured for 48 h. For pluripotency resetting, transfected primed hESCs were further manipulated in different naïve induction systems.

Primed or naïve hESCs stably overexpressing EGFP (as control) or CDH1 were generated by lentivirus-mediated transduction. The LentiCDH1-Blast plasmid was derived from the lentiCas9n(D10A)-Blast (Addgene, #63593) by replacing sequence of Cas9n(D10A) with cDNA sequence of the CDH1 gene. The control LentiEGFP-Blast plasmid was constructed in a similar fashion. Then, lentiviruses were generated in HEK293T cells (SCSP-502, from the Cell Bank of the Chinese Academic of Sciences) by co-transfecting the HEK293 cells with psPAX2 (Addgene, #12260), pMD2.G (Addgene, #12259), and LentiCDH1-Blast (or LentiEGFP-Blast) plasmids using Lipofectamine 3000 (Thermo, L3000015). 6 to 8 h after transfection, the medium was replaced with Opti-MEM containing 5% FBS and 1% sodium pyruvate. The culture media containing the released recombinant viruses were collected on day 1 after transfection, fresh media were added, and the culture media were collected again on day 2 after transfection. The recombinant virus-containing supernatants were filtered with a 0.45-μm filter and viral particles were concentrated by centrifugation at 25,000 rpm in a Beckman SW 28 rotor for 100 min at 4°C. hESCs were subsequently transduced with concentrated viruses for 12 h and cultured in fresh media for further 48 h before blasticidin was added.

#### Colony-formation assay and alkaline phosphatase staining

To compare the single-cell clonogenicity of primed versus naïve hESCs, primed and naïve hESCs were digested into single cells and plated at a density of 10000 cells/well in 6-well plates without ROCK inhibitor Y27632 treatment and cultured for a week. Next, the alkaline phosphatase (AP) staining kit (Beyotime, #C3206) was used to stain the hESC colonies, and then the number of colonies was quantified by Image J (V.1.52).

To assess their pluripotency after RO3306 treatment, H9N-EGFP and H9N-CDH1 cells were plated at a density of 50000 cells/well in 6-well plates without ROCK inhibitor Y27632 treatment and cultured for 4 days. Cells were then treated with DMSO (Ctrl) or 5 μM RO3306 for 24 h and AP staining was performed as mentioned above.

#### Cell doubling-time determination

8 × 10^4^ of naïve and primed hESCs were seeded in 12-well plates. Cells were digested and counted using the Countess II FL Automated Cell Counter (Thermo) at 24, 48, 72, 96, 120, 144 and 168 h after seeding. Growth curves were plotted using the GraphPad Prism 7.0 software. Doubling time was calculated using the following formula:

Doubling time = duration ∗ log (2)/[log (final concentration)-log (initial concentration)].

#### Quantification of RNA and protein abundance

5 × 10^5^ of naïve and primed hESCs were counted and collected repeatedly for the following assays. Total RNA was isolated by EZ-press RNA Purification Kit (EZ Bioscience, #B0004DP), and its quality was determined using a Nano Drop 2000 (Thermo) and quantitated by absorbance at 260 nm. For protein extraction, hESCs were lysed with SDS Lysis Buffer (Beyotime, #P0013G) and centrifuged at 12,000 g for 5 min. Then, the supernatant was collected and the protein concentration was determined with BCA Protein Assay Kit (Bio-Rad, #P0011) according to the manufacturer's instructions. In parallel, total proteins were subjected to SDS-PAGE and visualized by Coomassie Blue staining (0.1% Coomassie blue- R250 (Sangon Biotech, #A100472), 10% acetic acid, 25% isopropanol) after cells were treated with CHX for 12 h. Then, the overall band intensities representing total protein levels in individual lanes were quantified by the ImageJ software.

#### Cell viability assay

hESCs were seeded at a density of 5,000 cells in a volume of 100 μL per well in 96-well plates. The next day, medium was replaced with fresh culture medium containing XL388 or RO3306 at the indicated concentrations. The cells were cultured in the presence of XL388 for 4 h (RO3306 for 24 h) and then subjected to CCK-8 analysis (Beyotime, #C0043). The OD450 values were measured by a microplate reader (Thermo).

#### Determination of global nascent protein synthesis rate

Click-iT™ Plus OPP Alexa Fluor™ 488 Protein Synthesis Assay Kit (Thermo, #C10456) was used to determine global protein synthesis/translation rate. After treatment with inhibitors at indicated concentrations, hESCs were incubated for 30 min in the medium supplemented with O-propargyl-puromycin (OPP) (20 μM), a puromycin analog that generates covalent conjugates with nascent polypeptide chains. Cells were then harvested and fixed with 1% paraformaldehyde for 15 min on ice. Next, the fixed cells were permeabilized in PBS supplemented with 0.1% saponin (Sangon Biotech, #A604521) and 3% FBS (GIBCO, #10270-106) for 5 min at room temperature. The copper-catalyzed azide-alkyne cycloaddition was performed according to the manufacturer's protocol. Addition of the Alexa Fluor^TM^ 488 picolyl azide and the ‘click’ reaction reagents led to a ‘click’ reaction between the picolyl azide dye and the OPP alkyne, allowing the newly synthesized polypeptides to be labeled with Alexa Fluor^TM^ 488. Finally, the Alexa Fluor^TM^ 488-labeled cell samples were re-suspended in 100 μL PBS supplemented with 3% FBS and 0.1% saponin per well in 96-well plates and analyzed by an Operetta CLS High-Content Analysis System (PerkinElmer). All fields in each well were imaged and the mean Alexa Fluor^TM^ 488 fluorescence intensity was quantified. Mean background fluorescent intensity (referred to as mBFI) was the average of all background fluorescent intensity in each field. The global nascent protein synthesis rate for a given sample was calculated as the mean Alexa Fluor^TM^ 488 fluorescence intensity subtracted by mBFI. The ratio of global nascent protein synthesis rate with an inhibitor treatment over that with vehicle (control) treatment was calculated as: (mean Alexa Fluor^TM^ 488 fluorescent intensity with a specific inhibitor - mBFI)/(mean Alexa Fluor™ 488 fluorescent intensity with vehicle - mBFI).

#### Next generation sequencing-based bisulfite sequencing PCR

Gene-specific DNA methylation was assessed by a next generation sequencing-based BSP, according to a previously published method (Gao et al., 2014). Briefly, BSP primers were designed using the online MethPrimer software. 1 μg of genomic DNA was converted using the ZYMO EZ DNA Methylation-Gold Kit (Zymo Research, #D5005) and one-20th of the elution products were used as templates for PCR amplification with 35 cycles using KAPA 2G Robust Hot start PCR Kit (Kapa Biosystems, #KK5522). For each sample, BSP products of multiple genes were pooled equally, 5′-phosphorylated, 3′-dA-tailed and ligated to barcoded adapter using T4 DNA ligase (New England Biolabs, #M0202). Barcoded libraries from all samples were sequenced on Illumina platform.

#### LC-MS sample preparation

All MS experiments were performed in biological triplicates. Proteins were extracted from hESCs using SDT lysis buffer (4% SDS, 100 mM DTT, 100 mM Tris-HCl pH 8.0) with 1× Halt Protease and Phosphatase Inhibitor Cocktail (Thermo, #78443). Samples were boiled for 5 min, and further ultrasonicated and boiled for another 5 min. Undissolved cellular debris were removed by centrifugation at 16,000 g for 15 min. The supernatant was collected and quantified with a BCA Protein Assay Kit (Bio-Rad, #P0011).

#### Protein digestion

Protein digestion was performed with the FASP method as described by Wisniewski et al. (Wisniewski et al., 2009). Briefly, the detergent, DTT and IAA in UA buffer were added to block reduced cysteine. Then, the protein suspension was digested with trypsin (Promega, #V5113) at a ratio of 50:1 overnight at 37°C and collected by centrifugation at 16,000 g for 15 min. Finally, the peptides were desalted with a Sep-Pak C 18 cartridge (Waters, #Z720070) for further analysis.

#### Affinity enrichment

Phosphopeptides were enriched using High-Select™ TiO_2_ Phosphopeptide Enrichment Kit (Thermo, #A32993). Briefly, lyophilized peptide samples were resuspended in Binding/Equilibration buffer and applied to the pre-equilibrated TiO_2_ Spin Tip. After washing, enriched phosphopeptides were eluted using the Phosphopeptide Elution Buffer. Then, the eluates were dried immediately in a speed vacuum concentrator to remove elution buffer. For acetylated peptide enrichment, the peptide samples were first dissolved in IPA buffer and incubated with prewashed anti-acetylation antibody beads (PTMScan® Acetyl-Lysine Motif [Ac-K] Kit, Cell Signaling Technology, #13416) at 4°C for 1.5 h incubation. Then the beads were washed three times with IPA buffer and three times with ddH_2_O. The bound peptides were eluted from the beads with 0.1% trifluoroacetic acid (TFA), and then, dried by vacuum concentrator. Finally, enriched acetylated peptides were desalted with a Sep-Pak C 18 cartridge and vacuum-dried again.

#### LC-MS/MS analysis

LC-MS/MS was performed on a Q Exactive Plus mass spectrometer coupled with Easy 1200 nLC (Thermo Fisher Scientific). Peptides were first loaded to a trap column (100 μm∗20 mm, 5 μm, C18) in buffer A (0.1% (v/v) Formic acid in water). Reverse-phase high-performance liquid chromatography (RP-HPLC) separation was performed with the EASY-nLC system (Thermo Fisher Scientific) using a self-packed column (75 μm × 150 mm; 3 μm ReproSil-Pur C18 beads, 120 Å, Dr. Maisch GmbH) at a flow rate of 300 nL/min. The RP−HPLC mobile phase A was 0.1% formic acid in water, and B was 0.1% formic acid in 95% acetonitrile. Peptides were eluted over 120 min with a linear gradient of buffer B. The gradient was 5% B for 2 min and was linearly increased to 8% in 88 min, and then increased to 23% in 10 min, and then increased to 40% in 8 min and maintained for 12 min. MS data were acquired using a data-dependent top20 method dynamically choosing the most abundant precursor ions from the survey scan (300–1800 m/z) for HCD fragmentation. The instrument was run with peptide recognition mode enabled. A lock mass of 445.120025 Da was used as internal standard for mass calibration. The full MS scans were acquired at a resolution of 70,000 at m/z 200, and 17,500 at m/z 200 for MS/MS scan. The maximum injection time was set to 50 ms for MS and 50 ms for MS/MS. Normalized collision energy was 27 and the isolation window was set to 1.6 Th. Dynamic exclusion duration was 60 s.

### Quantification and statistical analysis

#### Sequence database searching and mass spectrometric data analysis

We processed the mass spectra using MaxQuant software version 1.6.0.16. MS data were searched against the UniProtKB Homo sapiens database (173,282 total entries). The trypsin was selected as the digestion enzyme. The maximal two missed cleavage sites and the mass tolerance of 4.5 ppm for precursor ions and 20 ppm for fragment ions were defined for database search. Carbamidomethylation of cysteines was defined as a fixed modification, while acetylation of protein N-terminal and oxidation of Methionine were set as variable modifications for database searching. The database search results were filtered and exported with <1% false discovery rate (FDR) at peptide-spectrum-matched level, and protein level, respectively. For phosphorylation or acetylation data searching, phosphorylation on serine, threonine, and tyrosine or acetylation on lysine was further added into the searching parameter and the site localization probability was set as > 0.75.

#### Bioinformatics analysis

To assess the quality of data, principal-component analysis (PCA) and relative standard deviation (RSD) were performed using simcap (v.14.1) and R (v.3.6.1), respectively. Only proteins with fold change ≥1.5-fold or ≤0.66-fold and a p value <0.05 were considered as significantly differential hits. As for phosphoproteome and acetylome, only modified sites with a localization probability of >0.75 were further analyzed. Venn diagrams for differential proteins and modified sites were drawn by Venny 2.1.0 (http://bioinfogp.cnb.csic.es/tools/venny/index.html). Hierarchical clustering analysis was performed with the pheatmap package, which is based on the open-source statistical language R25, using Euclidean distance as the distance metric and complete method as the agglomeration method. Besides, R was also applied to perform volcano plot analysis with ggplot2 package.

#### Bisulfite sequencing data analysis

The FastQC tool (http://www.bioinformatics.babraham.ac.uk/projects/fastq) was used to perform basic statistics on the quality of the raw reads. Then, sequencing adapters and low quality data of the sequencing data were removed by Trimmomatic (version0.36) (Bolger et al., 2014). The BSMAP software was used to map the bisulfite sequence to the reference genome with parameters ‘-n 0 -g 0 -v 0.08 -m 50 -x 1000’ (Xi and Li, 2009). The statistic information of the alignment was collected, only the unique mapped reads were kept for the following analysis, and only methylated cytosines with sequence depth coverage of at least 5 were used. If the base on the alignment is C, methylation occurs; conversely, if the base on the alignment is T, no methylation occurs. The methylation levels of individual cytosines were calculated as the ratio of the sequenced depth of the ascertained methylated CpG cytosines to the total sequenced depth of individual CpG cytosines, i.e., ML = mC/(mC + umC). Where ML is the methylation level, mC and umC represent the number of reads supporting methylation C and the number of reads supporting unmethylated C, respectively. The software metilene (version 0.2-7) was used to identify DMR (differentially methylated regions) by a binary segmentation algorithm combined with a two-dimensional statistical test (parameters: -M 300 -m 5 -d 0.1 -t 1 -f 1 -v 0.7) (Juhling et al., 2016). Gene Ontology (referred to as GO, http://www.geneontology.org/) enrichment analysis of DMR-related genes were applied to uncover biological processes of interest, we choose to deem pathways with a Q value ≤0.05 as significantly enriched with DMR-related genes. Based on the results of the DMR annotation and the database of Kyoto Encyclopedia of Genes and Genomes (KEGG) (Kanehisa et al., 2008), functional enrichment analysis was performed on genes whose gene body and its upstream and downstream regions (upstream 2k, gene body, and downstream 2k) are overlapping with DMR.

#### Motif analysis

Over-represented sequence motifs from the total phosphoproteome and acetylome dataset were determined by motif-X algorithm (Schwartz and Gygi, 2005), available online at website: http://meme-suite.org/tools/momo. The motifs were centered at the modified residues (S, T, Y or K), using a ±10 amino acid residue sequence window surrounding the modification sites and only motifs with p < 10^−10^ were allowed.

#### Subcellular location

Subcellular location annotation information for total as well as regulated proteins or modified sites were revealed by CELLO (V.2.5) (Yu et al., 2006). The proportion of each cellular component was calculated and presented as a pie chart.

#### Inference of kinase activities from phosphoproteomics (IKAP) analysis

IKAP was a machine learning algorithm to infer kinase activity based on the phosphorylation levels of substrates (Mischnik et al., 2016). The kinase-substrate table was downloaded from the PhosphoSitePlus database (Hornbeck et al., 2015). After normalization of phosphopeptide abundance by protein abundance, phosphoproteomic data was input into the IKAP algorithm, and implementation was carried out in MATLAB (R2004a). Kinases that are most likely to phosphorylate the identified phosphosites were inferred for two pluripotency states.

#### Protein-protein interaction (PPI) network analysis

We submitted the significantly differential proteins to the Search Tool for the Retrieval of Interacting Genes (STRING, http://string.embl.de/) database, and only validated interactions with the highest confidence score >0.9 were selected. Cytoscape software (V.3.7.2) was further applied to visualize modules with the closest internal links from the PPI network. Molecular Complex Detection (MCODE) plugin performed with degree cutoff = 2, node score cutoff = 0.2, k-core = 2, and max. depth = 100. Besides, KEGG enrichment analyses were carried out with ClueGO plugin and the global view of connections among various pathways was presented.

#### Statistical analysis

For Figure 1, Figure 3, Figure 6C, 1D, 3D, 3G, 3J, 6A, 6B, 6E, and 6F; Figures S1C, S1D, S5B, S5D, S5F, S6F, S6G, S8F, S8G, S9A, S10B, S10D, S10E, and S10F, the p values were calculated by two-tailed unpaired Student's t test using GraphPad Prism 7. Data represent the mean ± S.D. as indicated in figure legends. p values of less than 0.05 were considered as statistically significant. All statistics were shown as ∗p < 0.05, ∗∗p < 0.01, ∗∗∗p < 0.001.

## Data Availability

• The multi-omics data have been deposited to the ProteomeXchange Consortium via the PRIDE partner repository with the dataset identifier PXD030230. • This paper does not report original code. • Any additional information required to reanalyze the data reported in this paper is available from the lead contact upon request.

## Supplemental information

Document S1. Figures S1–S10, Data S1, and Table S5–S7

Table S1: Proteomic data-H9, related to Figure 2

Table S2: Acetylomic data-H9, related to Figure 2

Table S3: Phosphoproteomic data-H9, related to Figure 2

Table S4: RNA processing and translation analysis, related to Figure 5
