# Supplementary material for: Translational and post-translational control of human naïve versus primed pluripotency
Source: iScience. 2021 Dec 17;25(1):103645. doi: 10.1016/j.isci.2021.103645 (PMC8718978; doi:10.1016/j.isci.2021.103645)

iScience, Volume *25*

Supplemental information

Translational and post-translational control of human naïve versus primed pluripotency

Cheng Chen, Xiaobing Zhang, Yisha Wang, Xinyu Chen, Wenjie Chen, Songsong Dan, Shiqi She, Weiwei Hu, Jie Dai, Jianwen Hu, Qingyi Cao, Qianyu Liu, Yinghua Huang, Baoming Qin, Bo Kang, and Ying-Jie Wang

Figure S1. Generation of naïve H1 hESCs in RSet-ff medium, Related to Figure 1

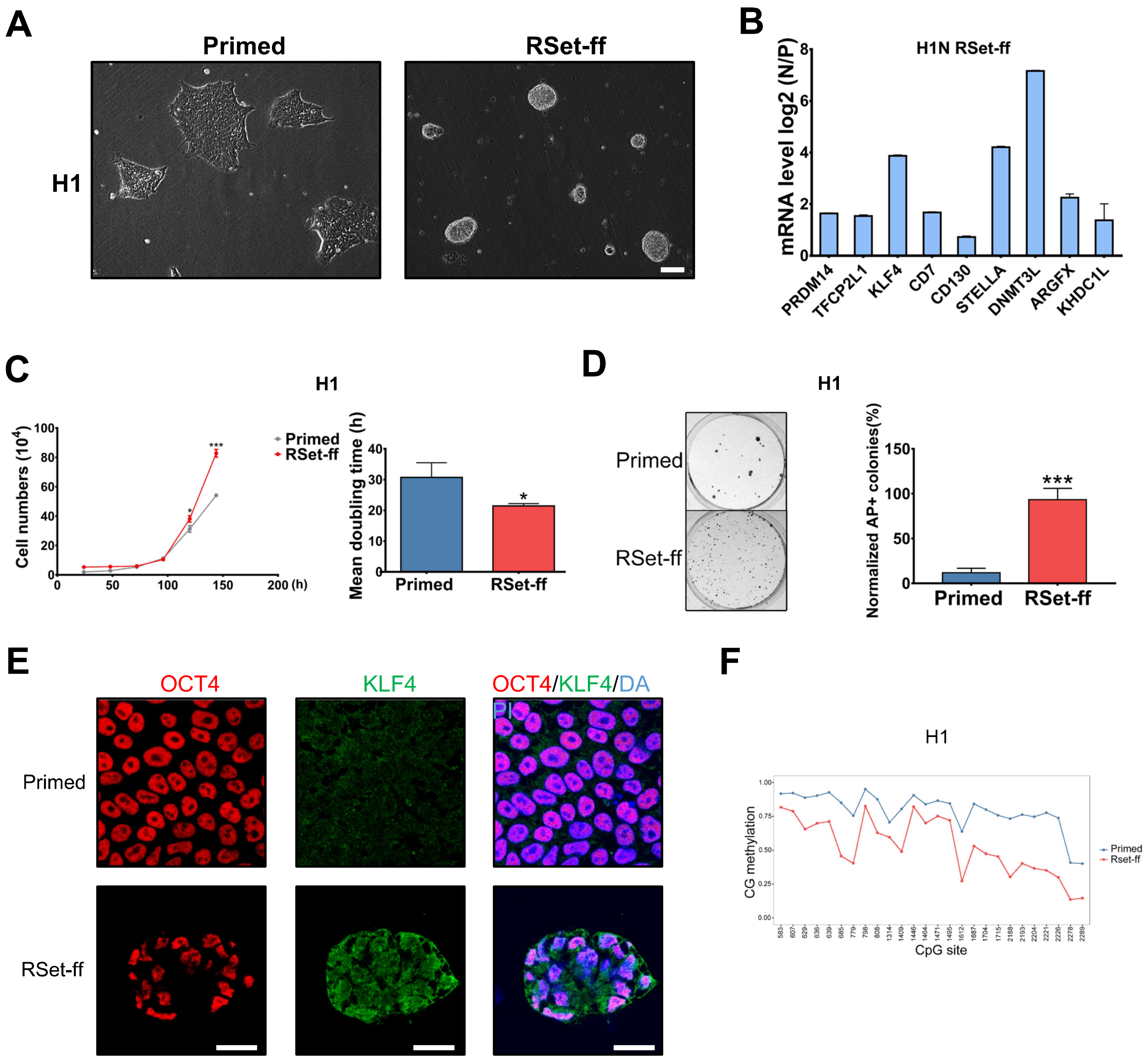

(A) Light micrographs of primed H1 hESCs and converted naïve H1 cells using RSet-ff system. Scale bars,100 μm.

(B) Primed and naive H1 hESCs were harvested, and the mRNA levels of naive-pluripotency marker genes were determined by qRT-PCR. The log2 Naïve/Primed (N/P) fold change values were presented as mean ± S.D. of three independent experiments.

(C) Mean population doubling time of naïve versus primed H1 hESCs. Total cell numbers (left panels) were counted every 24 hours, and the mean population doubling times were calculated (right panels). The data were presented as the mean ± S.D. from three independent experiments. Unpaired t test was performed so that *p <0.05, ***p <0.001.

(D) Single-cell clonogenicity of naïve versus primed H1 hESCs was measured by Alkaline Phosphatase Staining. The data were presented as the mean ± S.D. from three independent experiments. Unpaired t test was performed so that ***p <0.001.

(E) Immunofluorescence staining for pluripotency marker OCT4 and naive-associated marker KLF4. Scale bars, 50 μm.

(F) The CpG methylation level at the DNMT3L promotor in naïve (RSet-ff) versus primed H1 hESCs.

Figure S2. Establishment of PXGL and FINE naïve hESC culture systems, Related to Figure 1

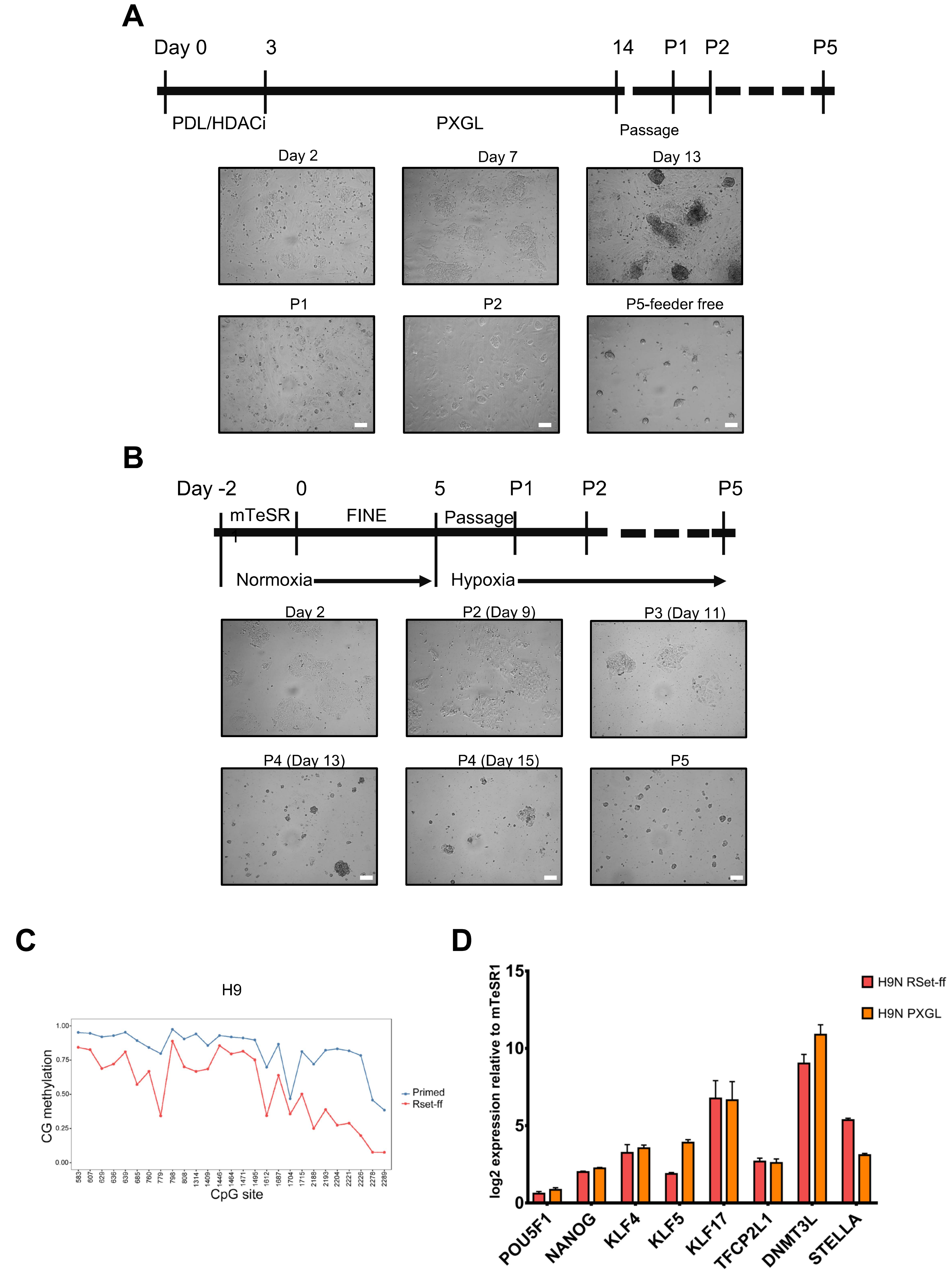

(A-B) Schematic representations of protocols and light micrographs of naïve H9 cells induced from primed H9 cells via PXGL (A) or FINE (B) inducing system. HDACi, histone deacetylase inhibitor. Scale bars, 200 µm.
(C) The CpG methylation level at the DNMT3L promotor in naïve (RSet-ff) versus primed H9 hESCs.
(D) Gene expression analysis for naive markers in RSet-ff- and PXGL-induced naïve H9 hESCs. Data were presented as mean ± S.D. of three independent experiments.

Figure S3. Global comparison of multi-omics parameters between primed and naïve (RSet-ff) H9 hESCs, Related to Figure 2

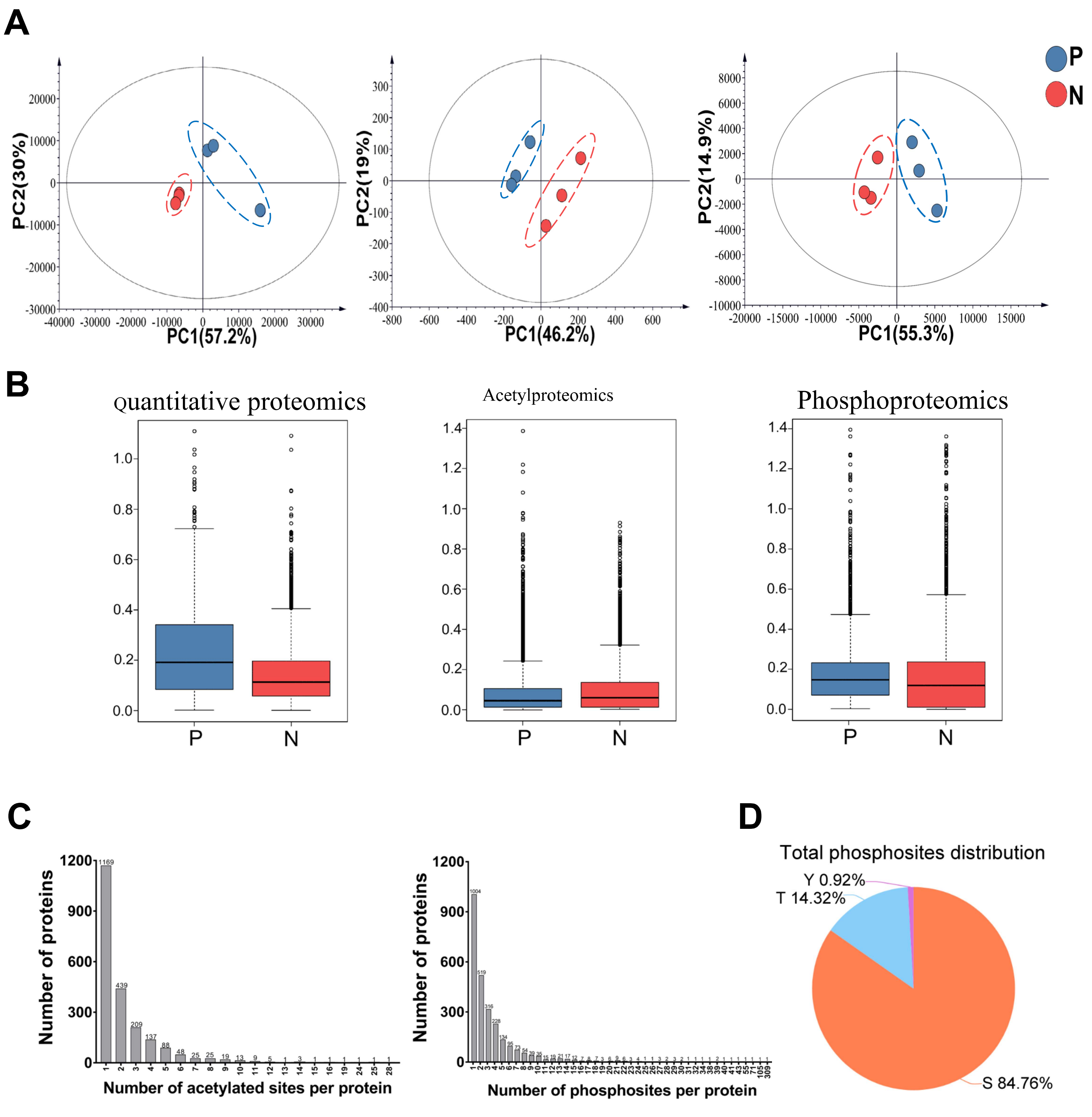

(A) Principal-component analyses for all identified proteins (left), acetylation sites (middle) and phosphosites (right) in primed (P) versus naïve (RSet-ff) (N) H9 hESCs.

(B) Relative standard deviations (RSDs) of quantitative values for three omics between primed (P) and naïve (RSet-ff) (N) H9 hESCs.

(C) Quantitative distribution of peptides containing phosphosites and acetylation sites.

(D) Classification of identified phosphosites.

Figure S4. Comparison of proteomics, phosphoproteomics and acetylproteomics profiles between primed and naïve (RSet-ff) H9 hESCs, Related to Figure 2

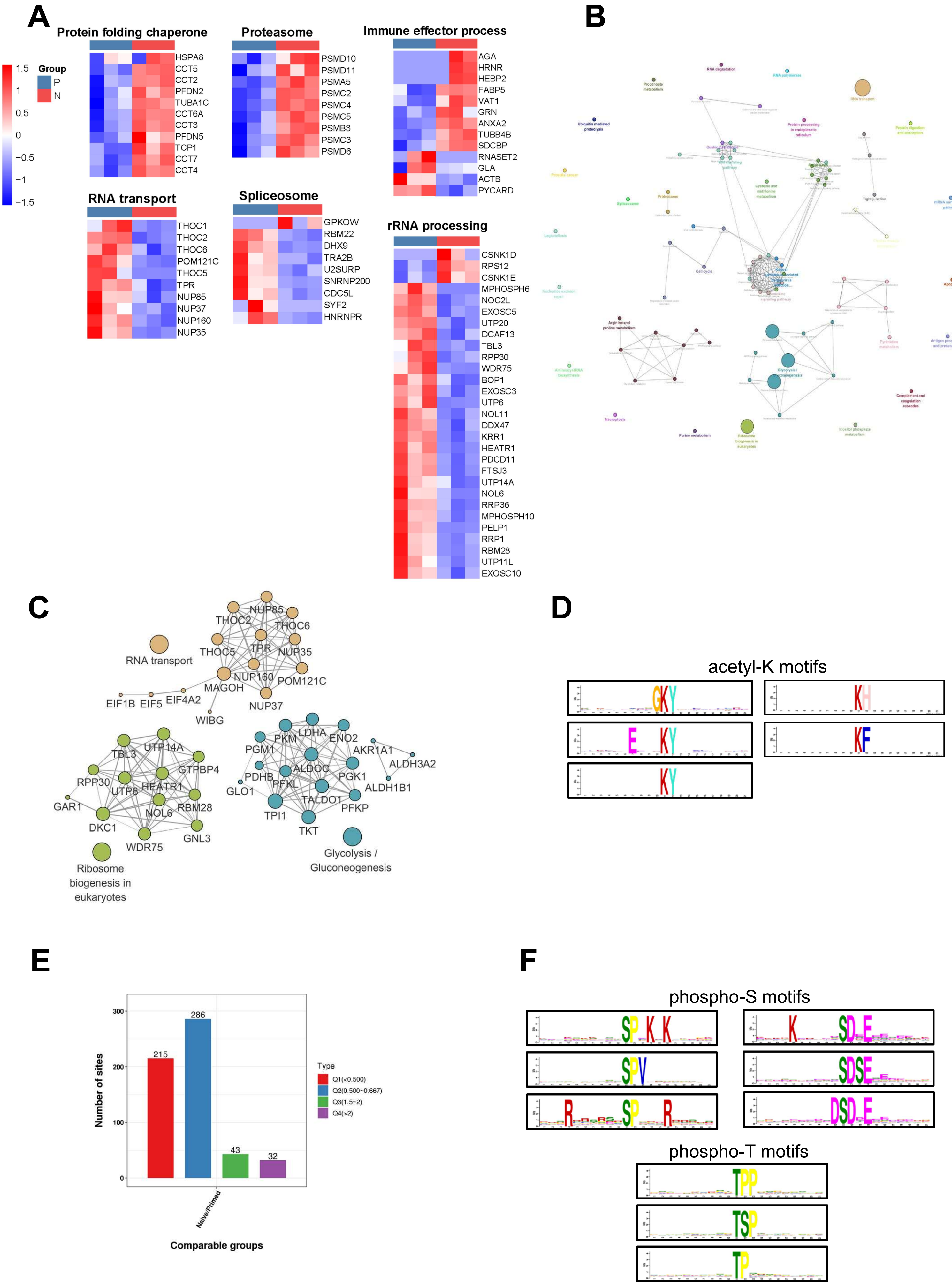

(A) Heatmap and cluster analyses for identified 6 modules with the upper 3 modules up-regulated in naïve hESCs and the lower 3 modules down-regulated in naïve hESCs.

(B) Global KEGG pathway connections of differentially expressed proteins in naïve versus primed hESCs.

(C) Three most enriched modules derived from global KEGG pathway analysis. The differential proteins and connections among them were shown for each module. The thickness of the lines represents the strength of the correlations.

(D) Motif analysis of acetylation sites present in naive hESCs by the MoMo.

(E) There are four quantiles indicating four different kinds of acetylation sites according to their quantitative ratio (naïve/primed), Q1 (ratio <0.5), Q2 ($0.5 <$ ratio <0.66), Q3 ($1.5 <$ ratio < 2), and Q4 (ratio >2).

(F) Motif analysis of phosphosites present in naive hESCs.

Figure S5. Ablating CDK1 activity had more prominent negative effect on naïve pluripotency than on primed pluripotency, Related to Figure 3

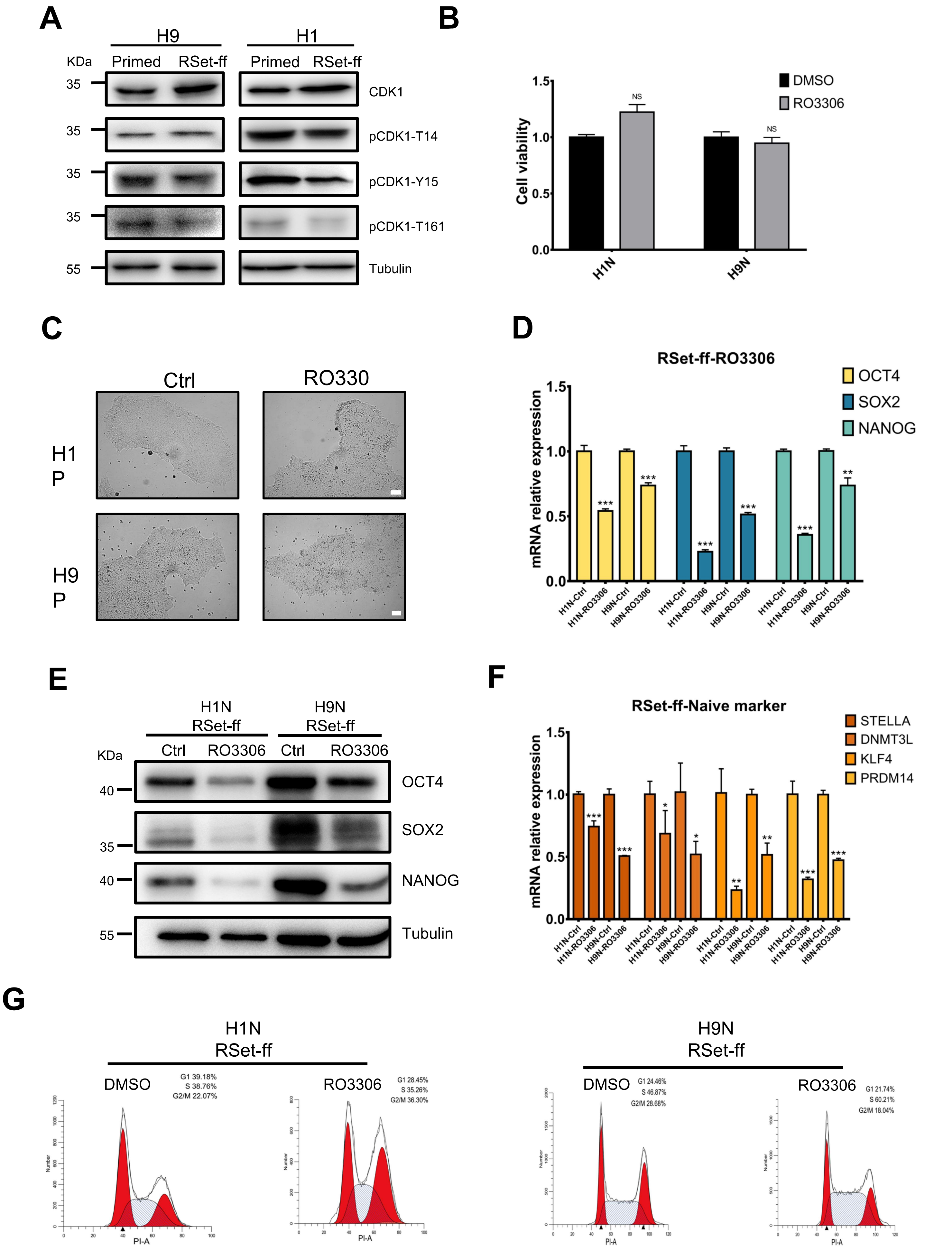

(A) Primed and naïve (RSet-ff) H9 hESCs were harvested and the whole cell lysates were subjected to SDS-PAGE and immunoblotting, and detected by specified antibodies. The immunoblots shown were from one experiment representative of three independent experiments with similar results.

(B) RSet-ff H1 and H9 naïve hESCs were treated with 5 μM RO3306 for 24 hours and subjected to CCK8 assay for cell viability assessment. Data were presented as mean ±S.D. from three independent experiments. Unpaired t test was performed so that ns $p > 0.05$.

(C) Light micrographs of primed H1 and H9 cells treated with vehicle (Ctrl) or 5 μM RO3306 for 24 hours. Scale bars, 200 μm.

(D) qRT-PCR detection of mRNA levels of pluripotency markers. The naïve (RSet-ff) hESCs were treated with 5 μM RO3306 for 24 hours. Data were presented as mean ±S.D. from three independent experiments. Unpaired t test was performed so that **$p < 0.01$, ***$p < 0.001$.

(E) Naïve (RSet-ff) H1 and H9 cells were harvested and the whole cell lysates were subjected to SDS-PAGE and immunoblotting, and detected by specified antibodies. The immunoblots shown were from one experiment representative of three independent experiments with similar results.

(F) Inactivation of CDK1 by RO3306 (5 μM for 24 hours) resulted in decreased transcription of naïve pluripotency markers. Naïve H1 and H9 cells were cultured in RSet-ff medium. Data were presented as mean ±S.D. from three independent experiments. Unpaired t test was performed so that *$p < 0.05$,**$p < 0.01$, ***$p < 0.001$.

(G) Naïve RSet-ff H1 and H9 cells were treated with vehicle (DMSO) or 5 μM RO3306 for 24 hours, fixed, stained with (50 μg/ml) Propidium Iodide, and subjected to flow cytometry for cell cycle analysis. The flow cytometry profiles presented were from one experiment representative of three independent experiments with similar results.

Figure S6. CDK1-regulated E-cadherin functionality is crucial for naïve pluripotency, Related to Figure 3

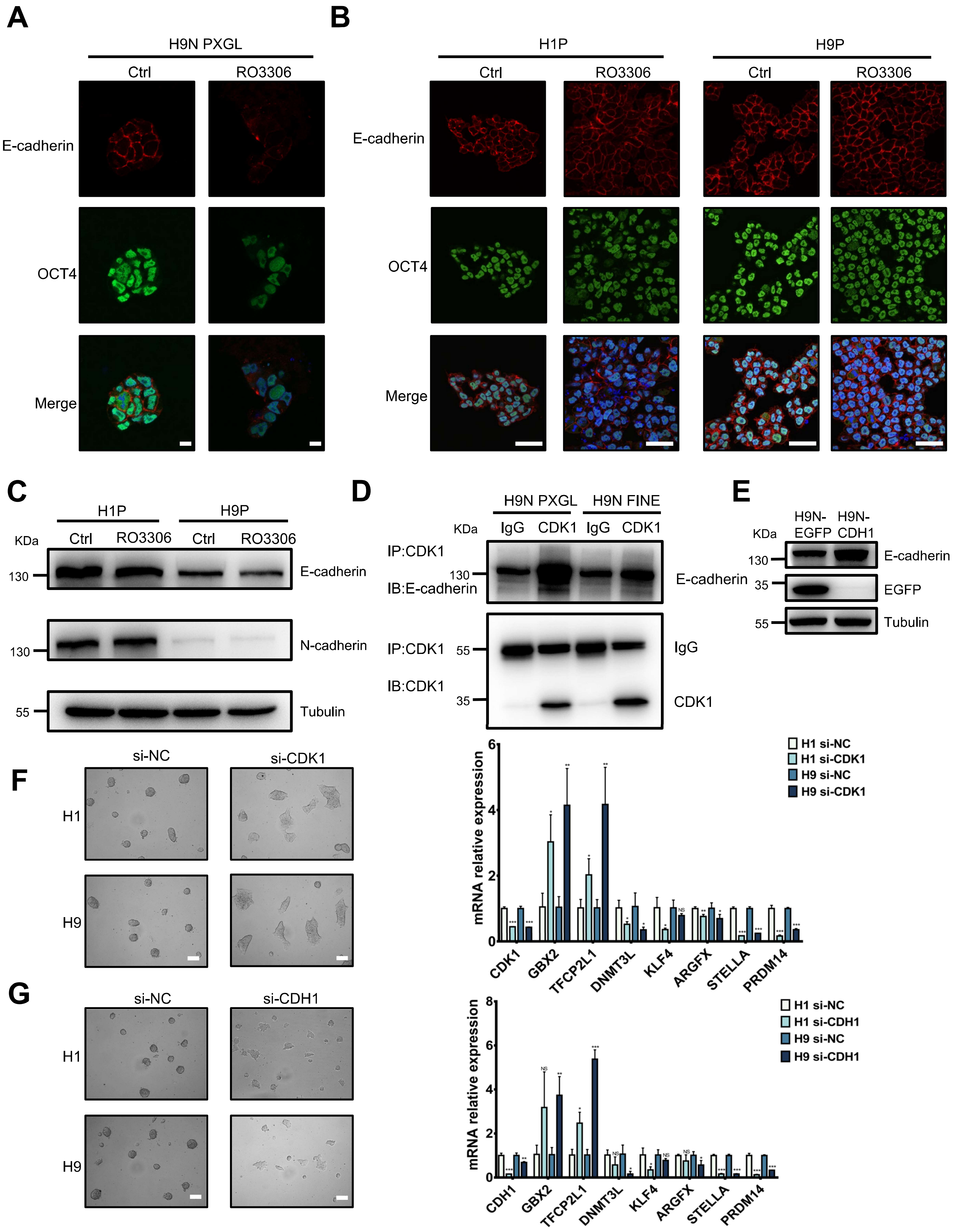

(A-B) PXGL-induced naïve H9 cells (A), primed H1 and H9 cells (B) were treated with vehicle (Ctrl) or 5 μM RO3306 for 24 hours, immunostained for E-cadherin and OCT4, and counterstained with Hoechst 33342 for nuclei. Representative confocal micrographs were shown. Scale bars, 10 μm (A), 50 μm (B).
(C) Western blot showing CDK1 inhibitor RO3306 had little effect on expression level of E-cadherin or N-cadherin. Primed H1 and H9 cells were treated with vehicle (Ctrl) or 5 μM RO3306 for 24 hours, harvested and the whole cell lysates were subjected to SDS-PAGE and immunoblotting with specified antibodies. The immunoblots shown were from one experiment representative of three independent experiments with similar results.
(D) CDK1 interacted with E-cadherin in naïve (PXGL/FINE) H9 hESCs. Naive cell lysates were immunoprecipitated with anti-CDK1 antibody, and the immune complexes were probed with anti-CDH1 antibody (upper) or anti-CDK1 antibody (lower).
(E) Western blot validation of overexpressed EGFP (control) and E-cadherin in lentiviral system-generated stable naïve (RSet-ff) H9 cells.
(F-G) CDK1 or CDH1 knockdown impaired establishment of naïve pluripotency. After being transfected with scramble siRNA (si-NC), si-CDK1, si-CDH1 for 24 hours, primed H1 and H9 hESCs were then cultured in RSet-ff medium to induce naïve pluripotency. After another 72 hours, cells were photographed (left) and the mRNA levels of naïve pluripotency biomarkers were determined by qRT-PCR (right). The changing trend of naïve markers was similar between si-CDK1 and si-CDH1. Scale bars, 200 μm. Data were presented as mean ±S.D. from three independent experiments. Unpaired t test was performed so that ns (not statistically significant) $p>0.05$, $*p <0.05$, $**p < 0.01$, $***p <0.001$.

Figure S7. Subcellular localization and KEGG pathway enrichment analysis of three omics, Related to Figure 4

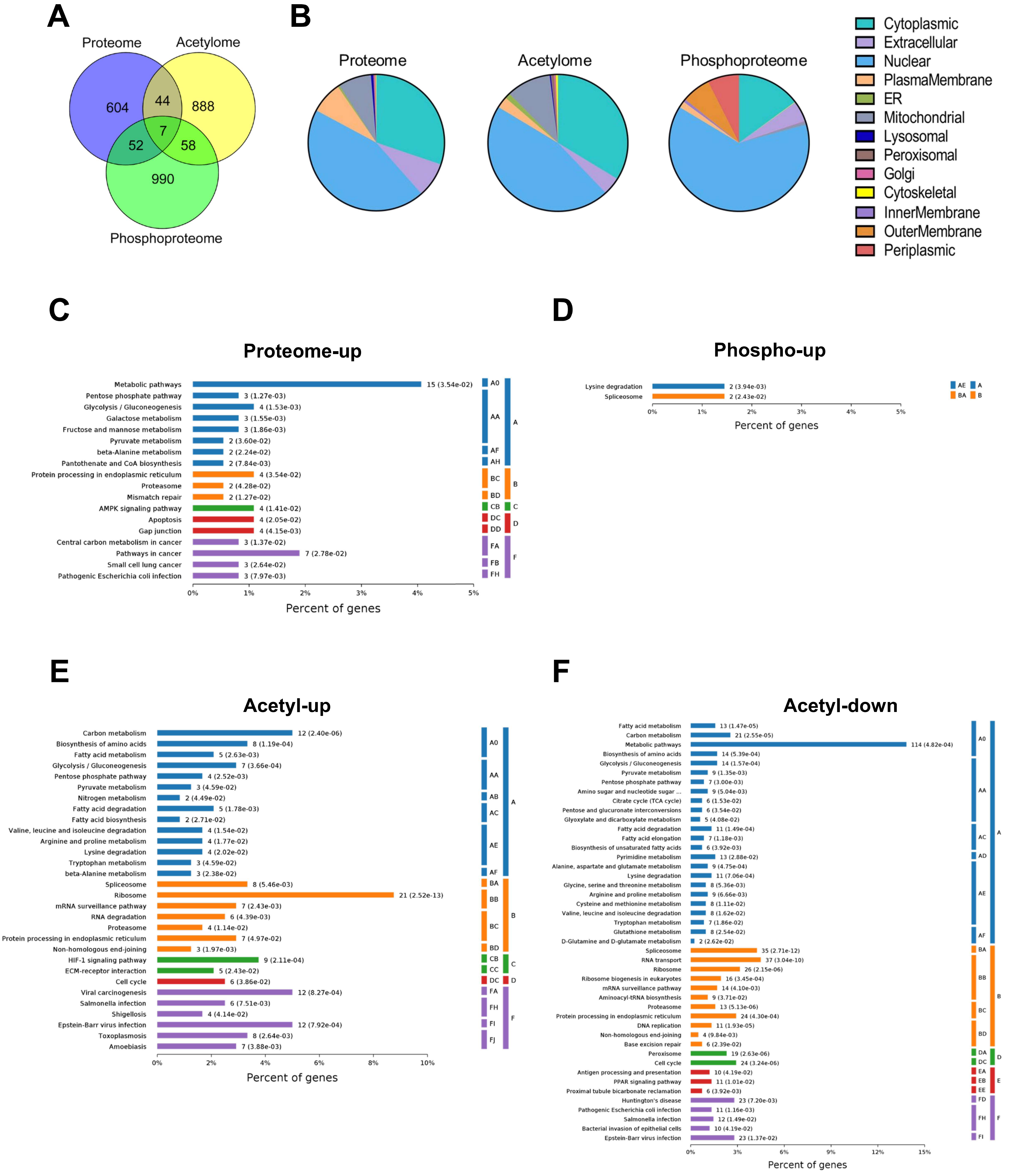

(A) Venn diagram showing the overlapping of differential proteomic, acetylomic and phosphoproteomic hits between naïve (RSet-ff) and primed H9 hESCs.
(B) Subcellular localization of total differentially changed proteins, acetylated and phosphorylated sites.
(C) KEGG pathway enrichment analysis of up-regulated protein (upper-left panel), up-regulated phosphosites (upper-right panel), up-regulated acetylation sites (lower-left panel), and downregulated acetylation sites (lower-right panel). Definition of acronyms: A-Metabolism: A0-Global and overview maps; AA-Carbohydrate metabolism; AB- Energy metabolism; AC-Lipid metabolism; AD-Nucleotide metabolism; AE-Amino acid metabolism; AF-Metabolism of other amino acids; AH-Metabolism of cofactors and vitamins; B-Genetic information processing: BA-Transcription; BB-Translation; BC-Folding, sorting and degradation; BD-Replication and repair; C-Environmental information processing: CB-Signal transduction; CC-Signaling molecules and interaction; D-Cellular processes: DA-Transport and catabolism; DC-Cell growth and death; DD-Cellular community; E-Organismal systems: EA-Immune system; EB-Endocrine system; EE-Excretory system; EF-Nervous system; F-Human Diseases: FA-Cancers: Overview; FB-Cancers: Specific types; FD-Neurodegenerative diseases; FH-Infectious diseases: Bacterial; FI-Infectious diseases: Viral; FJ: Infectious diseases: Parasitic.

Figure S8. Protein synthesis/turnover and the activity of mTORC1 in naïve versus primed hESCs, Related to Figure 6

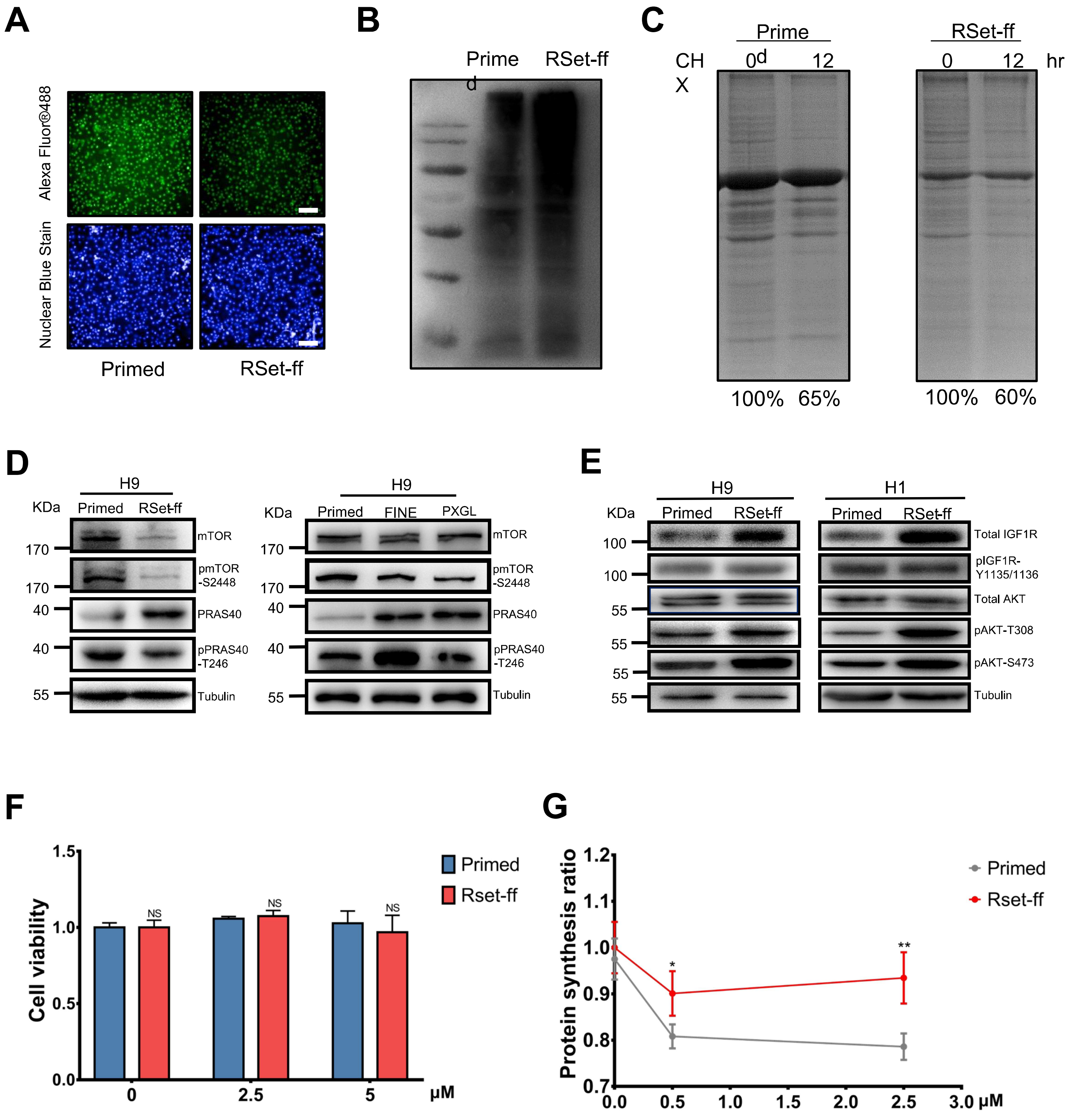

(A) Fluorescence pictures of primed and naïve (RSet-ff) H9 cells. Alexa FluorTM 488 fluorescence intensities indicated the quantities of incorporated nascent polypeptide chains. Scale bars, 100 μm

(B) Primed and naïve (RSet-ff) H9 hESCs were harvested and the whole cell lysates were subjected to SDS-PAGE and immunoblotting with anti-ubiquitin antibody.

(C) Primed and naïve (RSet-ff) H9 hESCs were treated with CHX (20 μg/ml) for 12 hours and subjected to SDS-PAGE and Coomassie Blue Staining.

(D) Primed and naïve H9 hESCs (RSet-ff, left; FINE and PXGL, right) were harvested and the whole cell lysates were subjected to SDS-PAGE and immunoblotting, and detected by specified antibodies. The immunoblots shown were from one experiment representative of three independent experiments with similar results.

(E) Primed and naïve (RSet-ff) H9 (left) and H1 (right) hESCs were harvested and the whole cell lysates were subjected to SDS-PAGE and immunoblotting, and detected by specified antibodies. The immunoblots shown were from one experiment representative of two independent experiments with similar results.

(F) Primed and naïve (RSet-ff) H9 hESCs were treated with varying concentrations of XL388 for 4 hours and subjected to CCK8 assay for cell viability assessment. Data were presented as mean ±S.D. from three independent experiments. Unpaired t test was performed so that ns $p>0.05$.

(G) Primed and naïve (RSet-ff) H9 cells were treated with XL388 at the indicated concentrations for 4 hours, and incubated with OPP and processed for determining nascent protein translation rates as described in Figure 6(B). The normalized mean Alexa FluorTM 488 fluorescence intensity indicative of incorporated nascent polypeptide chains was taken as the global nascent protein synthesis rate, and the ratio of global nascent protein synthesis rate with XL388 treatment over that with vehicle treatment was presented as mean ± S.D. from three independent experiments. Unpaired t test was performed so that *$p<0.05$,**$p<0.01$.

Figure S9. Naïve hESCs are more tolerant to eIF4E blockade, Related to Figure 6

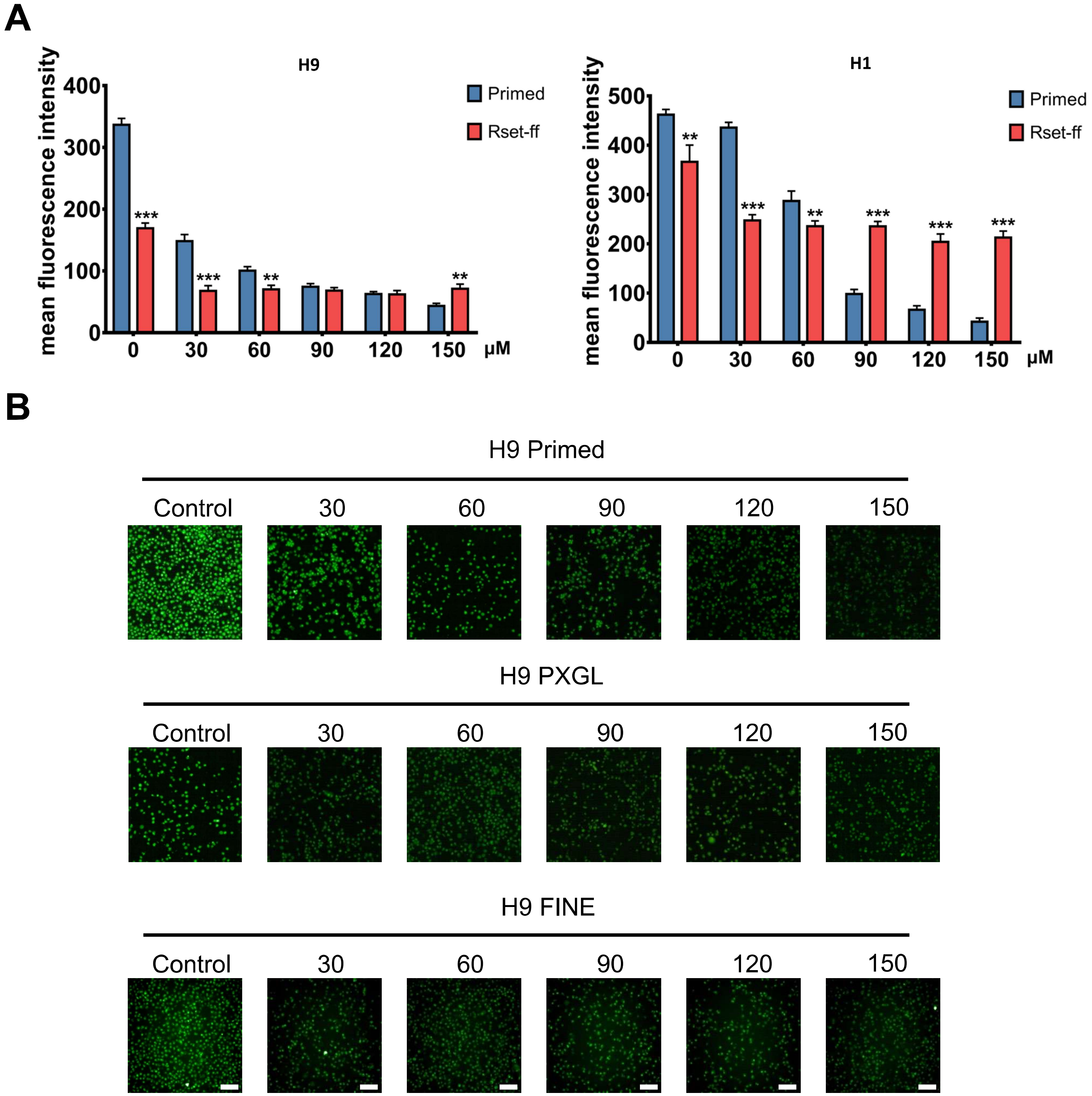

(A) Primed and naïve (RSet-ff) H9 (left) or H1 (right) cells were treated with varying concentrations of 4E1RCat for 2.5 hours, followed by nascent protein translation rate determination as described in Figure 6(B). The mean Alexa FluorTM 488 fluorescence intensity values indicative of incorporated nascent polypeptide chains were presented as mean ± S.D. of triplicate measurements from one experiment representative of three independent experiments with similar results. Unpaired t test was performed so that $^{**}p < 0.01$, $^{***}p < 0.001$.

(B) Fluorescence pictures of primed and naïve (PXGL/FINE) H9 cells treated with varying concentration of 4E1RCat for 2.5 hours. Alexa FluorTM 488 fluorescence intensities indicated the quantities of incorporated nascent polypeptide chains. Scale bars, 100 μm.

Figure S10. eIF4A2 and bivalent metabolism are essential for naïve pluripotency, Related to Figure 7

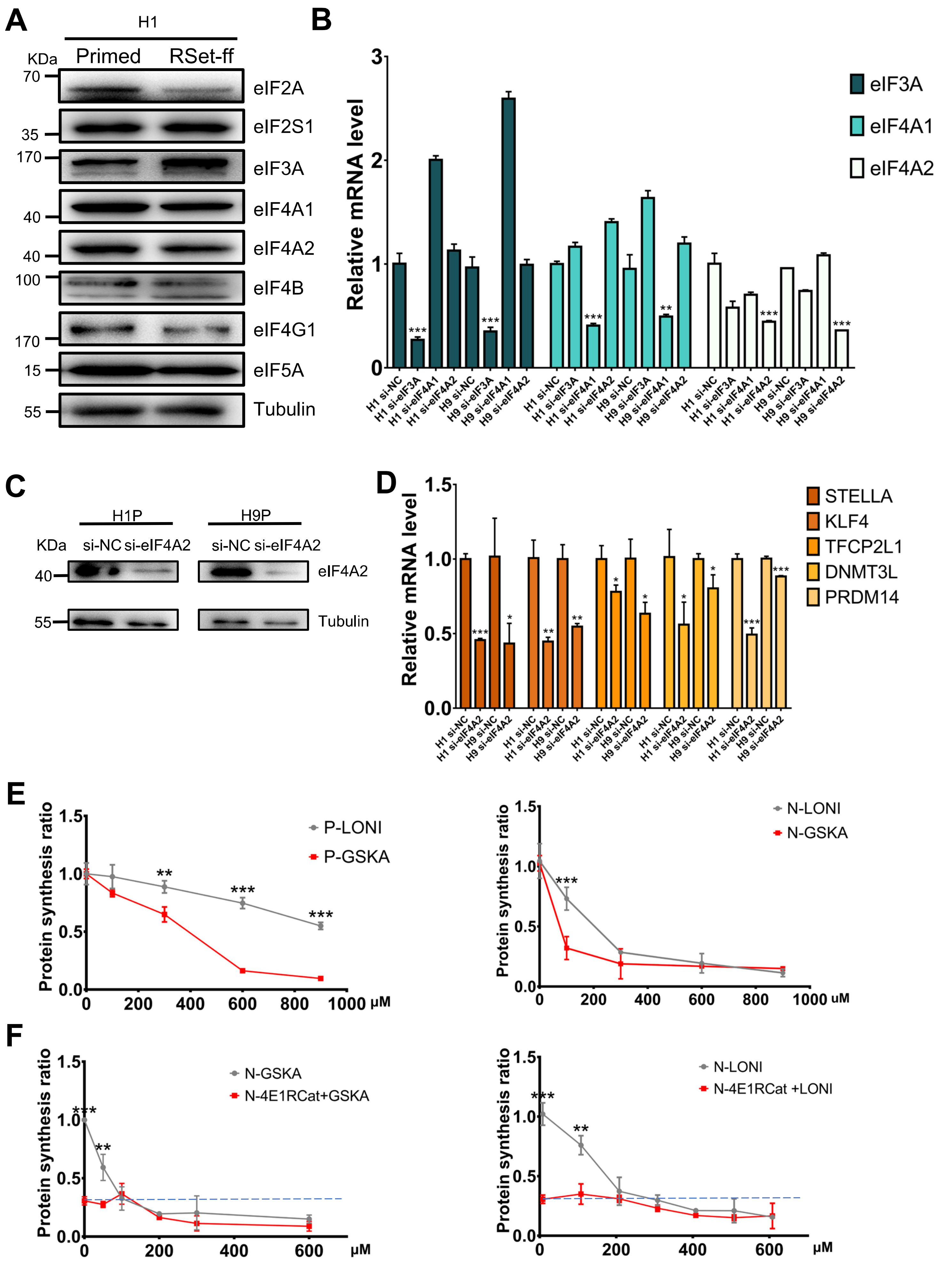

(A) Protein levels of a series of eukaryotic initiation factors in primed versus naïve (RSet-ff) H1 hESCs determined by Western blotting.

(B) qRT-PCR quantitation of mRNA levels for a series of eukaryotic initiation factors after primed H1 or H9 cells were transfected with indicated siRNAs for 48 hours. Data were presented as mean ± S.D. of triplicate measurements from one experiment representative of three independent experiments with similar results. Unpaired t test was performed so that **$p < 0.01$, ***$p < 0.001$.

(C) Western blot showing eIF4A2 protein levels were significantly reduced in primed hESCs after siRNA transfection for 48 hours.

(D) qRT-PCR quantitation of mRNA levels for naïve pluripotency marker genes. The primed hESCs were transfected with si-eIF4A2 and then cultured in RSet-ff medium for 48 hours. Data were presented as mean ± S.D. of triplicate measurements from one experiment representative of three independent experiments with similar results. Unpaired t test was performed so that *$p < 0.05$,**$p < 0.01$, ***$p < 0.001$.

(E) Primed or naïve (RSet-ff) H9 cells were treated with varying doses of Lonidamine (LONI) or GSK2837808A (GSKA) for 4 hours, followed by nascent protein translation rate determination as described in Figure 6(B). Data were presented as mean ± S.D. of triplicate measurements from one experiment representative of three independent experiments with similar results. Unpaired t test was performed so that **$p < 0.01$, ***$p < 0.001$.

(F) Naïve (RSet-ff) H9 cells were treated singly with varying doses of GSK2837808A (GSKA) or Lonidamine (LONI), or additionally in combination with a fixed dose (60 μM) of 4E1RCat for 4 hours, followed by nascent protein translation rate determination as described in Figure 6(B). The normalized mean Alexa FluorTM 488 fluorescence intensity indicative of incorporated nascent polypeptide chains was taken as the global nascent protein synthesis rate, and the ratio of global nascent protein synthesis rate with inhibitor treatment over that with vehicle treatment was presented as mean ± S.D. of triplicate measurements from one experiment representative of three independent experiments with similar results. Unpaired t test was performed so that **$p < 0.01$, ***$p < 0.001$. The dotted blue lines indicate the ratio level of global nascent protein synthesis rate with 60 μM 4E1RCat treatment alone over that with vehicle treatment.

Table S5-predicted CDK1 phosphorylation site in EIFs, Related to Figure 6

RNA transport	Uniprot ID	Protein description	N/P ratio in Proteome（FC）	N/P ratio in Proteome（P-VALUE）	N/P ratio in Phosphoproteome(p-value)	N/P ratio in Acetylome(P-value)	Predicted CDK1 phosphorylation site
DDX48;EIF4A3	A0A024R8W0;P38919;I3L3H2	Eukaryotic initiation factor 4A-III;Eukaryotic initiation factor 4A-III, N-terminally processed	0.694089139	0.030999876	S12-1.65(0.43)	/	
SUI1;EIF1	Q6IAV3;K7EM18;P41567	Eukaryotic translation initiation factor 1	/	/	S9-Only in primed	K66-Only in primed	
EIF1AX	P47813;X6RAC9	Eukaryotic translation initiation factor 1A, X-chromosomal	1.510322372	0.173340958	/	K56-1.15(0.76);K88-0.77(0.53)	
**EIF2A**	Q9BY44;F8WAE5;C9IZE1	Eukaryotic translation initiation factor 2A;Eukaryotic translation initiation factor 2A, N-terminally processed	0.789849491	0.308404287	S501-1.04(0.85)	/	T97 LATWQPYTTSKDGTA; T457 ALRNKPITNSKLHEE
EIF2AK2	Q8IW76;B7ZKK7;P19525;Q05CP4;Q6PK38	Interferon-induced, double-stranded RNA-activated protein kinase	0.821396002	0.621961874	/	/	
EIF2AK4	Q9P2K8	Eukaryotic translation initiation factor 2-alpha kinase 4	/	/	/	K1259-0.78(0.006);KK238-Only in primed	
EIF2B1	Q14232;H0YGG4;F5H0D0	Translation initiation factor eIF-2B subunit alpha	1.746457108	0.122309961	/	/	
EIF2B2	Q53XC2;P49770;H0YJJ8;G3V5E5;Q9BPX4	Translation initiation factor eIF-2B subunit beta	1.151972509	0.422055923	/	/	
EIF2B3	H0Y580;Q9HA31;Q9NR50	Translation initiation factor eIF-2B subunit gamma	only in naïve		/	/	S22 RMTDLTSSIPKPLLP
EIF2B5	Q13144	Translation initiation factor eIF-2B subunit epsilon	/	/	/	K110-only in primed	T336 DSTTQSCTHSRHNIY
EIF2S1	Q53XC0;P05198;G3V4T5;H0YJS4	Eukaryotic translation initiation factor 2 subunit 1	0.900470938	0.660988723	/	/	
**EIF2S2**	Q6IBR8;B5BU01;P20042;Q96I16	Eukaryotic translation initiation factor 2 subunit 2	1.24064711	0.250156085	S2-0.77(0.064)	K276-0.61(0.0005);K293-Only in primed	T11 DEMIFDPTMSKKKKK; S13 MIFDPTMSKKKKKKK
EIF2S3;EIF2S3L	P41091;Q2VIR3	Eukaryotic translation initiation factor 2 subunit 3;Putative eukaryotic translation initiation factor 2 subunit 3-like protein	1.060446734	0.731288491	/	K183-0.81(0.03);K54-0.64(0.005);K449-Only in primed	
EIF2C2;AGO2	A4FVC0;Q9UKV8	Protein argonaute;Protein argonaute-2	/	/	S336-only in primed	/	
EIF3A;eIF3a	Q24JU4;Q14152;J9R021;Q7Z5T5;Q6P1R0;Q05BS0;Q3B770	Eukaryotic translation initiation factor 3 subunit A	1.583754156	0.008377253	S881-Only in naïve	K1369-1.14(0.62)	S261 EDIHGLFSLSKKPPK
EIF3B;EIF3S9	B4DV79;A4D210;A0A024R821;P55884;B4DXN6;Q96G38;Q86UM1	Eukaryotic translation initiation factor 3 subunit B	1.506692609	0.068144035	S119-only in naïve	K430-0.48（0.005）；K364-0.77(0.005);K595-1.28(0.3);K209-0.64(0.16);K345-0.52(0.0001)	
EIF3C;EIF3CL	B4DVQ5;A1KYQ7;A0A024QYU9;Q99613;B4DVU3;B4DDN4;B4DRU0;B5ME19;B4E2Z6;B3KNZ4;A0A024QYX7	Eukaryotic translation initiation factor 3 subunit C;Eukaryotic translation initiation factor 3 subunit C-like protein	1.624175256	0.014072251	S39-0.79(0.23)	K331-0.905(0.22);K513-0.52(0.088);K712-0.55(0.27)	
EIF3D	O15371;B4E1K8	Eukaryotic translation initiation factor 3 subunit D	1.041372647	0.846666654	/	K426-0.74(0.01);K275-Only in primed	
EIF3E	Q6IAX5;P60228;B2R806;B3KW56;E5RGA2	Eukaryotic translation initiation factor 3 subunit E	1.253335019	0.28927602	S110-Only in primed	/	
EIF3F	B4DMT5;B3KSH1;O00303;B4DEW9	Eukaryotic translation initiation factor 3 subunit F	1.827371695	0.005231017	/	/	
EIF3G	O75821;K7EL20;Q6IAM0;A8K5K5;K7ENA8	Eukaryotic translation initiation factor 3 subunit G	1.014654048	0.95514802	S42-0.64(0.009);T41-Only in naïve	K212-Only in primed;K71-0.79(0.21)	
EIF3H	O15372	Eukaryotic translation initiation factor 3 subunit H	/	/	/	K227-0.43(2.2E-05)	
EIF3I	Q5U0F4;Q53HU7;Q13347	Eukaryotic translation initiation factor 3 subunit I	1.084133736	0.571946409	/	K224-0.92(0.09);K17-Only in naïve	
EIF3K	Q9UBQ5;K7ERF1;K7ES31;U3LUI4;A0A087WVB9;B4DVD7;K7EQM4	Eukaryotic translation initiation factor 3 subunit K	1.341074754	0.045600742	/	K140-1.24(0.13)	
EIF3L	B0QY89;Q9Y262;B4DQF6;B3KPB9;B0QY90;B3KNG0;Q8N7H0	Eukaryotic translation initiation factor 3 subunit L	1.060330897	0.796711065	/	K549-0.77（0.03）;K465-0.63(0.08);K356-0.68(0.0065)	
EIF3M	Q7L2H7;J3KNJ2;H0YCQ8	Eukaryotic translation initiation factor 3 subunit M	1.770035184	0.033657004	/	K254-1.1(0.59)	T117 FHGMDKNTPVRYTVY
EIF3S1;EIF3J	A0A024R5S5;O75822	Eukaryotic translation initiation factor 3 subunit J	0.75871505	0.429752746	S11-0.73(0.31);S13-0.96(0.84)	/	
EIF3S3;EIF3H	Q6IB98;Q53HR0;Q53HG0;B3KS98;O15372;Q5BKY2;A0A087WZK9;E5RJT0	Eukaryotic translation initiation factor 3 subunit H	0.869927514	0.719463605	S180-0.88(0.24)	/	
EIF4A1	A8K7F6;P60842;A8K088;J3KT12;Q59F68;J3QS69;J3KTB5;B4DNH2;J3QL43	Eukaryotic initiation factor 4A-I	1.565449515	0.128759985	S4-Only in primed	K309-0.66(0.02);K174-1.14(0.32);K291-0.97(0.67)	
EIF4A2	Q14240;E7EQG2;Q9NZE6	Eukaryotic initiation factor 4A-II;Eukaryotic initiation factor 4A-II, N-terminally processed	2.007311686	0.007208158	/	/	
EIF4A3	P38919	Eukaryotic initiation factor 4A-III;Eukaryotic initiation factor 4A-III, N-terminally processed;Eukaryotic initiation factor 4A-I;Eukaryotic initiation factor 4A-II;Eukaryotic initiation factor 4A-II, N-terminally processed	/	/	/	K60-0.93（0.92）	
EIF4B	B4DEP6;E7EX17;B4DRM3;P23588;F8VX11;F8VP89;F8VSC7	Eukaryotic translation initiation factor 4B	1.235562544	0.545379963	S502-0.34(0.038);S509-0.25(0.003);S93-Only in primed;S503-0.36(0.068）；S425-Only in primed;S406-0.38(0.087);T420-2.27(0.38);S494-0.24(0.00041);S409-0.44(0.067)	K586-0.08(0.0001)	S340 KLNLKPRSTPKEDDS
EIF4E	Q32Q75;X5D7E3;D6RBW1;P06730	Eukaryotic translation initiation factor 4E	1.571069721	0.116709785	/	/	
EIF4E2;tmp_locus_9	C9JEL3;B9A044;B8ZZ50;Q59FE1;Q53RG0;O60573;B4E1E4;B9A023;B8ZZL3	Eukaryotic translation initiation factor 4E type 2	0.691771802	0.294039788	/	/	
EIF4EBP1	Q13541	Eukaryotic translation initiation factor 4E-binding protein 1	/	/	S5/S65/T46-Only in primed;T70-0.017(0.022)	K57-Only in primed	T70 RNSPVTKTPPRDLPT;S83 PTIPGVTSPSSDEPP;S96 PPMEASQSHLRNSPE
EIF4EBP2	Q6FG68	Eukaryotic translation initiation factor 4E-binding protein 2	/	/	T46-0.43(0.006)	/	
**EIF4ENIF1**	B1AKL4	Eukaryotic translation initiation factor 4E transporter	/	/	S353-0.65(0.15);S540-0.81(0.35);S77-1.32(0.01);S78-1.32(0.01)	/	S44 DIKELPHSKQRPSCL; S82 GRSSPVESLKKELDT; S700 SPSFTPTSVIRKMYE;T755 DRDSSPTTNSKLSAL; S951 RPSQRSSSPVGLAKW
EIF4G1	E7EX73;E9PGM1;E7EUU4	Eukaryotic translation initiation factor 4 gamma 1	1.004905404	0.984281303	S1046-Only in naïve;S1075-1.45(0.24);S1024-1.34(0.04);S1068-0.56(0.02);S1022-1.24(0.78);S1433-0.33(0.14);S1031-Only in primed	K1513-0.8590.02）;K925-1.58(0.23)	
EIF4G2;AAG1	P78344;D3DQV9;Q59G42;B4DZF2;H0Y3P2;Q2TU89;H0YCH5	Eukaryotic translation initiation factor 4 gamma 2	0.876627637	0.255897225	T470-1.77(0.007);S395-0.86(0.28)	K49-Only in primed	T89 RGILNKLTPEKFDKL
EIF4G3	A0A0A0MSA7	Eukaryotic translation initiation factor 4 gamma 3	/	/	S1192-0.82(0.51);S494-0.64(0.063);S231-Only in primed	/	S214 STPVTAASDQKQEEK; T508 IAITVPKTWKKPKDR; T618 ESWKPTDTEGKKQYD
EIF4H;WBSCR1;LOC392647	Q15056;Q75MU1;B4DMV6;Q75MU2;A4D198	Eukaryotic translation initiation factor 4H	1.236282238	0.412427585	/	K80-Only in primed	
EIF5	A0A024R6Q1;P55010;Q6IBU0;H0YLZ1;Q32Q19;Q05DF3;H0YN40	Eukaryotic translation initiation factor 5	1.912469973	0.020479776	/	K95-0.69(0.006)	T153 PENSDSGTGKKEKEK
EIF5A;EIF5AL1	P63241;I3L397;I3L504;Q6IS14	Eukaryotic translation initiation factor 5A-1;Eukaryotic translation initiation factor 5A;Eukaryotic translation initiation factor 5A-1-like	1.425744437	0.144187679	/	K47-10.1(0.03);K50-Only in primed;K68-0.63（0.39）；K85-0.5(0.0006);K67-Only in primed	S44 PCKIVEMSTSKTGKH
EIF5B	Q8N5A0;A0A087WUT6;O60841;B3KM86;D3DVI5	Eukaryotic translation initiation factor 5B	1.337332677	0.143546684	S113-0.64(0.008);S164-0.53(0.004);S214-0.43（0.004）；S137-0.65(0.032);S107-0.57(0.08);S190-1.28(0.68);S135-0.79(0.2);Y134-Only in primed;Y183-Only in naïve;S1168-0.56(0.01)	/	S40 KEQEPQKSKGKKKKE; S295 DTGVIPASEEKAETP
EIF6	P56537	Eukaryotic translation initiation factor 6	0.748830149	0.261475949	/	/	
GC20;EIF1B	Q6FG85;O60739;Q53F41	Eukaryotic translation initiation factor 1b	1.523630314	0.014006418	/	/	

confidence of prediction was ranked as: dark red > red > blue

Table S6-upregulated proteins in naïve containing IRES, Related to Figure 7

UP IN NAÏVE	eGFP_expression (a.u)	UP IN NAÏVE	eGFP_expression (a.u)	UP IN NAÏVE	eGFP_expression (a.u)	UP IN NAÏVE	eGFP_expression (a.u)	UP IN NAÏVE	eGFP_expression (a.u)
SERPINB9		COPS6		CRYZ		HIBCH		HEL107	
UBE1		ANXA7		MYO1E	206.29	MAT2B		HSPA4	
IFITM1		SYAP1	206.29	AF1Q		PSMC5	206.29	HEL-S-69	
ACAA1		CRMP1		TUBB3	206.29	TALDO1		HEL-S-77p	
FN1	1058.1	GLOD4		UBAP2L	206.29	PSMC2		CSNK1E	206.29
HDLBP		TMEM41B		EIF4A2	206.29	PDHB		CCT4	
DFFA	206.29	ZC3H15		PDK3		EEF2	206.29	MSH6	206.29
CORO1B		PTMA	206.29	UBR4		PSMB3		KIF11	
HEL-S-270		COL18A1		P4HA1	206.29	UBA2		COPA	
WARS	206.29	PRIC295		ECM29		MAP1LC3B		DKFZp761E1322	
PBEF1		RPL26L1	1484	DKFZp667H197		PSMC4	206.29	HEL32	
BTBD14B		FABP5		HDGFRP2		SUGT1		UBE2G1	
ARFGEF1		HMGB3		LSM14A		XPO7		HEL2	
LAMC1		MAP4	206.29	RCN3		NUCB1	593.86	TUBA1B	967.52
CSPG2		SQLE	206.29	WIBG		MAP1S		TUBB2C	
MAP1B		NPEPPS		ARMT1		PSMD10		HEL-S-100n	
RECQL	206.29	RPL10	213.1	MRS2	206.29	PFDN2		PFKP	
PTGES3		LAMB1	206.29	HN1	206.29	TARS	1063.5	NSUN2	
CTPS2		USP11	967.52	NSFL1C		WAPAL		ACACA	206.29
EPPK1		DHRS7		PLAA		EIF3F		MAD2L1	
PRODH	206.29	TXN		DRG1	206.29	CCT3	206.29	EIF3A	206.29
LYPLA1		ARHGDIA	206.29	ACOT9		GJA1		TRIP12	
C21orf33	206.29	L1RE1		COPG1		GRN		PSMD6	1869.5
CAPNS1		SCD		PSMC3	206.29	USP7		PDK2	206.29
FXR1	206.29/1181.8	MGEA5		EIF3C	206.29	YWHAZ	1233.5	DRIP4	
NLRP2		WDHD1	2075.9	ALDOC		POU5F1		WDR1	
ANXA6		HEL-S-133P		SAE1		S100A10		CCT7	206.29
PLIN3	1110.1	HEL-S-68p		ARF1		TWF2		UTF1	
USP5		HEL-S-102		LASP1	206.29	EZR		EXOC4	
TLN1		CAPN1	894.26	VAT1		GNB2L1	206.29	EIF3M	
TCEA1		ENO2		UBE2E2		HSPA8	353.84	SERBP1	206.29
RAD50	206.29	HEL-S-22		RPL15	1057.4	TUBA1C	483.16	PFDN5	206.29
FABP3		HEL-S-165mP		SUCLG2		FAM162A	206.29	FARSB	
DKFZp667O202		HEL-S-30		PARP1		OTUB1		TBL1XR1	
LGALS1	206.29	PFKL		EEF1B2		EL52		ACO2	206.29
TAGLN2	206.29	ARF4	206.29	LRRC47		PSMD11	1339.2	APRT	
SEP-2		PGAM1		hCG_2003792		CLIC1	206.29		
CLU	206.29	LAMA1		EIF5	206.29	IGF2BP3			
CBR1	206.29	HMGB2	206.29	SDCBP		SNX3			
TTC13		DDT	206.29	DARS2		GC20			
PRDX1		PGM1	206.29	MAGED2	206.29	GPC4			
KIF1A		STAT3		RCC2		PFN1			
TBCB		UTRN	1581	IQGAP1	206.29	PLS3	682.93		
SART3		SARS	206.29	NAP1L4		TRA1			
ESYT2		ALDH3A2	206.29	CCT6A		TCP1	206.29		
GSPT1	206.29	SMS		BCAM		GLDC			
TMEM199		HEL-S-49		GARS		ACP1			
PFAS		COPS2		TPM1		RPS12	206.29		
PRPS1		GLO1	206.29	DKFZp686J1372		DNAJB1			
ARPC3	206.29	MCL1	373.72	PSMA5	206.29	EEF1G	206.29		

Table S7-qPCR Primer Sequences, Related to Figure 1 and Figure 3

Gene Name	Forward primer	Reverse primer
ACTB	F-AACCGCGAGAAGATGACCCA	R-GGATAGCACAGCCTGGATAGCA
ARGFX	F-TCGACCTACCGGAGTCAACAG	R-GGAGAAAAAGCATAAGGACTGGG
B3GAT1	F-CTCCTTCGAGAACTTGTCACC	R-GGGTCAGTGAAGCCCTTCTT
BMP4	F-CACTGGCTGACCACCTCAAC	R-GGCACCCACATCCCTCTACT
BRACHYURY	F-TATGAGCCTCGAATCCACATAGT	R-CCTCGTTCTGATAAGCAGTCAC
CD130	F-GAAGGTGGGAAGGATGGTCC	R-CAGGCACGACTATGGCTTCA
CD7	F-CCACAGCCCCAAGACATCATT	R-GGTGCATGGTGATAGTCAGGTT
CDH1	F-CGAGAGCTACACGTTCACGG	R-GGGTGTCGAGGGAAAAATAGG
CDK1	F-AAACTACAGGTCAAGTGGTAGCC	R-TCCTGCATAAGCACATCCTGA
CK18	F-AGCTCAACGGGATCCTGCTGCACCTTG	R-CACTATCCGGCGGGTGGTGGTCTTTTG
DNMT3L	F-TCTCAAGCTCCGTTTCACCC	R-ACTTGTCCTTACATGGGGCG
eIF3A	F-GCCGGAAAATGCCCTCAAAC	R-TGGTTCGTGTATCTTTTGCCAT
eIF4A1	F-ATGGCACTAGGAGACTACATGG	R-CCACGGCTTAACATTTCGTCA
eIF4A2	F-CCAAAAGGTAATTCTGGCACTTG	R-CGGGTGTACCAACAACAATATGT
FOXA2	F-GGAGCAGCTACTATGCAGAGC	R-CGTGTTCATGCCGTTCATCC
GAPDH	F-GGAAGGTGAAGGTCGGAGTC	R-GAAGGGGTCATTGATGGCAAC
GATA4	F-TCCCTCTTCCCTCCTCAAAT	R-TCAGCGTGTAAAGGCATCTG
GATA6	F-TCCACTCGTGTCTGCTTTTG	R-TCCTAGTCCTGGCTTCTGGA
GBX2	F-AGCGAGGTGCAGGTGAAAAT	R-GCTGCTGATGCTGACTTCTGA
HERVH	F-GCCTCTGCTCCTCCACCCTATAA	R-CGTTTAGCTCCAGCCACCTTTTT
HES4	F-GAGCGCGTATTAACGAGAGC	R-GCAGGTGTCTCACGGTCATC
KHDC1L	F-GACTTGATGACACGTACCTTCG	R-AGCGTGACACTTGGAGTCCT
KLF17	F-GGGATGGTGCGATAGATTCA	R-GCCTCACCCTCACCTAACAA
KLF4	F-TCAACCTGGCGGACATCAAC	R-CAGCACGAACTTGCCCATCA
KLF5	F-TCAGACAGCAGCAATGGACACTC	R-GTGGCCTGTTGTGGAAGAAACTG
MAP2	F-CAGGTGGCGGACGTGTGAAAATTGAGAGTG	R-CACGCTGGATCTGCCTGGGGACTGTG
MEF2C	F-TTTAACACCGCCAGCGCTCTTCACCTTG	R-TCGTGGCGCGTGTGTTGTGGGTATCTCG
NANOG	F-CTGTGATTTGTGGGCCTGAAGAA	R-TTTGGGACTGGTGGAAGAA
NKX2.5	F-AAGTGTGCGTCTGCCTTTCCCG	R-TTGTCCGCCTCTGTCTTCTCCA
NKX3.1	F-CCATACCTGTACTGCGTGGG	R-TGCACTGGGGGAATGACTTA
PAX6	F-TGGGCAGGTATTACGAGACTG	R-ACTCCCGCTTATACTGGGCTA
POU5F1	F-GTGGAGGAAGCTGACAACAA	R-ATTCTCCAGGTTGCCTCTCA
PRDM14	F-GCATACTCCGCACACACATCA	R-CAGCCATCATCCTCCTTGTGT
SOX17	F-GTGGACCGCACGGAATTTG	R-GGAGATTCACACCGGAGTCA
SOX2	F-CATGGGTTCGGTGGTCAAGTC	R-TCGGCGCCGGGGAGATACA
STELLA	F-CAGCAGGAGAGGAGTAAGAACA	R-TGAAGTGGCTTGGTGTCTTGA
TFCP2L1	F-GCCCTTTCGAGTCCAGATTGA	R-CTCCTTCTCTTGGGCAGTTCTT
ZIC2	F-GCGCAACTCCACAACCAGTA	R-TGCCGCATATAGCGGAAAAAG

Data S1. Uncropped Western blot, Related to Figures 3, 6, 7, S5, S6, S8 and S10

Fig 7A

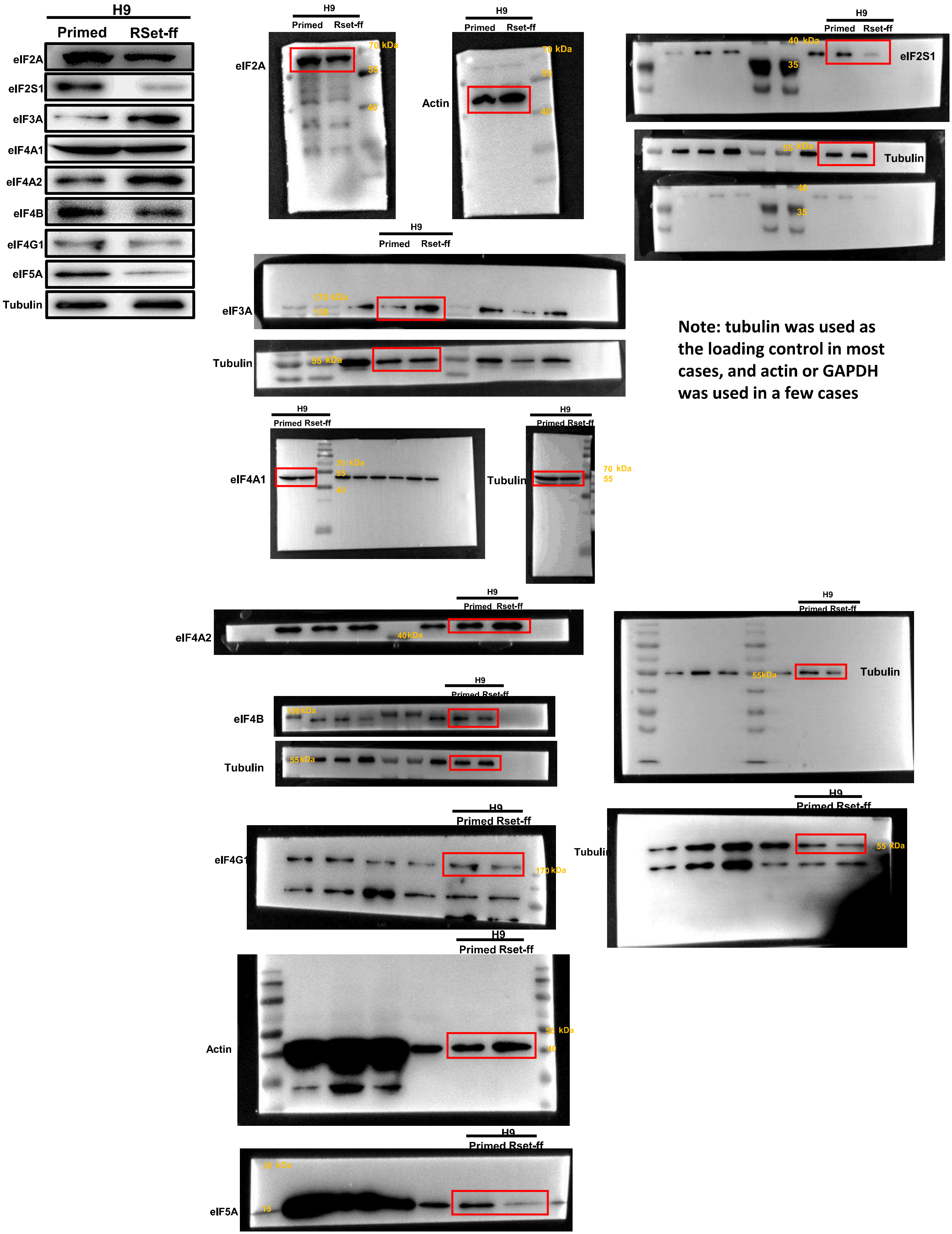

Note: tubulin was used as the loading control in most cases, and actin or GAPDH was used in a few cases

Fig S10A

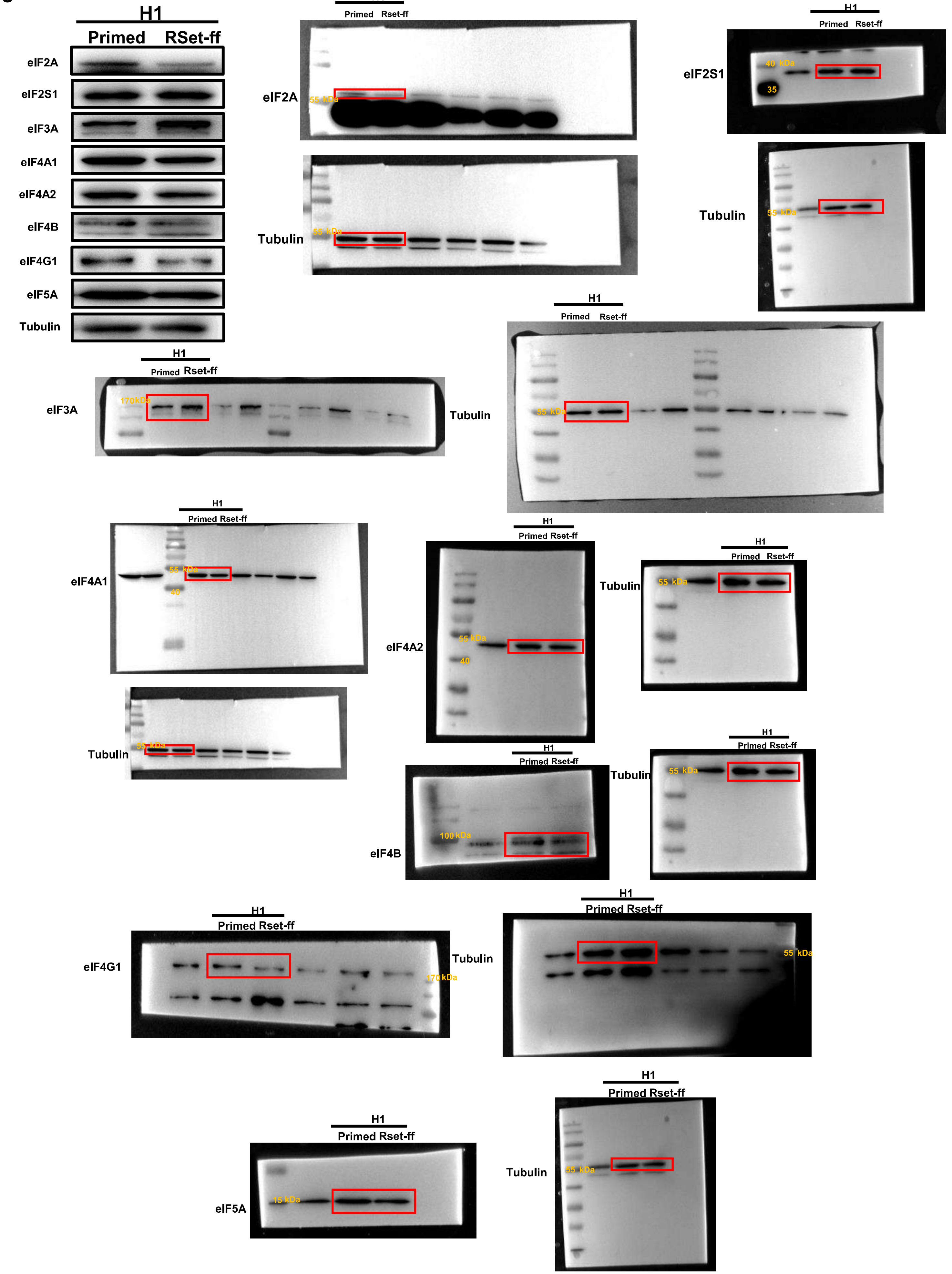
H1
Primed
RSet-ff
eIF2A
eIF2S1
eIF3A
eIF4A1
eIF4A2
eIF4B
eIF4G1
eIF5A
Tubulin
Rset-ff
55 kDa
40 kDa
35
170 kDa
40
100 kDa
15 kDa

Fig 3B

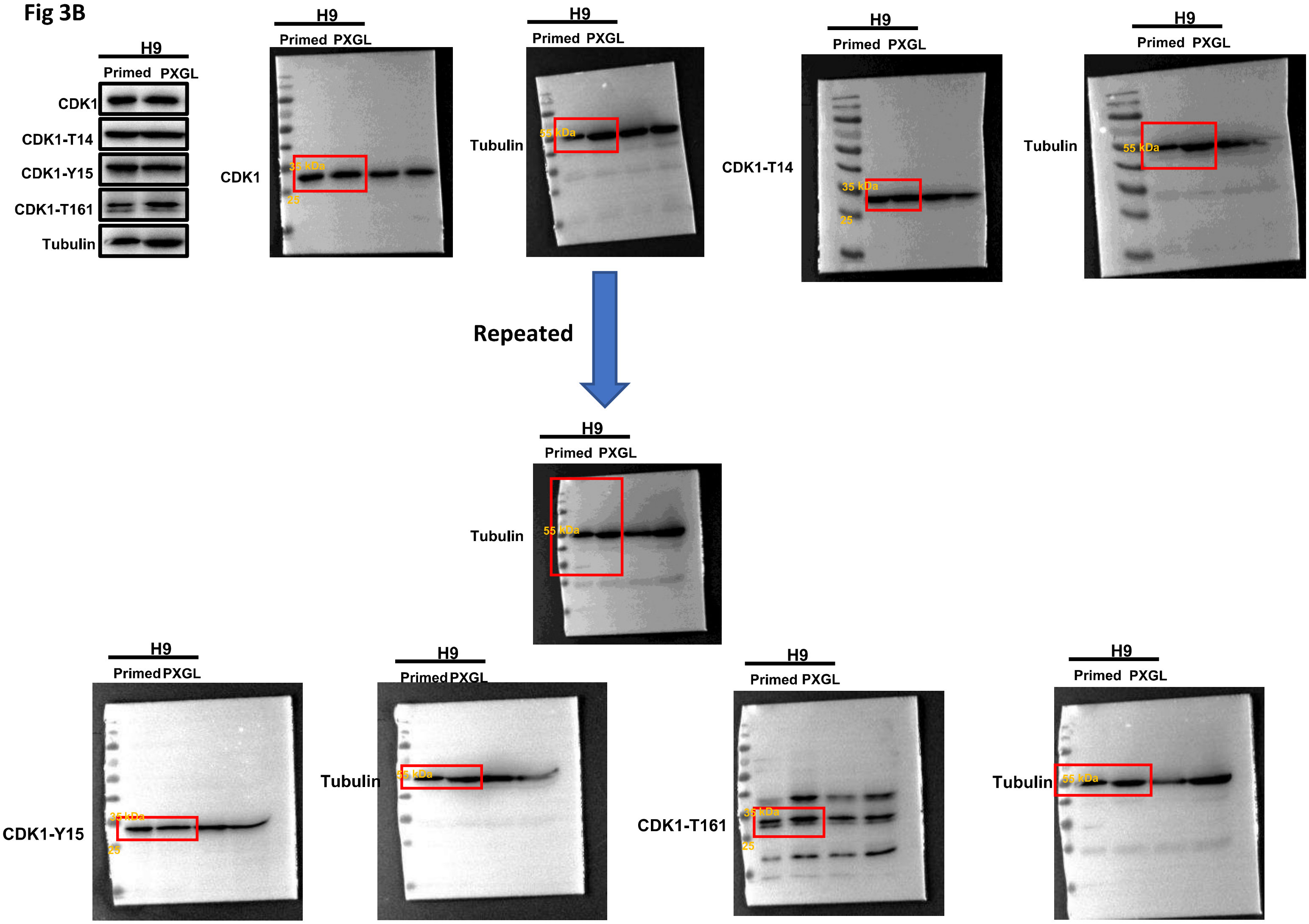
H9
Primed PXGL
CDK1
CDK1-T14
CDK1-Y15
CDK1-T161
Tubulin
Repeated

Fig 3F

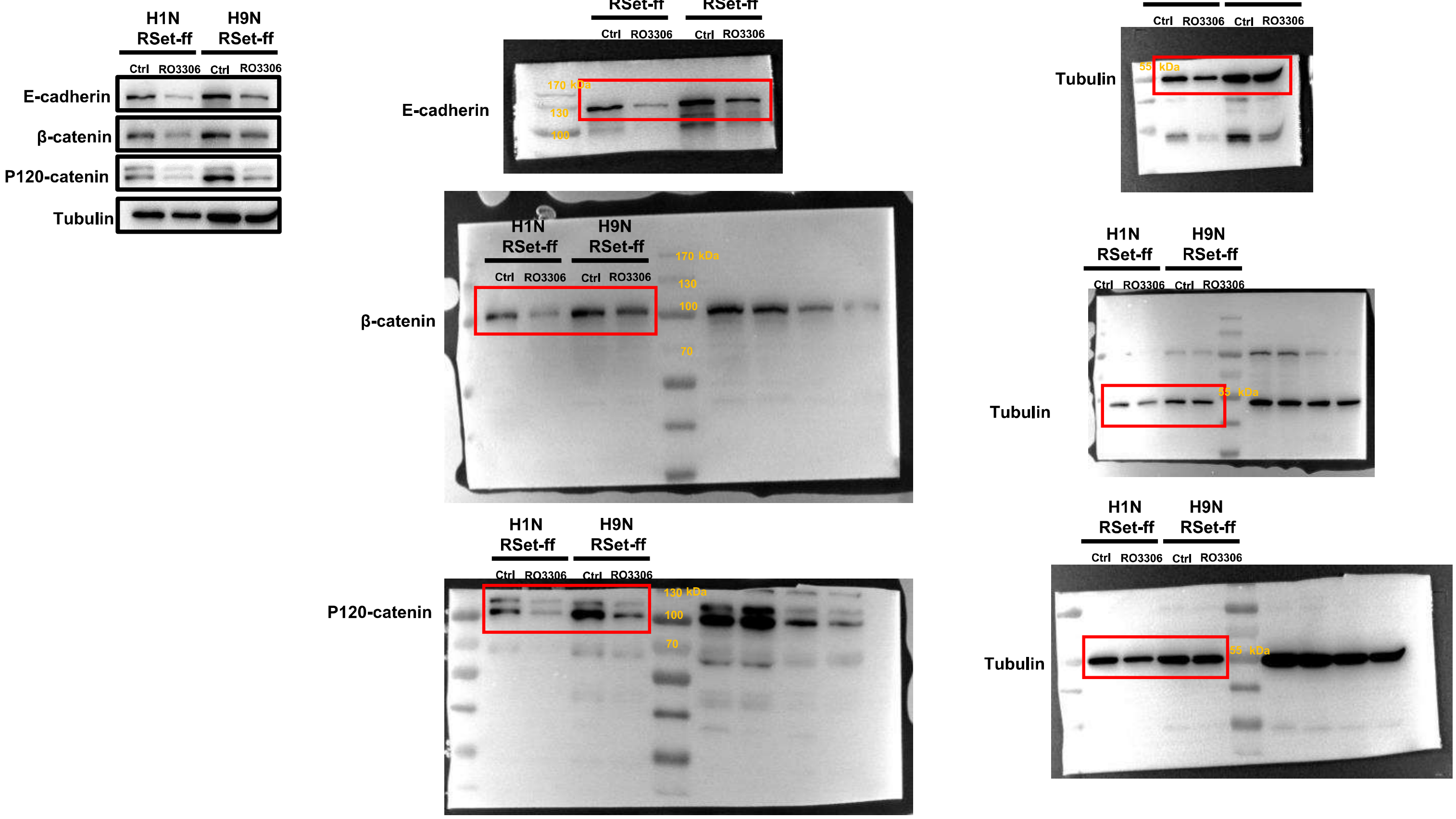
H1N RSet-ff
H9N RSet-ff
Ctrl RO3306 Ctrl RO3306
E-cadherin
β-catenin
P120-catenin
Tubulin
170 kDa
130
100
70
55 kDa

Fig 3H

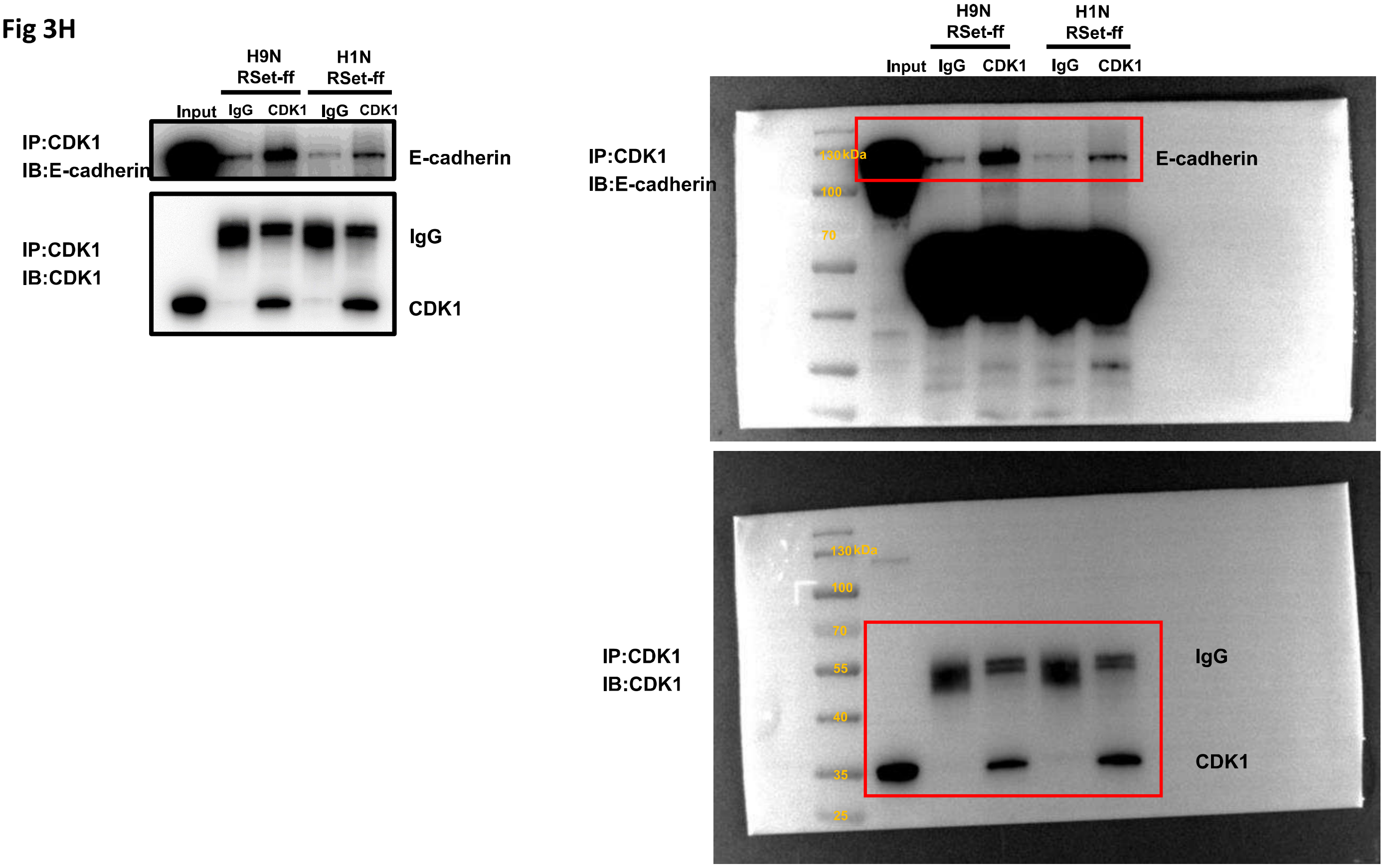
H9N RSet-ff
H1N RSet-ff
Input IgG CDK1 IgG CDK1
IP:CDK1
IB:E-cadherin
E-cadherin
IP:CDK1
IB:CDK1
IgG
CDK1
130 kDa
100
70
55
40
35
25

Fig 3K

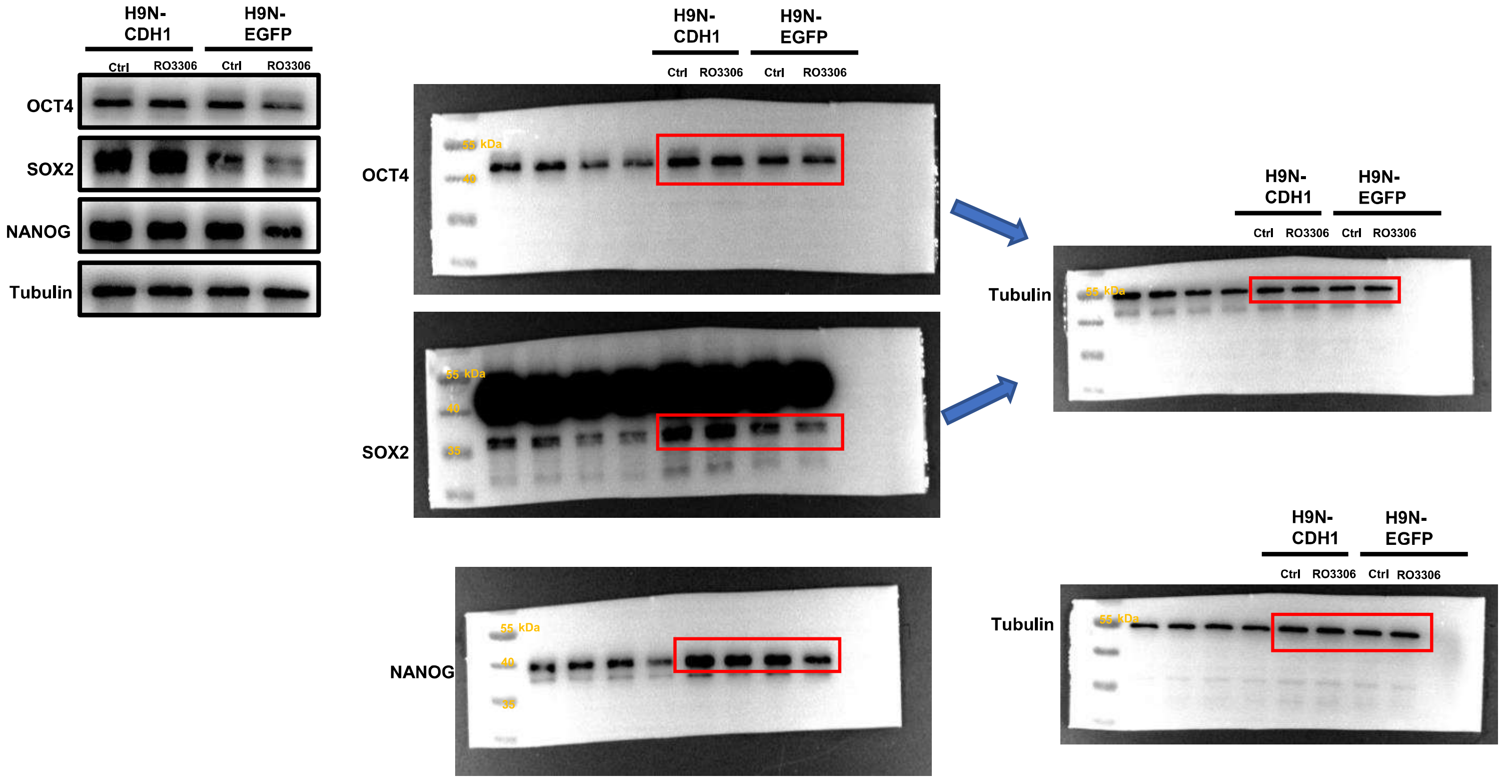
H9N-CDH1
H9N-EGFP
Ctrl
RO3306
Ctrl
RO3306
OCT4
SOX2
NANOG
Tubulin
55 kDa
40
35

Fig 6C

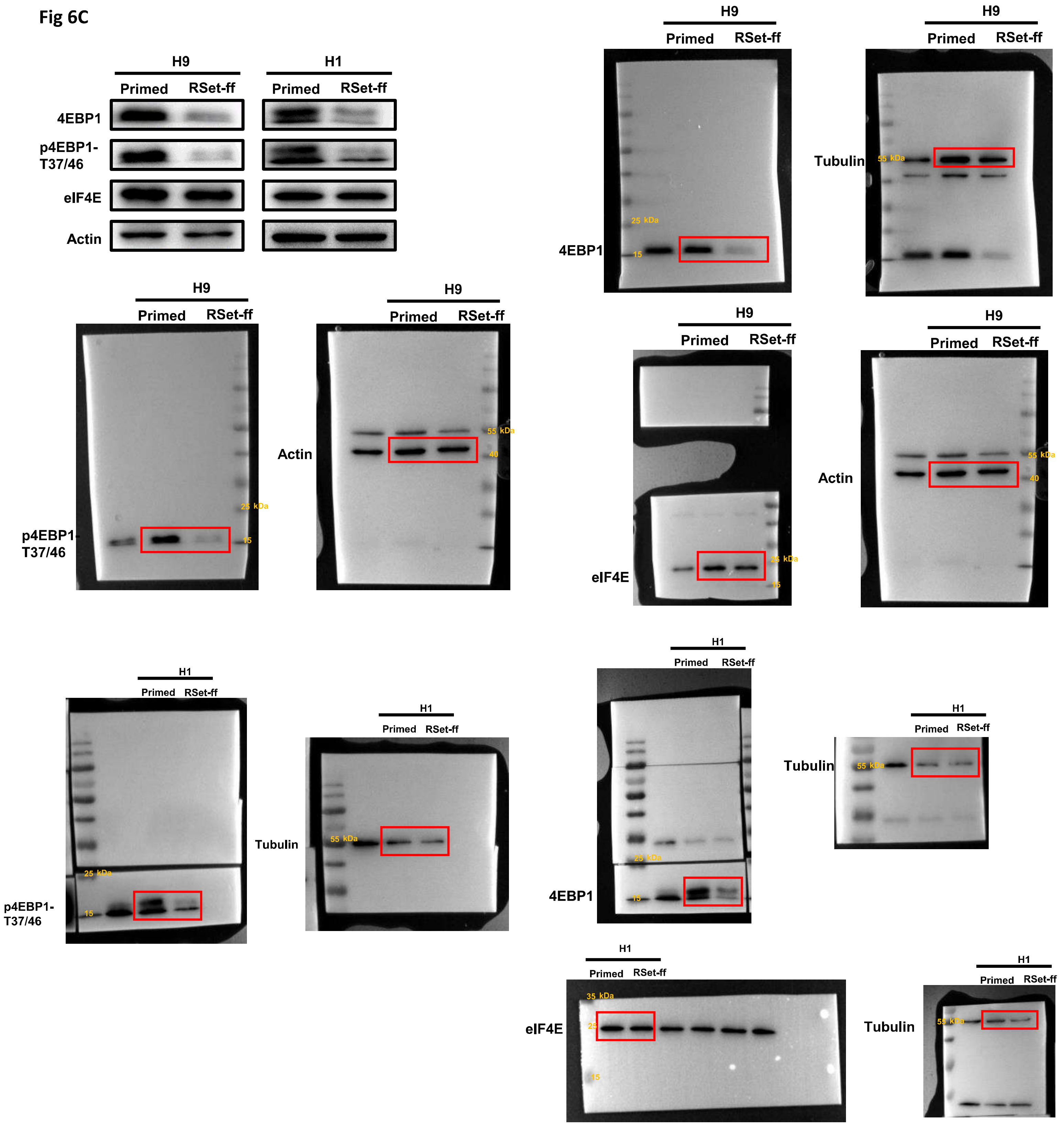
H9
H1
Primed
RSet-ff
4EBP1
p4EBP1-
T37/46
eIF4E
Actin
Tubulin
55 kDa
40
25 kDa
35 kDa
25
15

Fig 6D

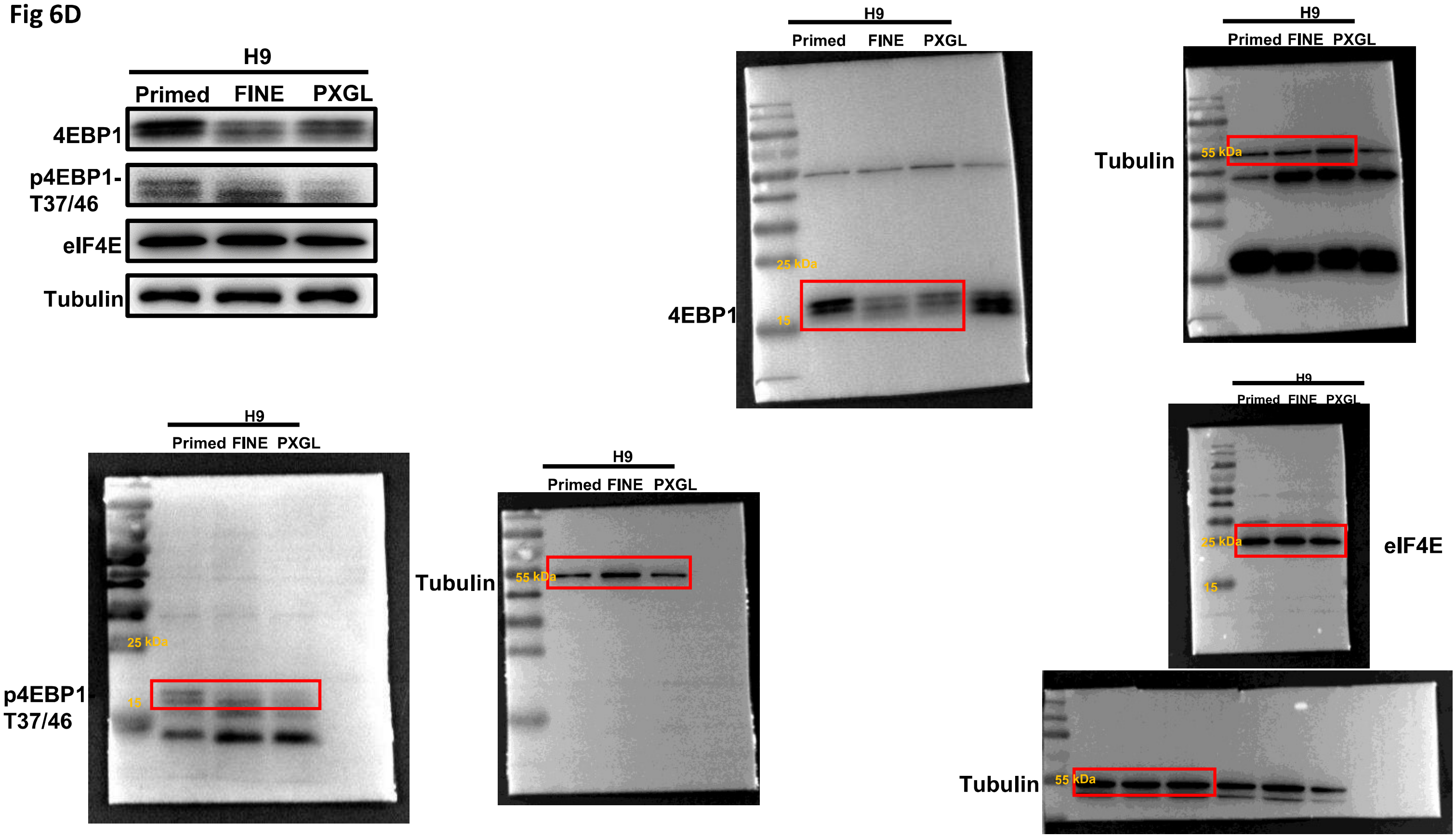
H9
Primed
FINE
PXGL
4EBP1
p4EBP1-T37/46
eIF4E
Tubulin
25 kDa
15
55 kDa
p4EBP1 T37/46

Fig 7C

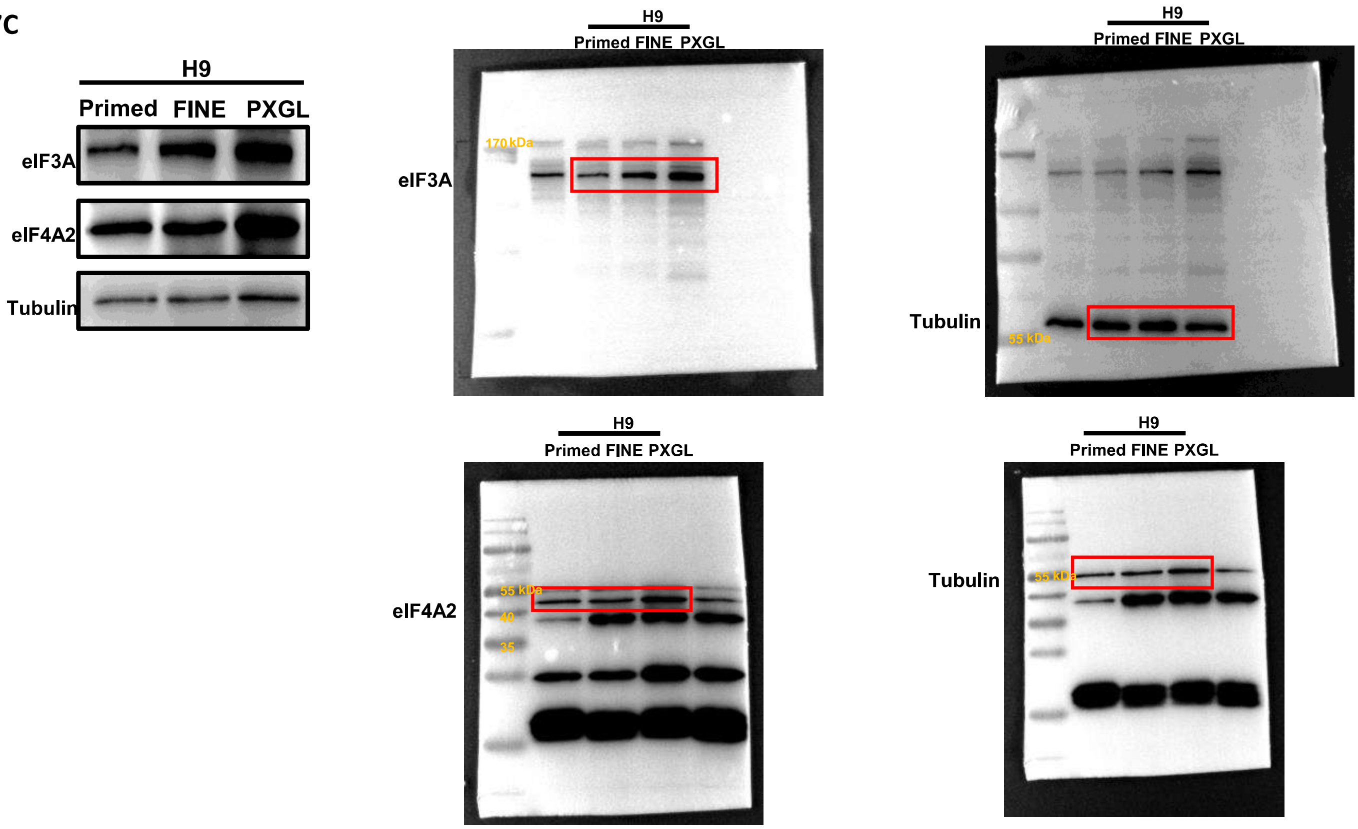
H9
Primed
FINE
PXGL
eIF3A
eIF4A2
Tubulin
170 kDa
55 kDa
40
35

Fig S5A

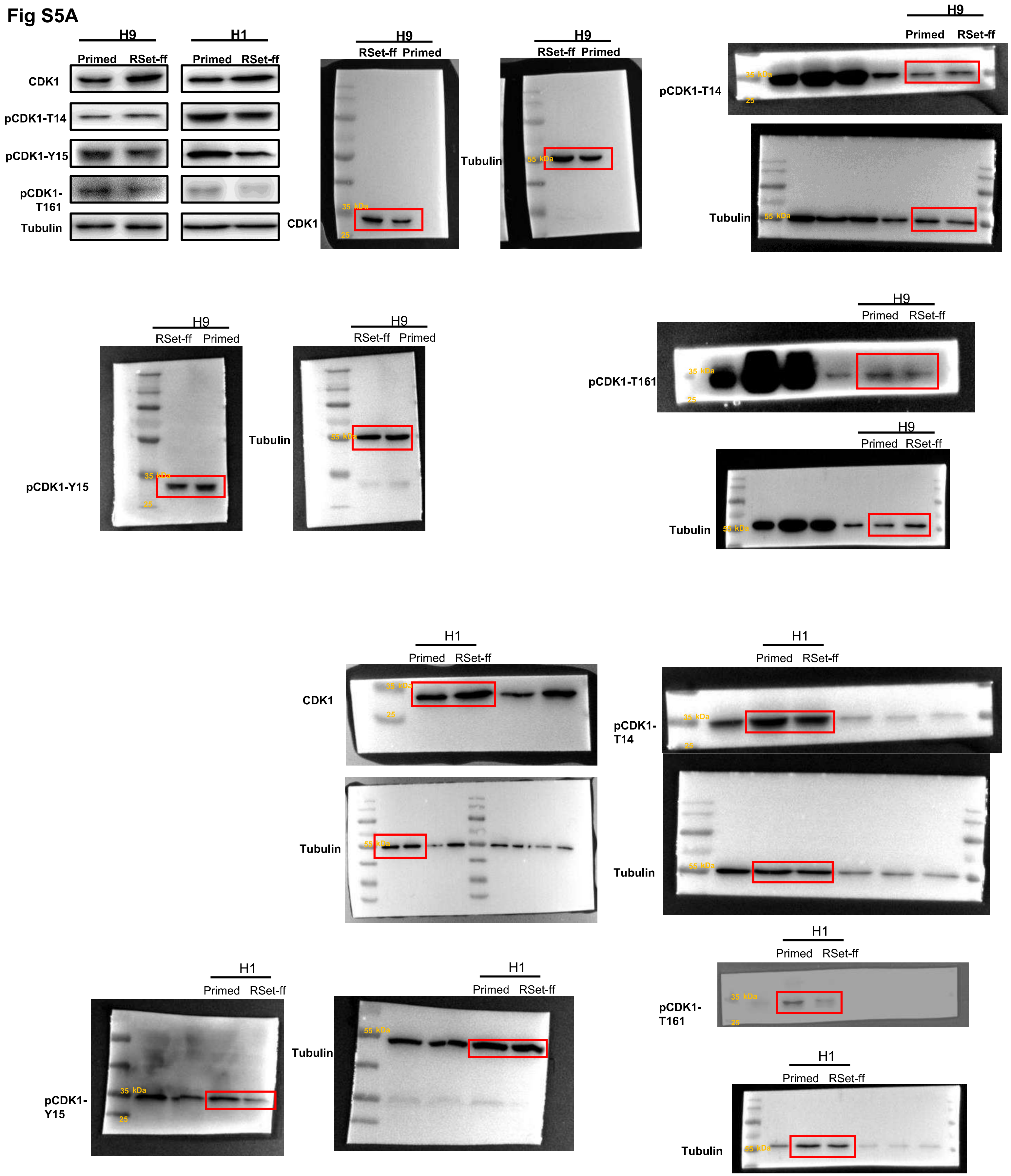
H9
H1
Primed
RSet-ff
CDK1
pCDK1-T14
pCDK1-Y15
pCDK1-T161
Tubulin
RSet-ff
Primed
35 kDa
25
55 kDa

Fig S5E

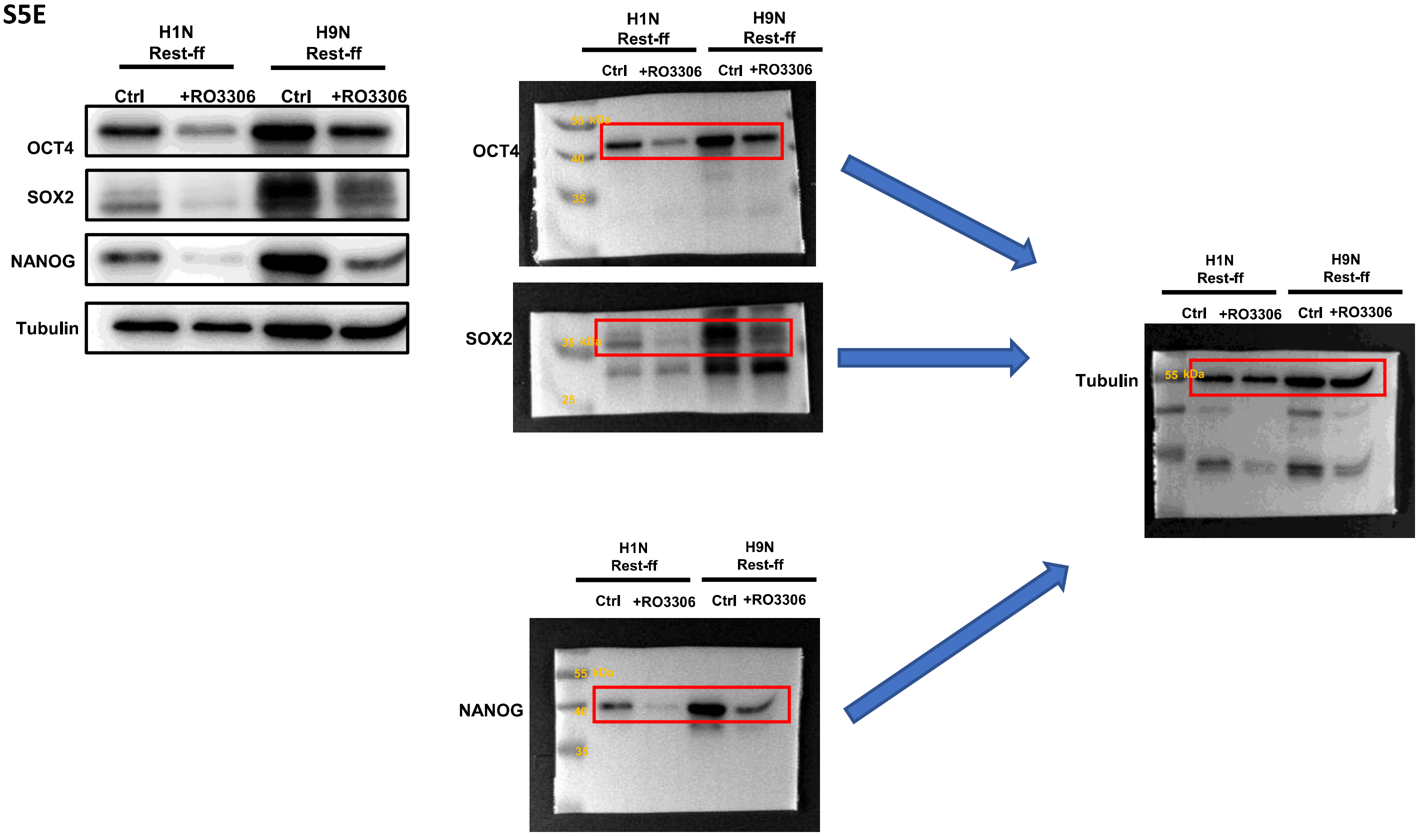

Fig S6C

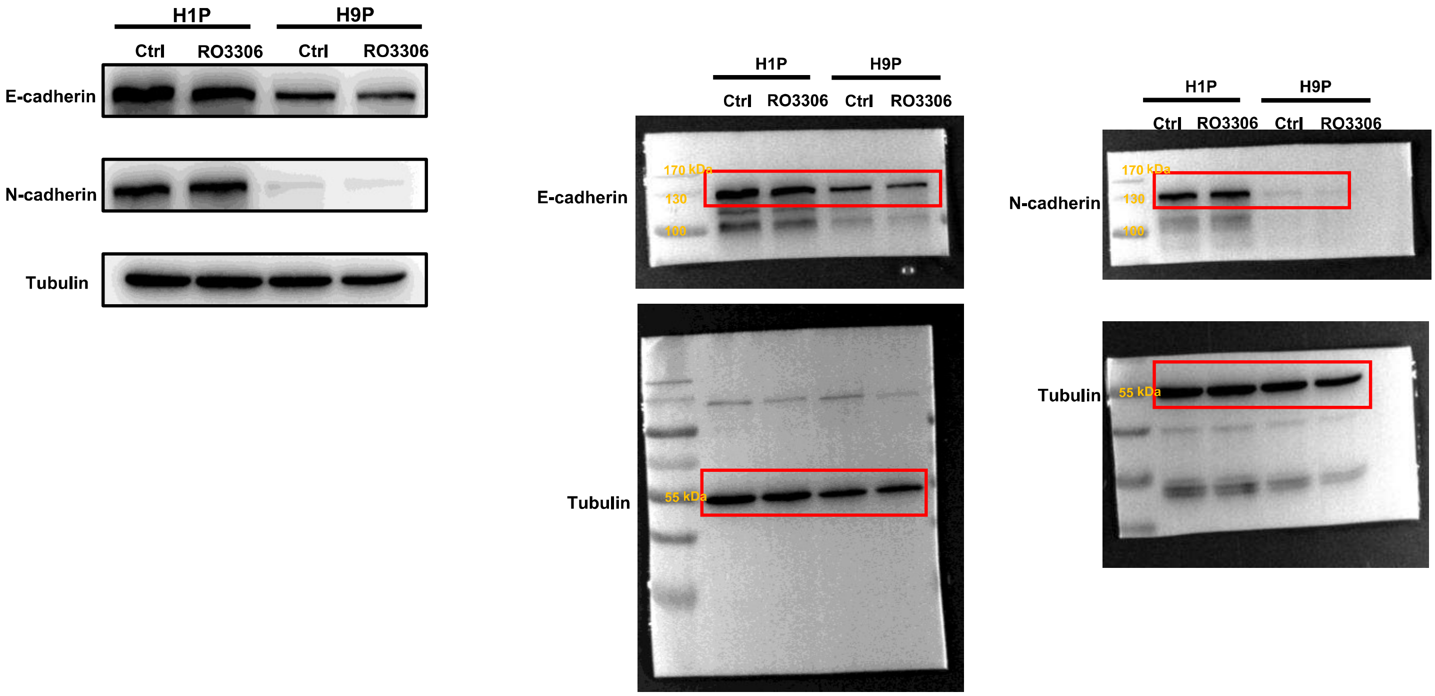

Fig S6D

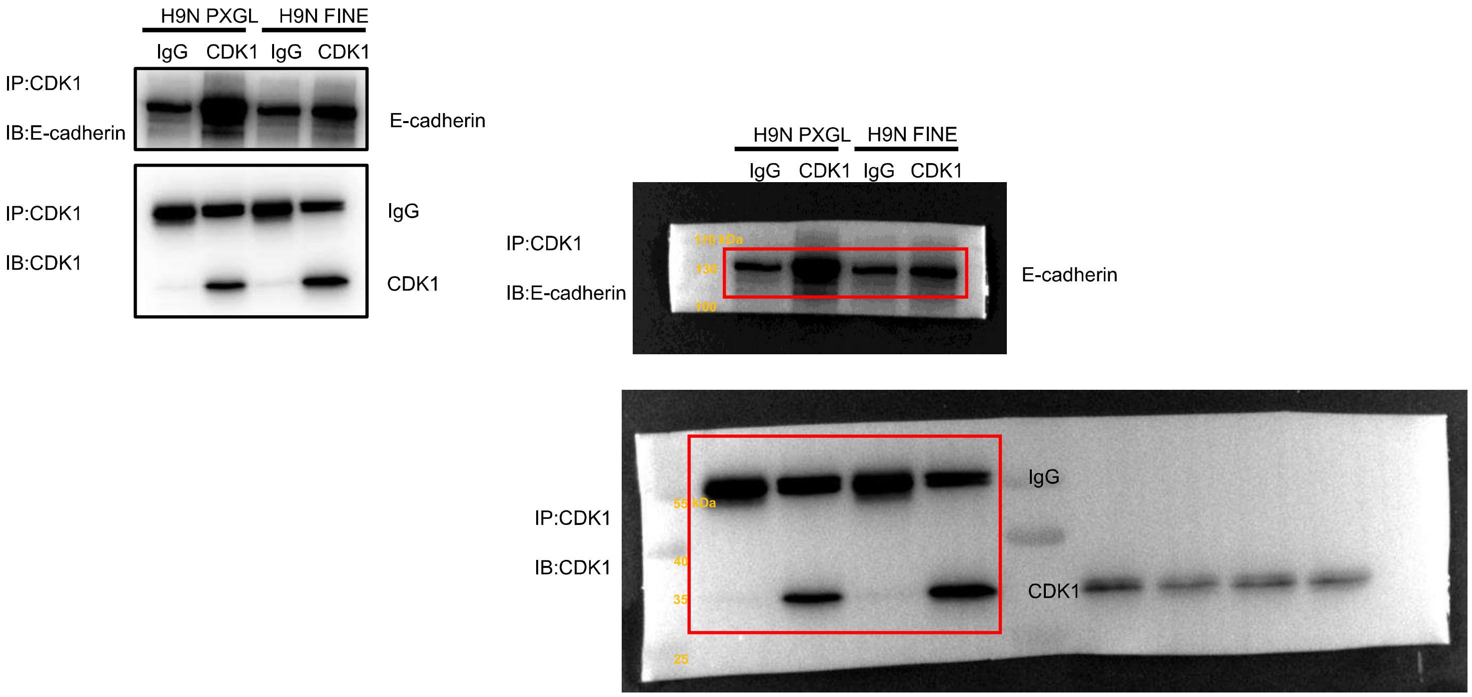

Fig S6E

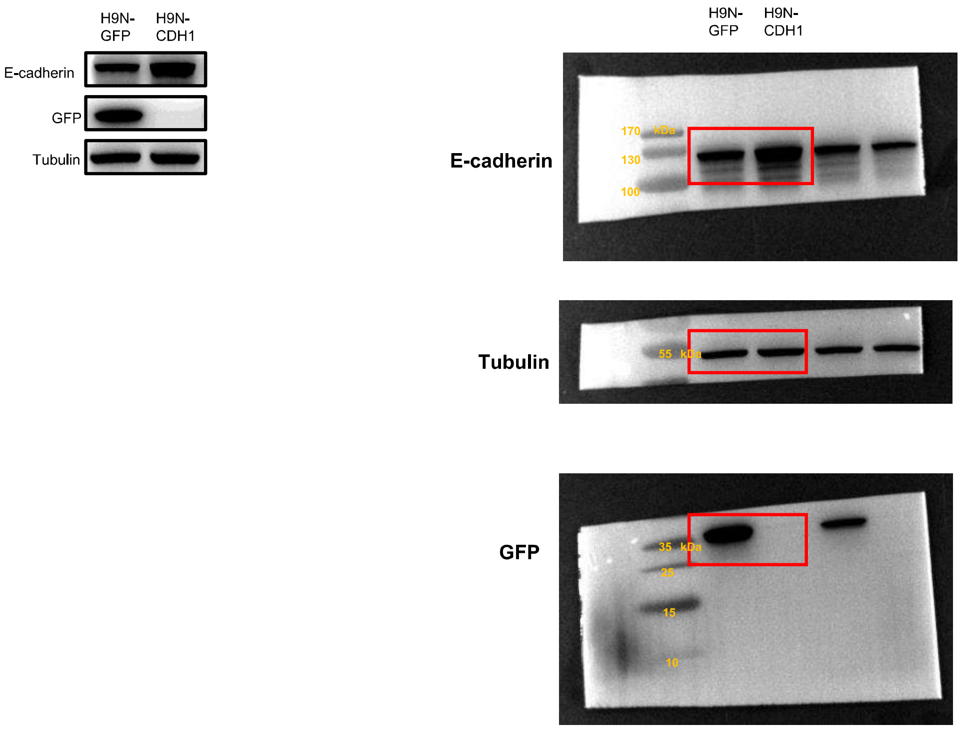

Fig S8D

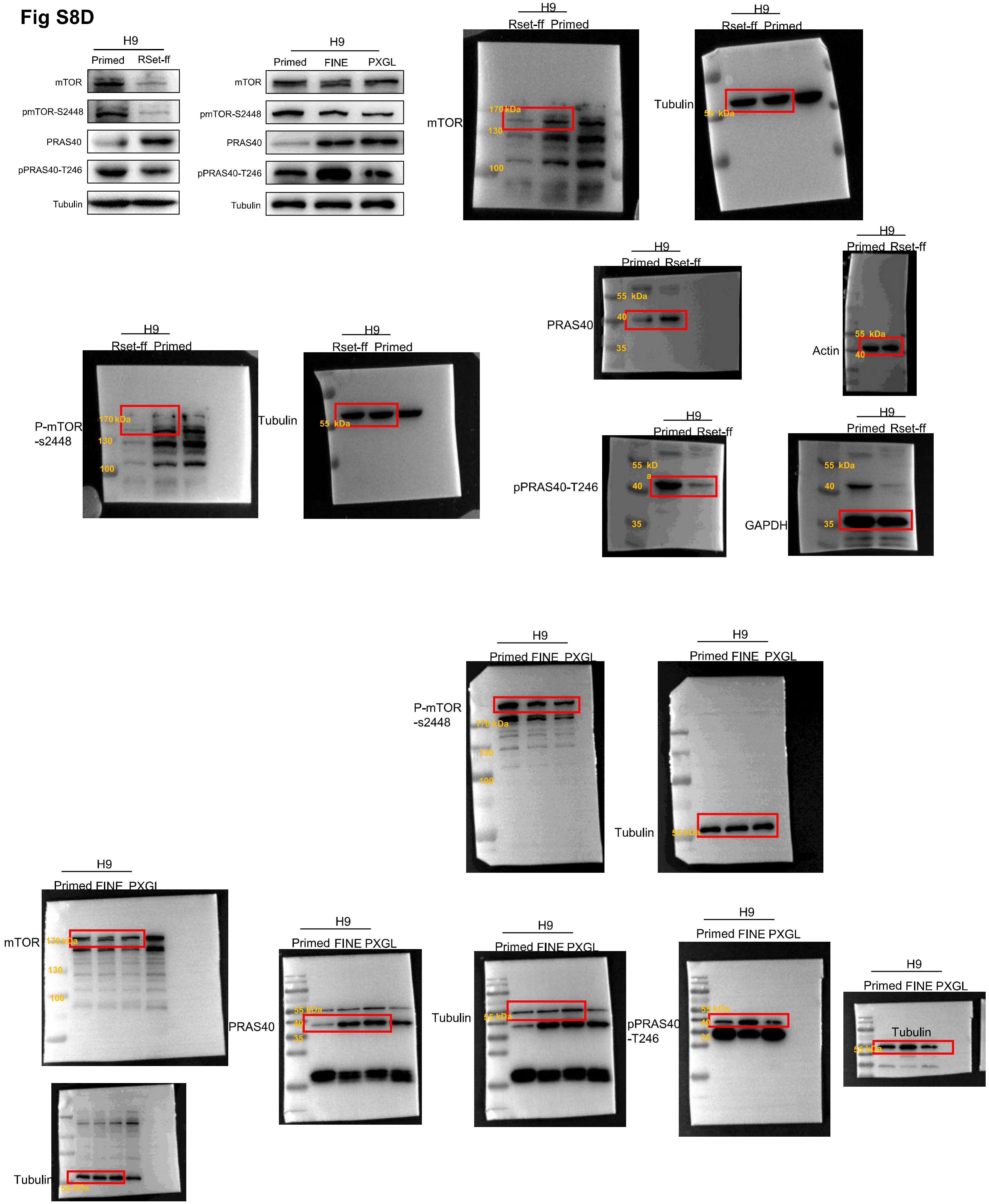

H9
Primed
RSet-ff
mTOR
pmTOR-S2448
PRAS40
pPRAS40-T246
Tubulin
FINE
PXGL
Rset-ff Primed
170 kDa
130
100
55 kDa
Primed Rset-ff
40
35
Actin
P-mTOR -s2448
GAPDH
Primed FINE PXGL
pPRAS40 -T246

Fig S8E

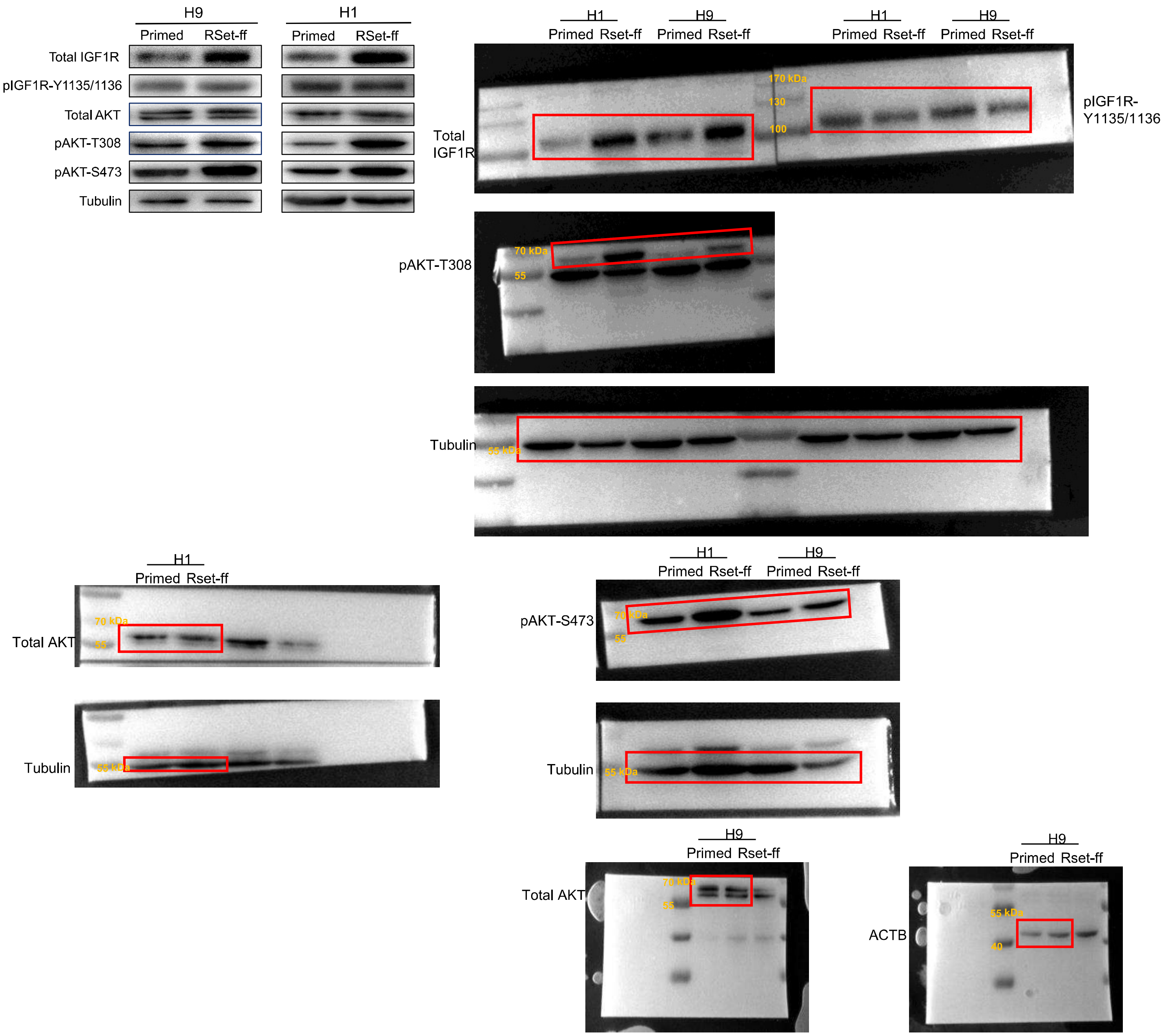

Fig S10C

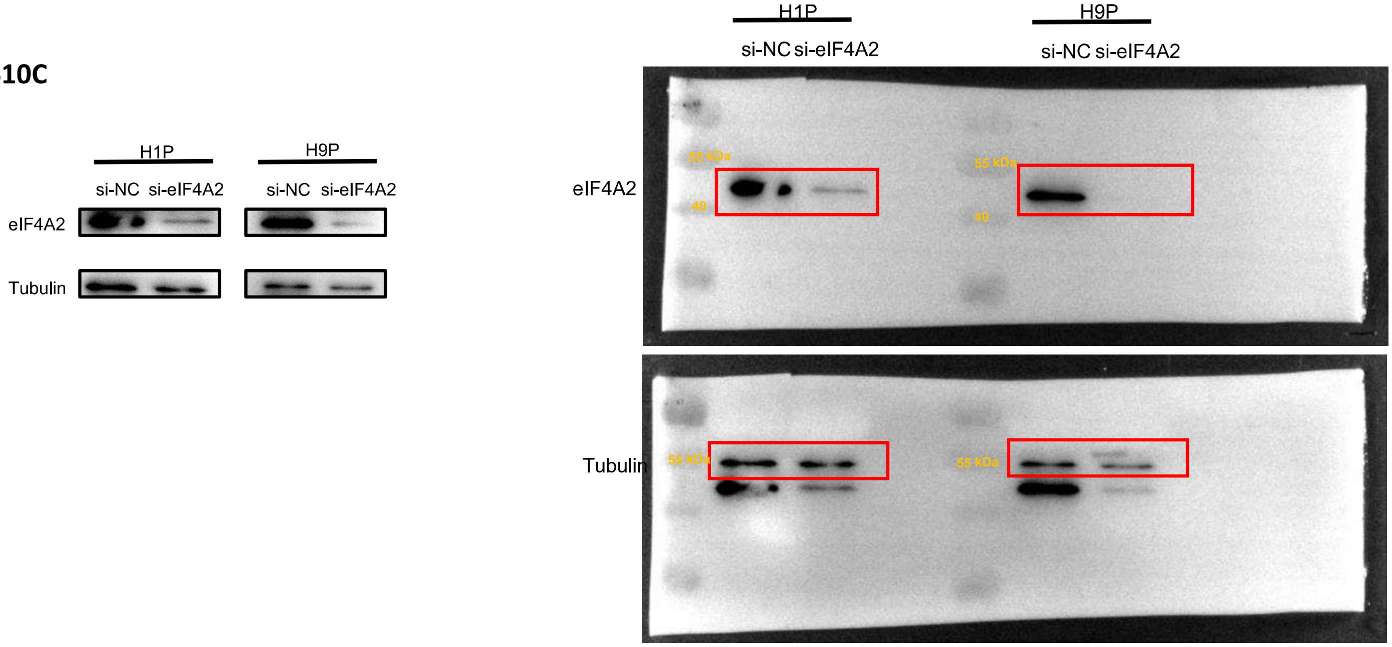

Supplement: Document S1. Figures S1–S10, Data S1, and Table — S5–S7 [file mmc1.pdf]
